# Discovery of VU6007496: Challenges in the Development of an M_1_ Positive Allosteric Modulator Backup Candidate

**DOI:** 10.1021/acschemneuro.4c00508

**Published:** 2024-08-28

**Authors:** Julie L. Engers, Katrina A. Bollinger, Rory A. Capstick, Madeline F. Long, Aaron M. Bender, Jonathan W. Dickerson, Weimin Peng, Christopher C. Presley, Hyekyung P. Cho, Alice L. Rodriguez, Colleen M. Niswender, Sean P. Moran, Zixiu Xiang, Anna L. Blobaum, Olivier Boutaud, Jerri M. Rook, Darren W. Engers, P. Jeffrey Conn, Craig W. Lindsley

**Affiliations:** † Warren Center for Neuroscience Drug Discovery, Vanderbilt University, Nashville, Tennessee 37232, United States; ‡ Department of Pharmacology, Vanderbilt University School of Medicine, Nashville, Tennessee 37232, United States; § Department of Chemistry, Vanderbilt University, Nashville, Tennessee 37232, United States; ∥ Vanderbilt Kennedy Center, Vanderbilt University Medical Center, Nashville, Tennessee 37232, United States; ⊥ Vanderbilt Brain Institute, Vanderbilt University, Nashville, Tennessee 37232, United States; # Vanderbilt Institute of Chemical Biology, Vanderbilt University, Nashville, Tennessee 37232, United States

**Keywords:** muscarinic acetylcholine receptor subtype 1 (M_1_), positive allosteric modulator (PAM), cognition, metabolism

## Abstract

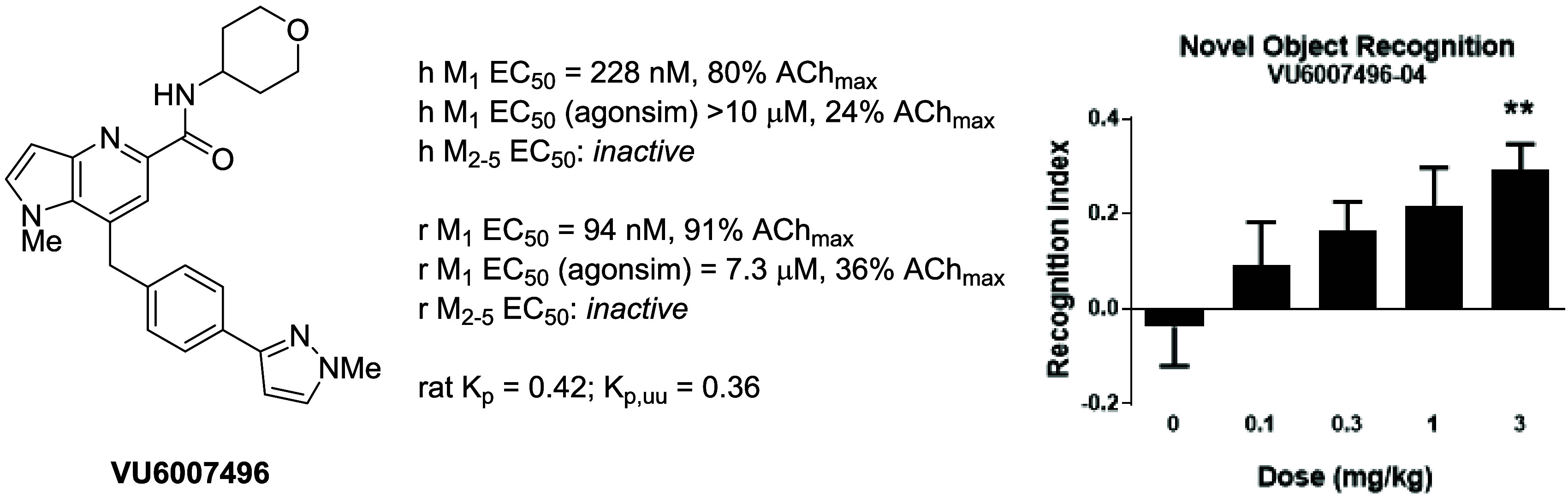

Herein we report progress toward a backup clinical candidate to the M_1_ positive allosteric modulator (PAM) VU319/ACP-319. Scaffold-hopping from the pyrrolo[2,3-*b*]pyridine-based M_1_ PAM VU6007477 to isomeric pyrrolo[3,2-*b*]pyridine and thieno[3,2-*b*]pyridine congeners identified several backup contenders. Ultimately, VU6007496, a pyrrolo[3,2-*b*]pyridine, advanced into late stage profiling, only to be plagued with unanticipated, species-specific metabolism and active/toxic metabolites which were identified in our phenotypic seizure liability *in vivo* screen, preventing further development. However, VU6007496 proved to be a highly selective and CNS penetrant M_1_ PAM, with minimal agonism, that displayed excellent multispecies IV/PO pharmacokinetics (PK), CNS penetration, no induction of long-term depression (or cholinergic toxicity) and robust efficacy in novel object recognition (minimum effective dose = 3 mg/kg p.o.). Thus, VU6007496 can serve as another valuable *in vivo* tool compound in rats and nonhuman primates, but not mouse, to study selective M_1_ activation.

## Introduction

**Figure 1:**
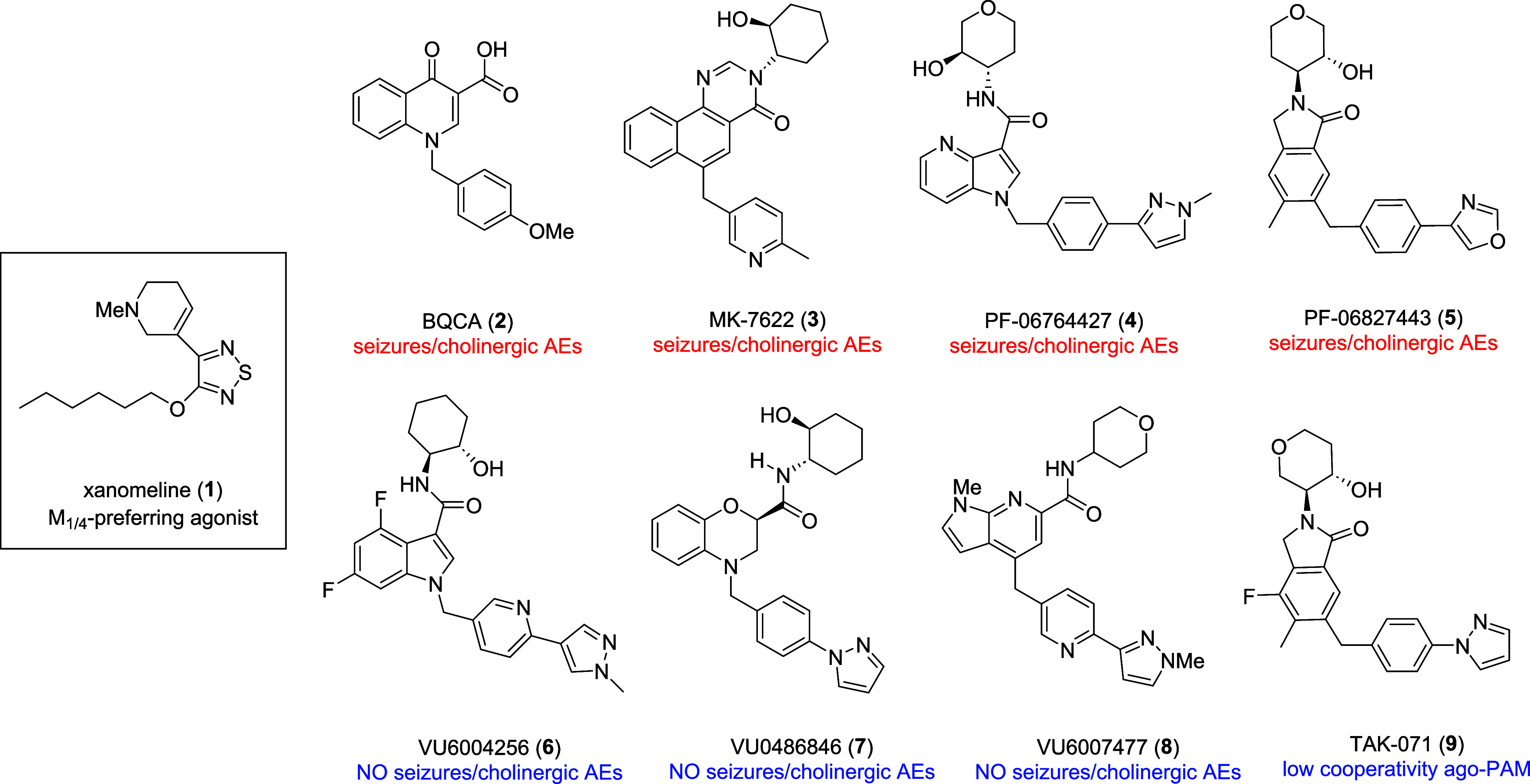
Structures of xanomeline (**1**), representative M_1_ ago-PAMs with cholinergic AEs **2**–**5**, representative “pure” PAMs devoid of M_1_ agonism **6**–**8**, and the low cooperative M_1_ ago-PAM TAK-071 (**9**).

The resurgence of muscarinic acetylcholine receptors (mAChRs or M_1–5_) at the forefront of CNS drug discovery for a variety of neuropsychiatric disorders, driven by the pending FDA approval of xanomeline **1** (an M_1_/M_4_ agonist, combined with a peripheral muscarinic antagonist as KarXT),^1−3^ has focused attention on the development of selective M_1_ and M_4_ positive allosteric modulators (PAMs). While M_4_ PAMs have demonstrated preclinical and clinical efficacy for treatment of the positive symptoms of schizophrenia, M_1_ PAMs offer promise for treating cognitive dysfunction in schizophrenia, Alzheimer’s disease and other CNS disorders.^4−7^ Potent ago-PAMs, such as **2**–**5** (Figure 1),^8−12^ overstimulate the M_1_ receptor and lead to adverse events (AEs) and cholinergic toxicity, which has diminished enthusiasm for the mechanism. However, PAMs with minimal to no M_1_ agonism, such as **6**–**8**,^13−16^ proved devoid of AEs and cholinergic toxicity in preclinical animal models. Interestingly, the M_1_ ago-PAM TAK-071 (**9**),^17,18^ displaying low cooperativity, was efficacious in a number of preclinical rodent models and has advanced into human clinical testing. From our efforts with VU319/ACP-319 (structure not disclosed at this time), an M_1_PAM with no detecable M_1_ agonsim proved devoid of AEs, cholinergic toxicity, and other toxicology findings in rat, dog, and nonhuman primate to support an open IND by the FDA. Recently, safety and pharmacokinetic data from a Phase I single ascending dose (SAD) study for ACP-319 have been reported.^19,20^ This M_1_ PAM was well tolerated, with no cholinergic side effects noted, at doses showing cognitive improvement and functional target engagement. Moreover, pharmacokinetic (PK) data demonstrated that ACP-319 demonstrated good absorption and bioavailability in man with a half-life supporting once daily dosing.^19,20^ Based on these data, the team was charged with developing a chemically orthogonal backup compound to ACP-319. Here, we disclose the chemical optimization of VU6007477 (**8**) and the challenges and tribulations that led to the discovery of not a backup clinical candidate, but a new *in vivo* tool compound, VU6007496.

## Results and Discussion

### Design

**Figure 2:**
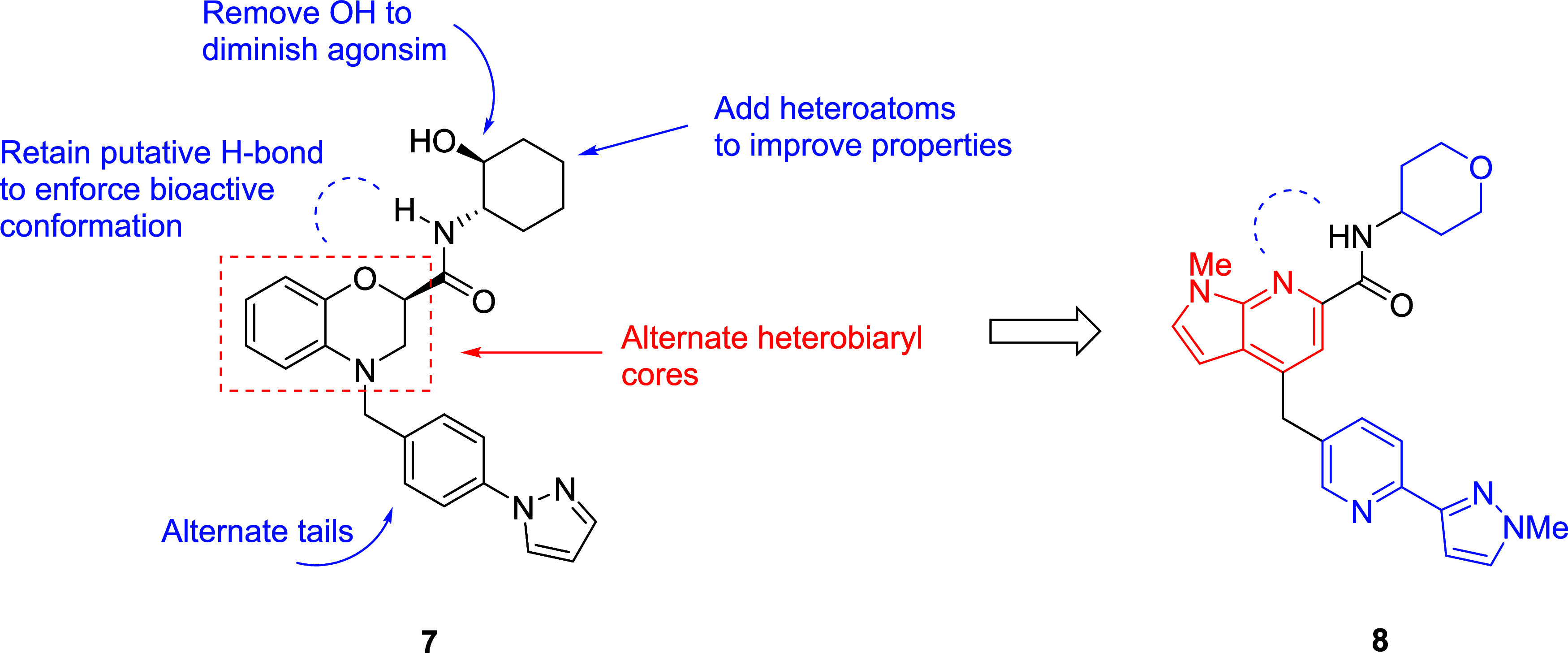
Initial scaffold-hopping exercise from **7** that led to **8**, a novel M_1_ PAM devoid of cholinergic toxicities and AEs.

Our initial backup campaign focused on the chemically orthogonal M_1_ PAM scaffold **7** (VU0486846), devoid of M_1_ agonism, AEs, and cholinergic toxicity, but which exhibited an unacceptable CYP_450_ profile.^15^ We elected to employ a scaffold-hopping approach to replace the benzomorpholine core while surveying alternate amides (deleting the agonism-prone hydroxyl moiety and incorporating heteroatoms) and various southern tail moieties (Figure 2). This exercise led to exceedingly steep structure–activity relationship (SAR) in terms of the generation of M_1_ ago-PAMs (displaying cholinergic side effects) *versus* M_1_ PAMs with minimal to no M_1_ agonism, with a single analog advancing as a new M_1_ PAM *in vivo* tool compound, **8** (VU6007477), with robust precognitive efficacy and no cholinergic toxicity or AEs.^16^ However, the overall profile of **8** did not warrant advancement as a backup candidate to VU-319/ACP-319.

**Figure 3:**
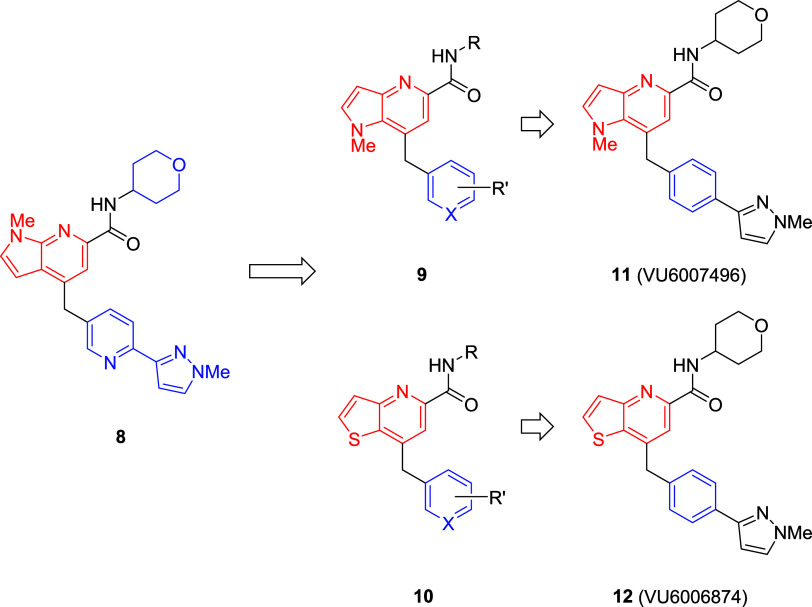
Scaffold-hopping from **8** to regioisomeric cores **9** and **10**, which led to the discovery of VU6007496 (**11**) and VU6006874 (**12**) that were advanced into further profiling.

Far more dramatic departures in subsequent scaffold-hopping campaigns were met with resolute failure to provide M_1_ PAMs devoid of M_1_ agonism, so the team decided to revisit the pyrrolo[2,3-*b*]pyridine-based **8**(16) and explore an alternate regioisomer, namely a pyrrolo[3,2-*b*]pyridine core **9** as well as an isosteric thieno[3,2-*b*]pyridine core **10** (Figure 3).^21^ Here again, SAR proved to be steep and failed to identify M_1_ PAMs with diminished M_1_ agonism worthy of further progression toward backup compounds. This exercise led to the discovery of two compounds that advanced into further profiling, VU6007496 (**11**, a pyrrolo[3,2-*b*]pyridine) and VU6006874 (**12**, a thieno[3,2-*b*]pyridine), with unexpected results and challenges.

### Synthesis

**Scheme 1:**
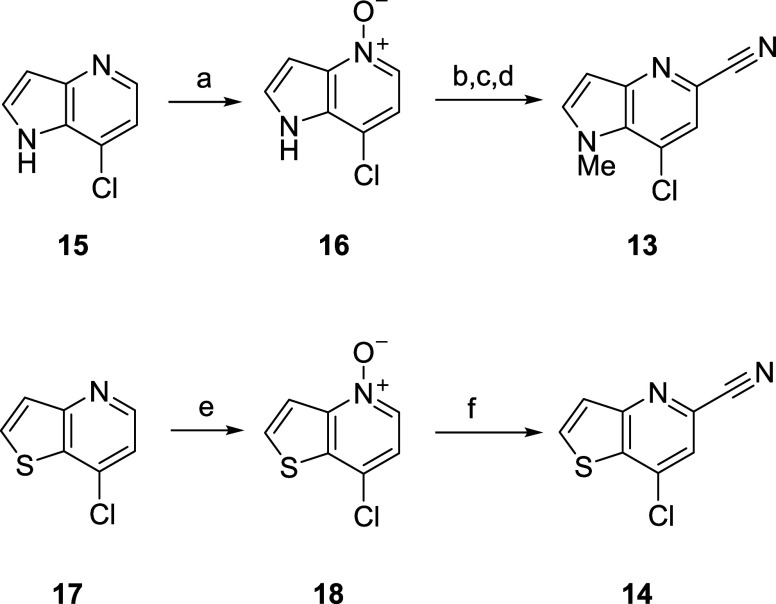
Synthesis of 7-Chloro-1-methyl-1*H*-pyrrolo[3,2-*b*]pyridine-5-carbonitrile **13** and a 7-Chlorothienyl[3,2-*b*]pyrdine-5-carbonitrile **14** Reagents and conditions: (a) *m-*CPBA, *n*-BuOAc:heptane (3:5), 0 °C—rt, 83%; (b) (MeO)_2_SO_2_, *n*-BuOAc, 75 °C, 16 h; (c) KCN, aq. NH_4_Cl, 50 °C, 2 h; quantitative yield (2 steps); (d) MeI, NaH, DMF, 0 °C—rt, 98%; (e) *m-*CPBA, DCM, 0 °C—rt, 54%; (f) TMSCN, (CH_3_)_2_NCOCl, DCM, rt, 16 h, 98%.

The synthesis of **11** and **12** (Scheme 1) first required the construction of key intermediates, namely a 7-chloro-1-methyl-1*H*-pyrrolo[3,2-*b*]pyridine-5-carbonitrile **13** and a 7-chlorothienyl[3,2-*b*]pyrdine-5-carbonitrile **14**.^21^ Here, we envisioned that these key intermediates would be prepared from the commercially available chloropyridines **15** and **17** by application of a Reissert-Henze reaction sequence.^22^ Conversion of **15** to the pyridine *N*-oxide **16** by treatment with *m-*CPBA proceeded in 85% yield. Treatment of **16** with dimethyl sulfate and KCN facilitated the Reissert-Henze reaction followed by *N*-methylation affording the requisite cyanopyridine **13** in 98% over 3 steps. A similar sequence provided the analogous cyanopyridine **14**, in albeit lower overall yield (∼53% over two steps).^21^

**Scheme 2:**
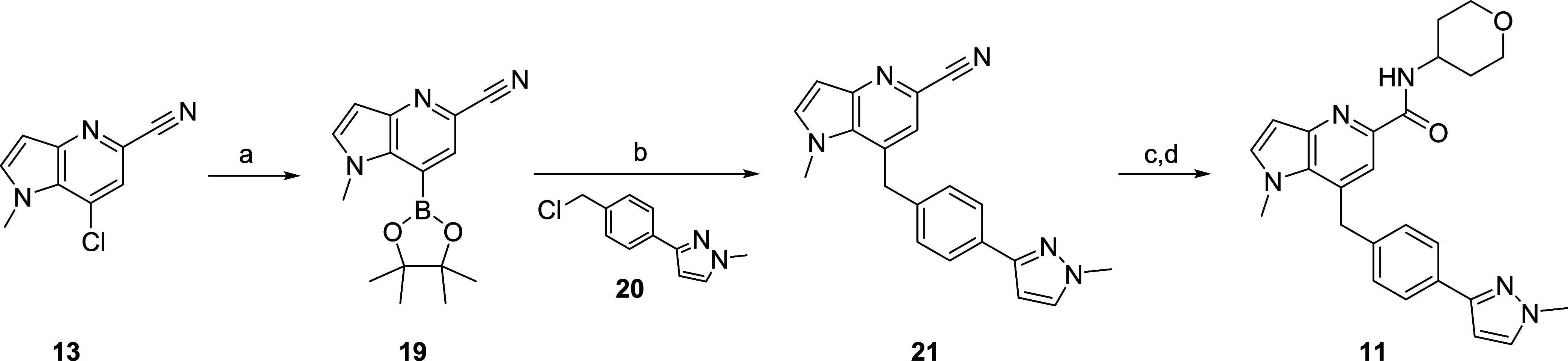
Synthesis of VU6007496 (**11**) Reagents and conditions: (a) bis(pinacolato)diboron, KOAc, Pd(dppf)Cl_2_, 1,4-dioxane, 100 °C, 16 h; (b) benzyl chloride **20**, Cs_2_CO_3_, Pd(dppf)Cl_2_, THF/H_2_O, 90 °C, 16 h; 56% (2 steps); (c) conc. HCl, reflux, 2 h; (d) tetrahydro-2*H*-pyran-4-amine, HATU, DIEA, DMF, rt, 20 min, 71% (2 steps).

With intermediates **13** and **14** in hand, elaboration into the M_1_ PAMs **11** and **12** proved straightforward. Conversion of **13** to the boronate ester **19** proceeded smoothly (Scheme 2), followed by a Suzuki coupling with benzyl chloride **20** to provide **21** in 56% yield for the two steps. Acid-mediated hydrolysis of the nitrile to the carboxylic acid, and a HATU-facilitated coupling with tetrahydro-2*H*-pyran-4-amine delivered **11** in 71% yield for the two step sequence.^21^

**Scheme 3:**
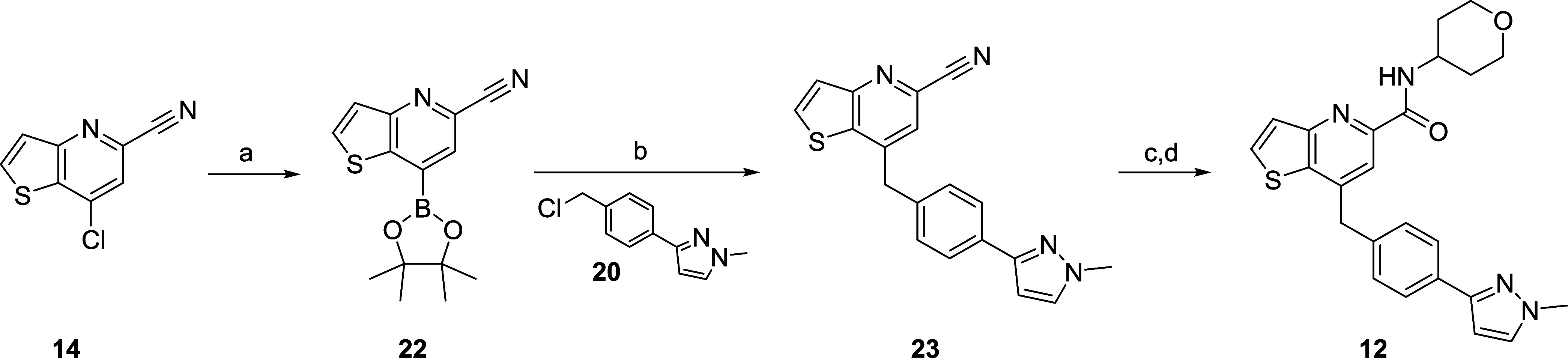
Synthesis of VU6006874 (**12**) Reagents and conditions: (a) bis(pinacolato)diboron, KOAc, Pd(dppf)Cl_2_, 1,4-dioxane, 100 °C, 16 h, 95%; (b) benzyl chloride **20**, Cs_2_CO_3_, Pd(dppf)Cl_2_, THF/H_2_O, 90 °C, 16 h, 99%; (c) conc. HCl, 100 °C, 3 h; (d) tetrahydro-2*H*-pyran-4-amine, HATU, DIEA, DMF, rt, 1 h, 61% (2 steps).

Application of a similar reaction sequence with 7-chlorothienyl[3,2-*b*]pyridine-5-carbonitrile **14** provided **12** (Scheme 3). Here, conversion of **14** to the boronate ester **22** proceeded in excellent yield (95%). A Pd(dppf)Cl_2-_catalyzed Suzuki coupling with benzyl chloride **20** gave the elaborated nitrile **23** in 99% isolated yield. Finally, acid-mediated hydrolysis of the nitrile to the acid, and a HATU-facilitated coupling with tetrahydro-2*H*-pyran-4-amine delivered **12** in 61% yield for the two-step sequence.^21^

**Scheme 4:**
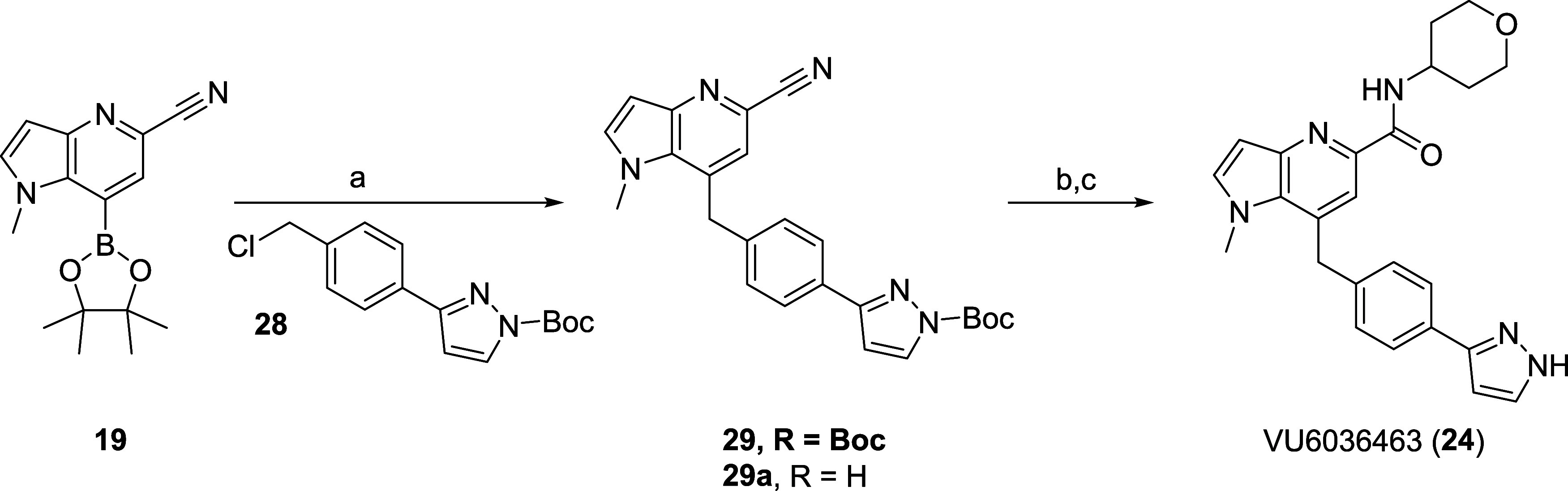
Synthesis of Metabolite VU6036463 (**24**) Reagents and conditions: (a) benzyl chloride **28**, Cs_2_CO_3_, Pd(dppf)Cl_2_, THF/H_2_O, 90 °C, 16 h; 58%; (b) conc. HCl, reflux, 2 h; (c) tetrahydro-2*H*-pyran-4-amine, HATU, DIEA, DMF, rt, 20 min, 81% (2 steps).

### Molecular Pharmacology and *In Vitro* Drug Metabolism and Pharmacokinetics (DMPK)

With **11** and **12** in hand, both compounds were evaluated in a battery of molecular pharmacology and *in vitro* DMPK assays (Table 1) to assess their suitability to advance further down the testing cascade toward putative backup candidates.

**Table 1:**
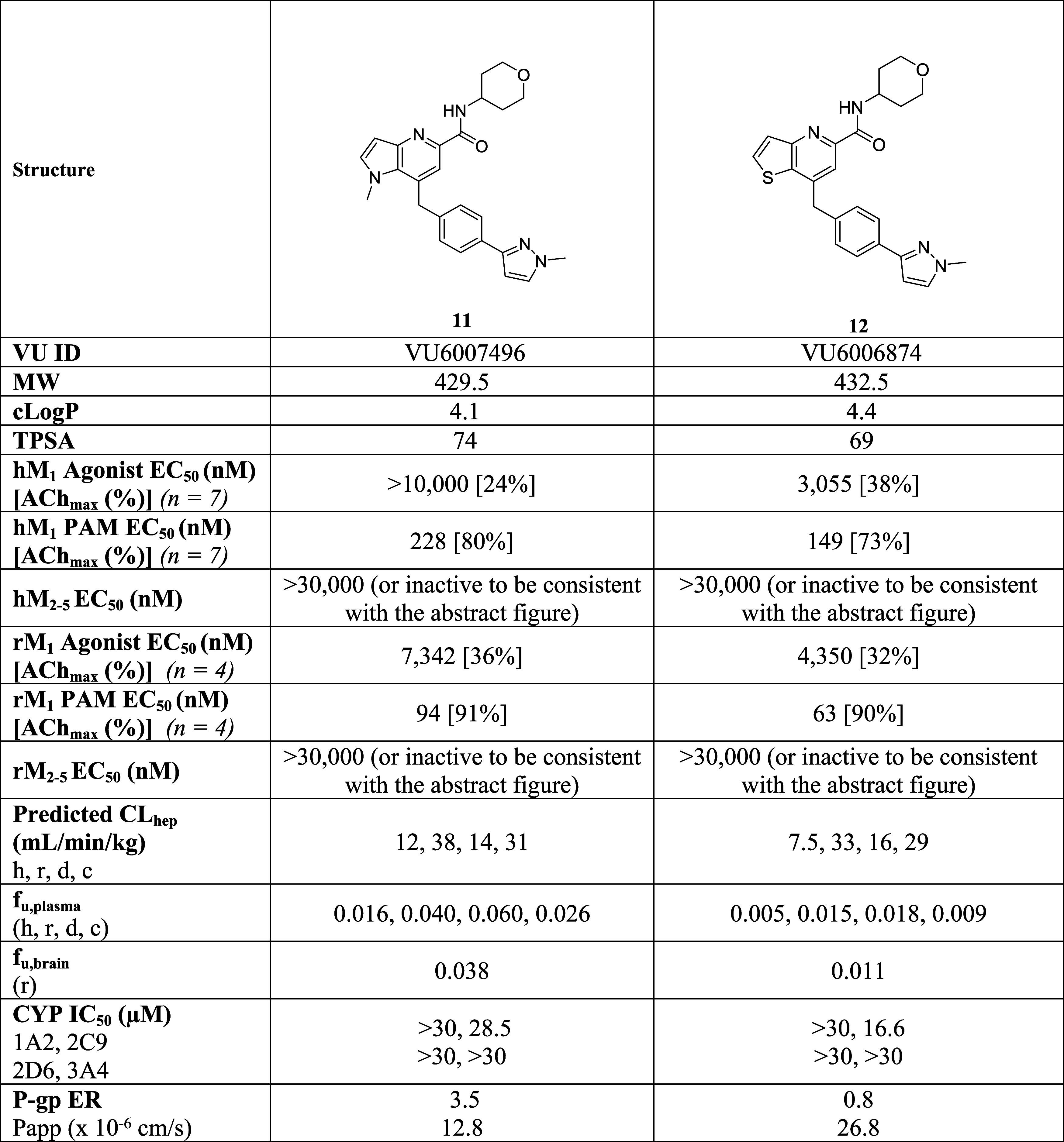
Pharmacology and *In Vitro* DMPK Profiles of **11** and **12**^a^

a *c* Log *P* and TPSA were calculated using ChemDraw Professional 22.2.

On human M_1_, **11** was found to be an M_1_ PAM (EC_50_ = 228 nM, 80% ACh max, pEC_50_ = 6.78 ± 0.11) with minimal M_1_ agonism (EC_50_ > 10 μM, 24% ACh Max), whereas **12** was a more potent M_1_ PAM (EC_50_ = 149 nM, 73% ACh max, pEC_50_ = 6.88 ± 0.08), but displayed moderate M_1_ agonism (EC_50_ = 3.1 μM, 38% ACh Max).^21^ Both **11** and **12** were potent on rat M_1_ with PAM potencies of 94 nM (91% ACh Max) and 63 nM (90% ACh Max), respectively, and both showed moderate rat M_1_ agonism. Importantly, both **11** and **12** were inactive on human and rat M_2–5_ (EC_50_s > 30 μM). PAMs **11** and **12** displayed moderate predicted hepatic clearance across human, rat and dog; however, both PAMs possessed high predicted hepatic clearance in cyno (≥30 mL/min/kg). Both PAMs showed acceptable plasma and brain homogenate binding profiles, but **12** uniformly showed less fraction unbound. Similarly, both **11** and **12** demonstrated acceptable CYP_450_ profiles against CYP3A4, CYP1A2, CYP2C9 and CYP2D6 (IC_50_s > 16 μM). In terms of predicted CNS penetration in human, **11** had an MDCK-MDR1 ER ratio of 3.5 with a *P*_app_ of 12.8, and, based on controls in this assay, was acceptable to move forward, whereas **12** was not a P-gp substrate (ER = 0.8) and had good permeability (*P*_app_ = 26.8). In a Lead Profiling Screen of 68 GPCRs, ion channels and transporters employing radioligand displacement to assess ancillary pharmacology, **11** had no significant results (<50% displacement at 10 μM), whereas **12** had a single significant hit (melatonin MT1, 70% at 10 μM).^21^ In a 4-strain Ames assay, with and without S9, both **11** and **12** were negative. A GSH trapping study in human liver microsomes to test for reactive intermediates showed similar negative results for the two PAMs. Finally, an electrophysiology panel of cardiac ion channels was clean (<17% inhibition at 10 μM) for both PAMs.^21^ Thus, both PAMs possessed acceptable overall *in vitro* profiles to advance into the tier of the development workflow; however, in the team’s opinion, PAM **11** was the leading contender due to higher fraction unbound and less M_1_ agonism.

### *In Vivo* Behavior and DMPK

**Figure 4:**
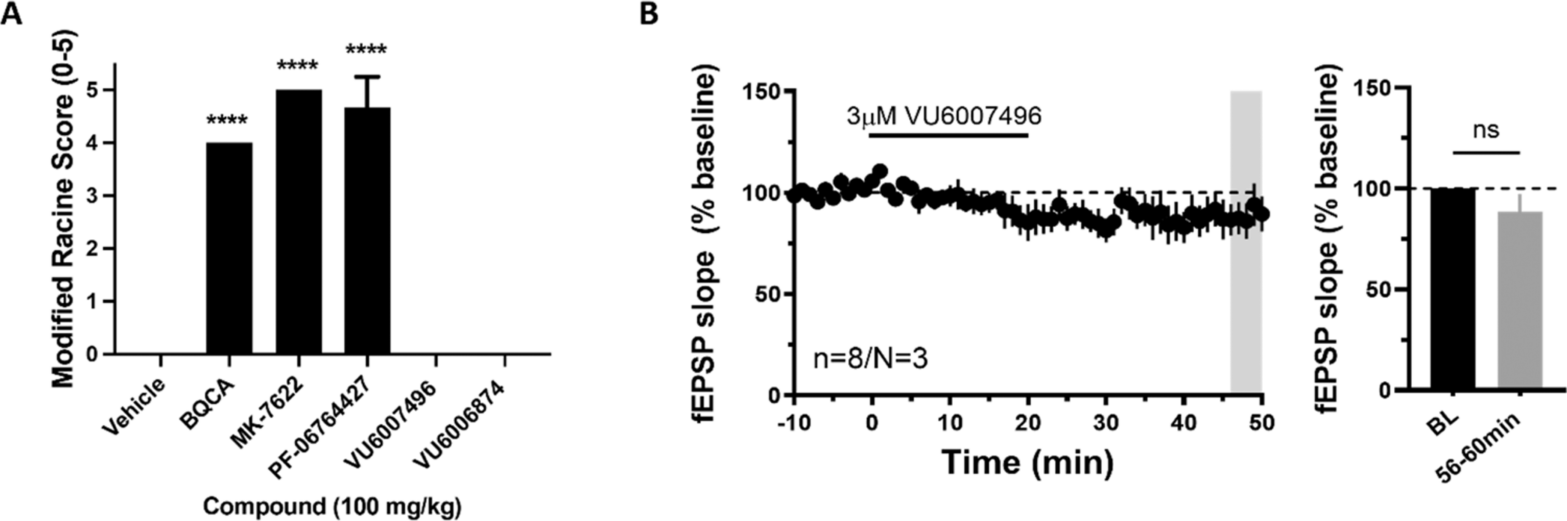
(A) Modified Racine Score test in mice with M_1_ PAMs. Pretreatment with M_1_ PAMs (100 mg/kg, i.p., 10 mL/kg, 180 min) BQCA (**2**), MK-7622 (**3**), PF-0674427 (**4**), resulted in robust behavioral convulsions at 3 h post administration, while VU6007496 (**11**) and VU6006874 (**12**) did not cause any observed adverse effects. *N* = 3/group of male C57Bl/6 mice. ANOVA *p* < 0.0001; ****p < 0.0001 as compared to vehicle control. (B) Time course graph showing that bath application of 3 μM VU6007496 (**11**) for 20 min led to no significant change in fEPSP slope. *N* = 8 brain slices from 3 different male C57Bl/6 mice.

PAMs **11** and **12** were next evaluated in our rat IV plasma: brain level cassette paradigm (0.25 mg/kg compound, 1 mg/kg total dose, 10.1% EtOH:40.4% PEG400:49.5% DMSO) sampled at a set 15 min time point to assess CNS penetration. **11** had a *K*_p_ (the partitioning coefficient between plasma and brain) of 0.42 (plasma, 92.2 ng/mL: brain, 39 ng/g) and a *K*_p,uu_ of 0.36; in contrast, **12** had a *K*_p_ of 1.1 (plasma, 105 ng/mL: brain, 120 ng/g) and a *K*_p,uu_ of 0.7.^20^ These data were in alignment with the human predicted CNS penetration from the MDCK-MDR1 P-gp *in vitro* assay discussed earlier and supported continued advancement. As mice are the most sensitive species to cholinergic mechanisms,^11,13,23,24^ we placed a high-throughput phenotypic seizure liability assay into the work-flow in which potent ago-PAMs, such as **2**–**5**,^8−15^ display robust Racine scale 4/5 behavioral convulsions that develop within minutes of dosing and last for the 3 h duration of the study. Here, a high dose (100 mg/kg intraperitoneal (i.p.)) of either **11** or **12** in mice did not induce seizure liability, akin to **6**–**9** and VU-319/ACP-319, for the 3-h duration of the study (Figure 4A).^21^ For **11** in particular, the 100 mg/kg i.p. study afforded total brain exposure of 1.04 μM, and the mouse M_1_ PAM EC_50_ was determined to be 92 nM (66% ACh max). In parallel, we performed electrophysiology studies in mouse native tissue layer V medial prefrontal cortex (mPFC). While ago-PAMs such as **2**–**5** induce substantial long-term depression that correlates with a lack of robust pro-cognitive efficacy, PAM **11** did not induce significant changes in field excitatory post synaptic potentials (fEPSPs) recorded from layer V and evoked by electrical stimulation in layer II/III at 3 μM concentration (∼30x above the functional mouse EC_50_), and, therefore, maintain activity dependence of PFC function (Figure 4B).^21^ Thus, both compounds cleared the major hurdles of CNS penetration and the liability of M_1_ overstimulation.

We next conducted multispecies IV/PO PK in parallel as a potential discerning data set. PAM **11** possessed an attractive PK profile across rat, dog and nonhuman primate (cynomologus monkey (cyno)) (Table 2). In rat, **11** showed low-to-moderate clearance (26 mL/min/kg) with a 6.1 h half-life and 66% oral bioavailability. The PK of **11** in dog and cyno was characterized by very low clearance (2.4 and 5.9 mL/min/kg, respectively), long half-life in dog (12.8 h) and a short half-life in cyno (1 h), driven by a low volume (0.39). Oral bioavailability for dog was 35% and cyno was 59%. These data were generated prior to any significant formulation of vehicle screens; thus, we were very pleased with the profile of **11**. PAM **12** was similar in disposition, with moderate clearance (33 mL/min/kg) with a 5.3 h half-life and 100% oral bioavailability in rat. Clearance was low in dog (1.3 mL/min/kg) and cyno (2.3 mL/min/kg), and, once again, a long half-life in dog (36.6 h) and moderate in cyno (4.5 h). However, while oral bioavailability was excellent in rat (100%) and cyno (79%), but it was poor in dog (9%).^21^ With a strong desire to employ dog as the nonrodent safety species, the challenge to address the low %F in dog was enough to triage PAM **12** and focus on the advancement of **11** (Table 3).

**Table 2:** Pharmacokinetic Parameters of **11**

parameter	rat (SD)	dog (beagle or mongrel)	NHP (cyno)
dose (mg/kg) iv/po	1/10	1/5	1/5
CL_p_ (mL/min/kg)	26	2.4	5.9
*V*_ss_ (L/kg)	3.2	2.2	0.39
elimination *t*_1/2_ (h)	6.1	12.8	1.0
*F* (%) po	66	35	52
*K*_p_	0.42		
*K*_p,uu_	0.36		

**Table 3:** Pharmacokinetic Parameters of **12**

parameter	rat Sprague–Dawley (SD)	dog (beagle or mongrel)	NHP (cyno)
dose (mg/kg) iv/po	1/10	1/3	1/3
CL_p_ (mL/min/kg)	33	1.3	2.3
*V*_ss_ (L/kg)	8.2	4.0	0.7
elimination *t*_1/2_ (h)	5.3	36.6	4.5
*F* (%) po	100	9	79
*K*_p_	1.1		
*K*_p,uu_	0.7		

**Figure 5:**
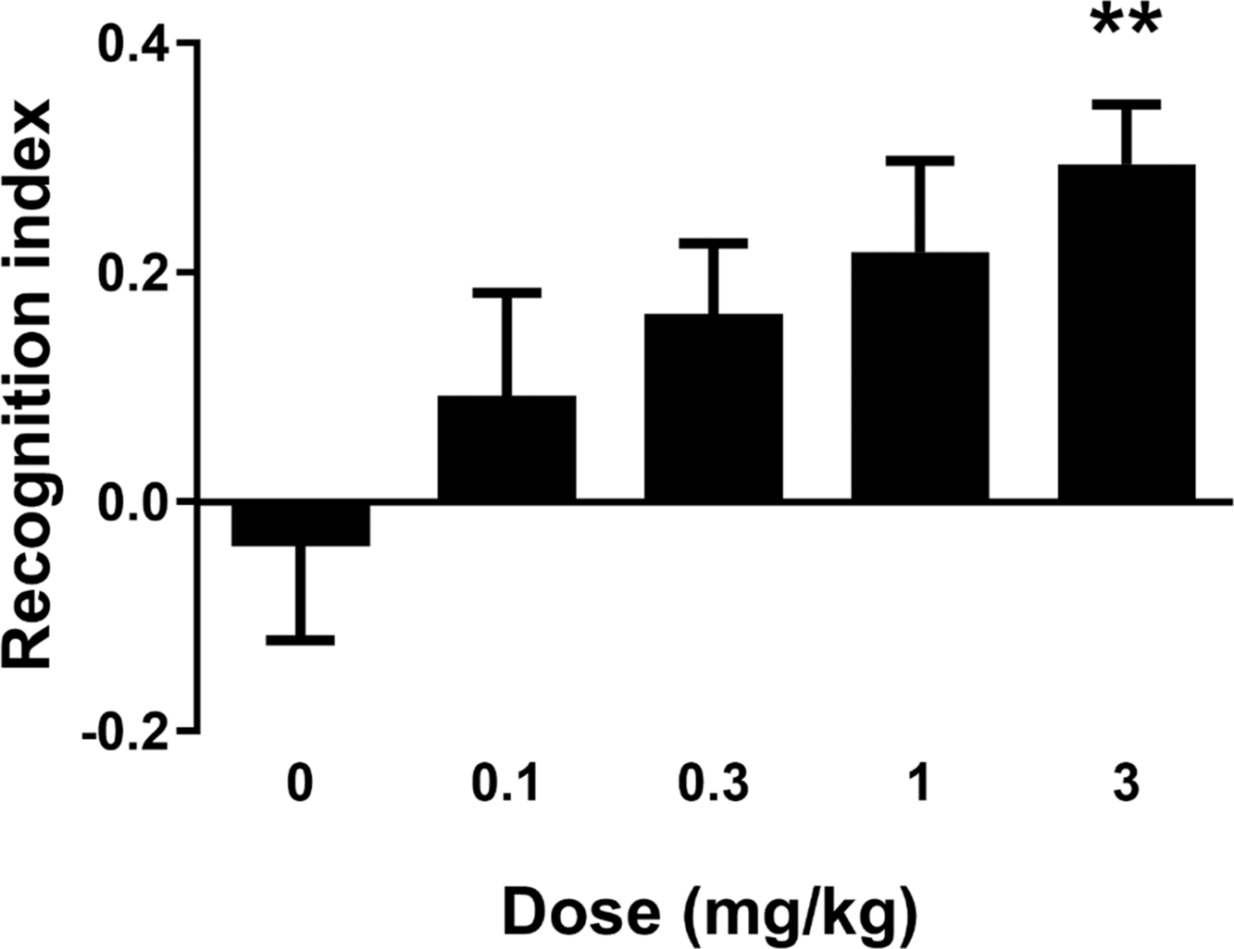
Novel object recognition (NOR) test in rats with VU6007496 (**11**). PAM **11** dose-dependently enhanced recognition memory in rats. Pretreatment with 0.1, 0.3, 1, and 3 mg/kg VU6007496 (p.o, 0.5% natrosol/0.015% Tween 80 in water, 30 min) prior to exposure to identical objects significantly enhanced recognition memory assessed 24 h later. *N* = 15–18/group of male Sprague–Dawley rats. ANOVA *p* = 0.0283; **p < 0.01.

Previously, we have shown that potent M_1_ ago-PAMs with agonist activity in the PFC showed little efficacy in novel object recognition (NOR); in contrast,^15,16^ M_1_ PAMs with no to minimal agonist activity in the PFC displayed robust dose-dependent enhancement of NOR. As illustrated in Figure 5, PAM **11** dose-dependently enhanced recognition memory in rats with a minimum effective dose (MED) of 3 mg/kg p.o., which was in-line with data obtained with previous M_1_ PAMs. Interestingly, a 3 mg/kg p.o. rat satellite PK study demonstrated total brain concentration of 990 nM and an unbound brain concentration of 39.8 nM (rat M_1_ PAM EC_50_ = 94 nM (91% ACh max)). Historically, our PK/PD for M_1_ PAMs in NOR has a strong correlation with total brain as opposed to unbound levels, with MEDs typically 0.25 to 0.4 of the rat EC_50_.^21^ This may be due to higher endogenous cholinergic tone in healthy animals, and the *in vitro* PAM EC_50_s being derived from an arbitrarily selected EC_20_ concentration of ACh as a subthreshold value.

### In-Depth DMPK Profiling

To advance **11** as a candidate and into IND-enabling studies, we next needed to better understand its CYP profile to minimize drug–drug interactions in the clinic. Here, a substrate depletion approach employing recombinant human cytochrome P450s (rCYPs) indicated that rCYP2J2 and rCYP3A4 are responsible for 2.4 and 97.6% of the hepatic CYP-mediated clearance of PAM **11**, respectively. We were pleased to see the contribution of another CYP beyond 3A4 for the metabolism of **11**.^21^ In parallel, we investigated the ability of PAM **11** to induce the expression of CYP_450_s (measuring mRNA) in cryopreserved human hepatocytes from three separate donors. Weak induction liability was noted for CYP1A2 (∼3-fold) and CYP2B6 (∼8-fold), while more concerning induction liability was reported for CYP3A4 (∼21-fold), and would require subsequent evaluation using the assessment of protein expression instead of mRNA.^21^

**Figure 6:**
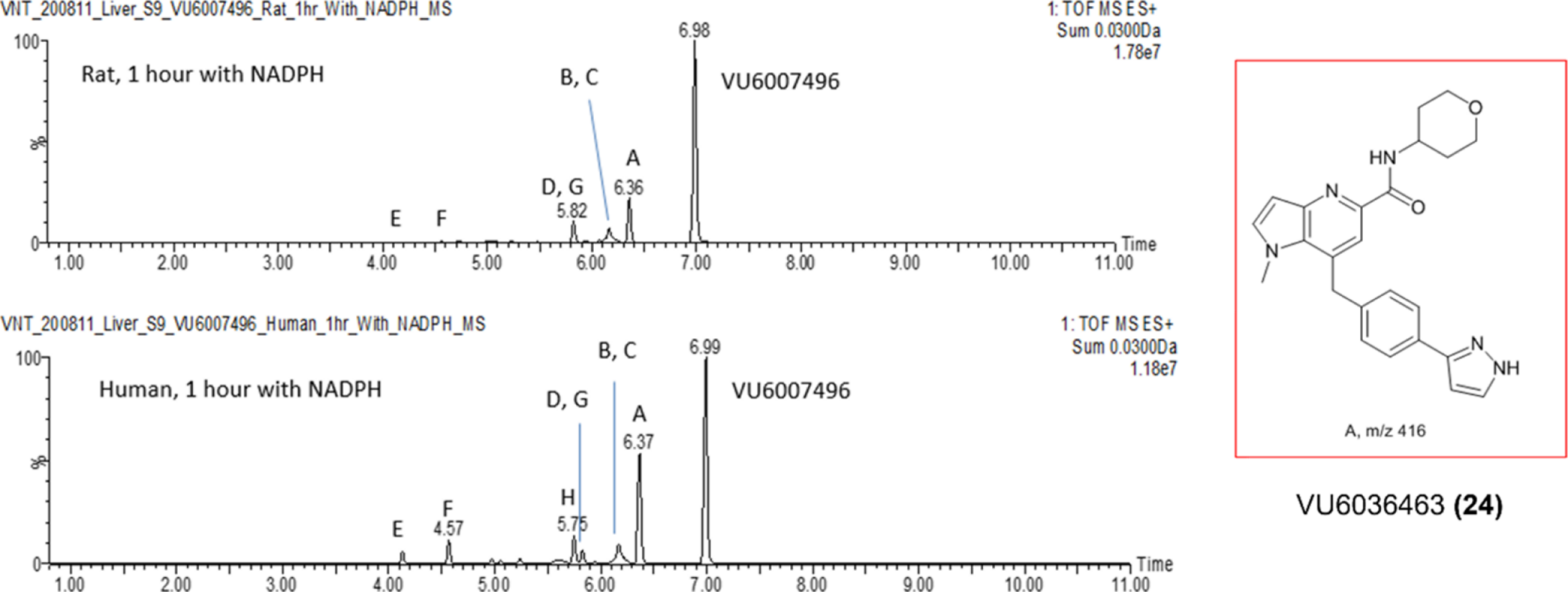
Metabolism of VU6007496 (**11**) in rat and human liver S9, with the major metabolite, VU6036463 (**24**), an *N*-demethylation product, exemplified.

In parallel to these CYP studies, we performed metabolite identification (MET ID) studies in rat and human S9 liver microsomes (Figure 6) and observed comparable coverage of metabolites across human and rat. The PAM **11** proved to be reasonably stable, with the extent of metabolism being 36.8% in rat and 58.1% in human (based on MS peak areas). Eight oxidative metabolites were identified, with the major metabolite in both rat and human being Metabolite A (VU6036463, **24**), the result of oxidative *N*-demethylation of the southern pyrazole (Scheme 4). The majority of other oxidative metabolism occurred on the tetrahydropyran moiety and included ring opening, but there was no amide hydrolysis observed.^21^

**Figure 7:**
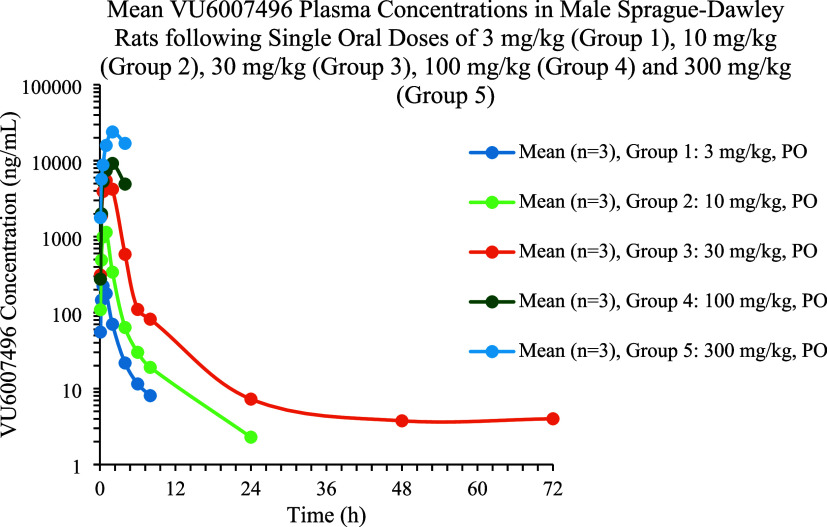
Rat dose escalation study with VU6007496 (**11**).

While awaiting more definitive MET ID studies in multispecies hepatocytes, we elected to perform a rat dose escalation study to assess exposures in a standard, tox-friendly oral vehicle (30% captisol) to lay the foundation for dose range finding/maximum tolerated dose (DRF/MTD) studies. Male Sprague–Dawley rats were dosed with VU6007496 (**11**) at doses of 3, 10, 30, 100, and 300 mg/kg p.o. in 30% captisol. At all doses, PAM **11** was rapidly absorbed with tight *T*_max_ values under 2 h (Figure 7). Linear dose escalation was noted from 3 to 30 mg/kg. However, severe adverse cholinergic events were noted after 4.5 h in the 100 and 300 mg/kg dose groups which was not expected due to the clean mouse phenotypic assay and our long history with other M_1_ PAMs.^20^ Recall, exposures in rat at the NOR MED were 990 nM total and 39.8 nM unbound brain concentrations, respectively. At *C*_max_, the 100 mg/kg dose achieved 9.1 μM total and 298 nM unbound brain concentrations (∼9-fold over the NOR MED exposure), while the 300 mg/kg dose afforded 17.2 μM total and 569 nM unbound brain concentrations (∼17-fold over the NOR MED exposure).^21^ There was a clear, yet unanticipated disconnect.

**Figure 8:**
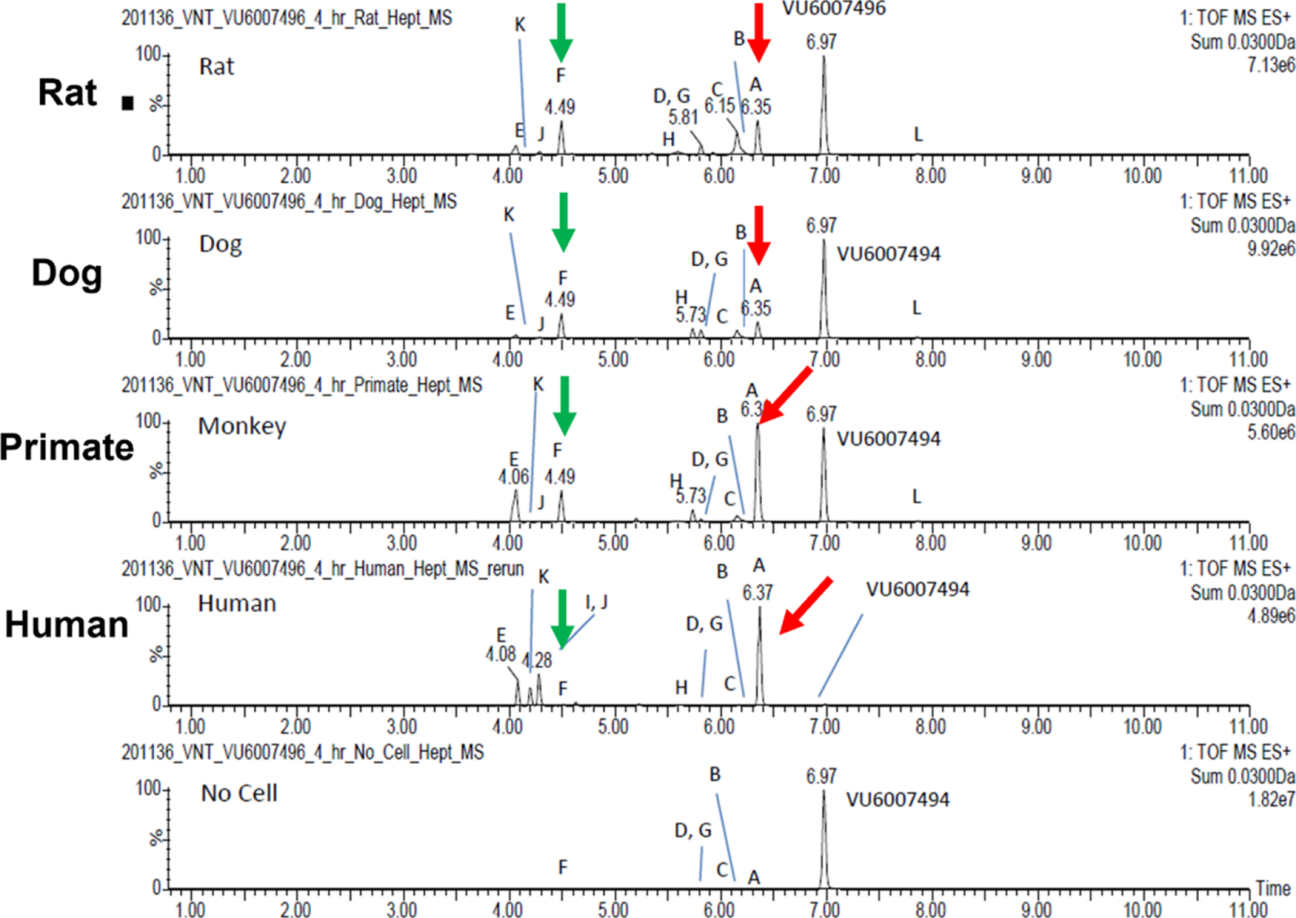
Metabolism of VU6007496 (**11**) in rat, dog, primate and human hepatocytes, with the major metabolite, VU6036463 (**24**), an *N*-demethylation product. Unexpectedly, no parent PAM **11** remained in human after the 4 h incubation.

More concerning were the results generated *via* outsourced multispecies hepatocyte MET ID, which were drastically different than the liver S9MET ID. The extent of metabolism for VU6007496 with rat, dog, monkey and human hepatocytes was 64.7, 51.5, 63.9 and 99.5%, respectively (Figure 8). Twelve metabolites were observed in the 4-h hepatocytes samples, and as opposed to the S9 study, there was no parent **11** remaining in human hepatocytes. In human hepatocytes, PAM **11** was metabolized primarily to Metabolite A (**24**), the result of oxidative *N*-demethylation of the southern pyrazole. The other minor metabolites were oxidation on the tetrahydropyran moiety and included ring opening (Metabolite F, **25**).^21^ With these data, PAM **11** was no longer a backup candidate, but the disconnects and paradigm changing cholinergic toxicity profile warranted further investigation to inform the next backup campaign.

**Figure 9:**
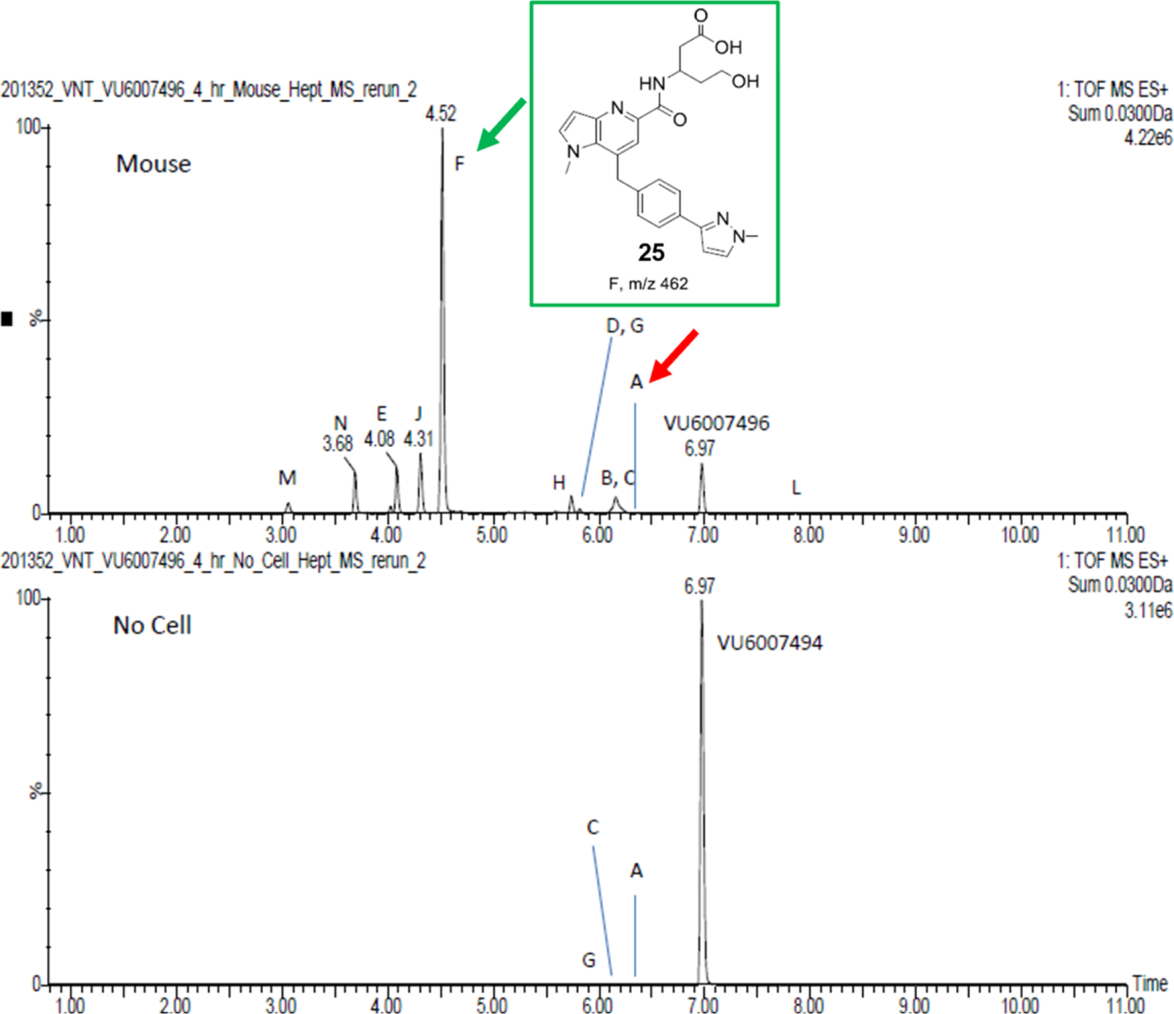
Extensive metabolism (95.4%) of VU6007496 (**11**) in mouse hepatocytes, with metabolite F (**25**) produced in high abundance.

We typically would not examine mouse hepatocyte MET ID, as mouse was not a safety species for the IND-enabling toxicology package, but with the unusual metabolism profile for human, and the lack of predictive cholinergic tox in the mouse phenotypic assay, we felt this was worthy of exploration. As shown in Figure 9, PAM **11** rapidly undergoes extensive metabolism (95.4%) on the tetrahydropyran moiety and almost complete ring opening to the hydroxy acid Metabolite F (**25**).^21^ Clearly, based on the hepatocyte data, there is a tremendous difference in the concentration of parent **11** and metabolite composition, which sheds light on the mouse-rat disconnect for observed cholinergic toxicity in rat *versus* the more cholinergic sensitive mouse.

**Figure 10:**
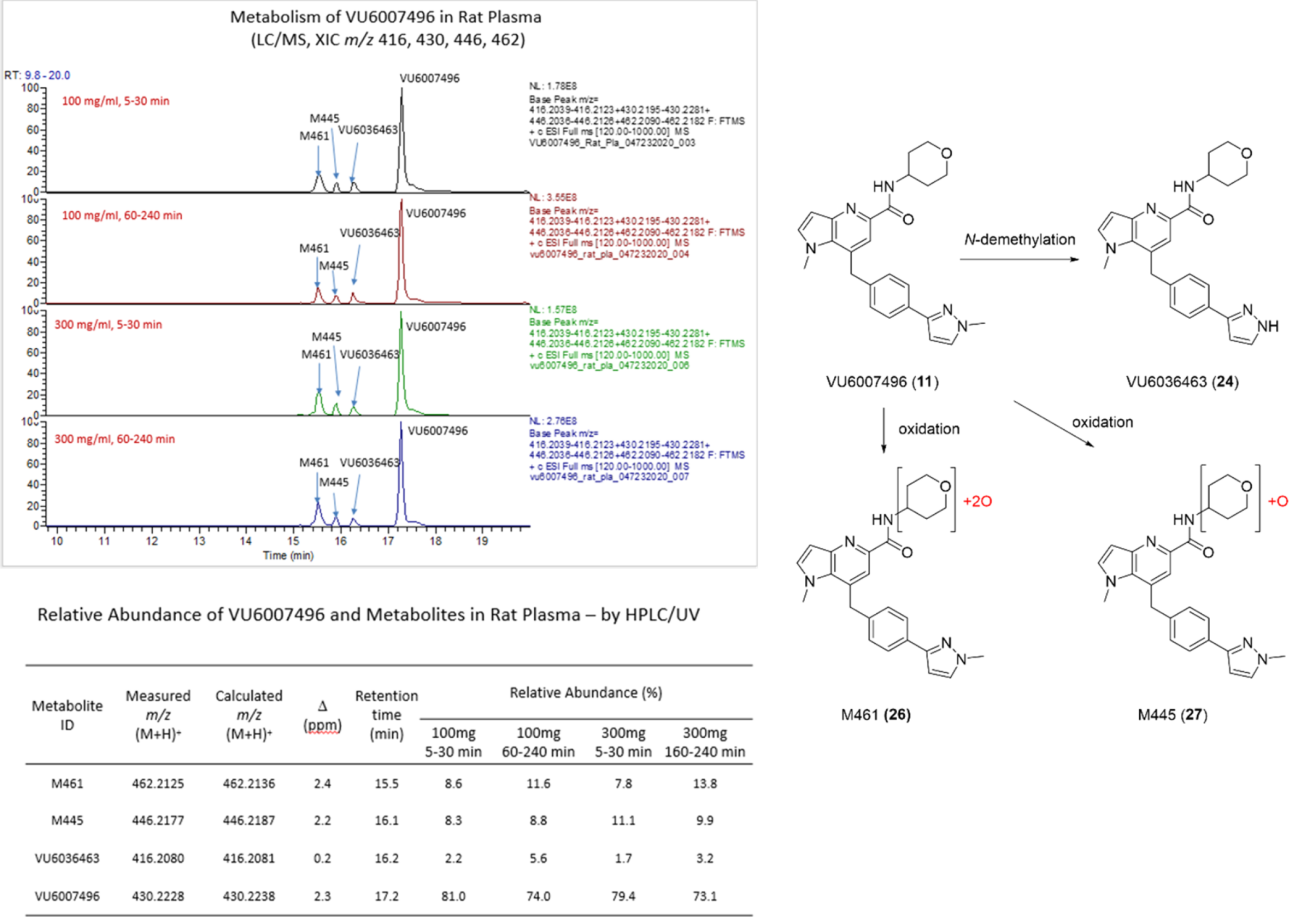
*In vivo* MET ID and metabolic pathways of VU6007496 (**11**) in mouse rat plasma at 100 mg/kg and 300 mg/kg p.o. at 5–30 min and 160–240 min at each dose. Three metabolites are produced *in vivo*: the *N*-demethylated **24**, and two oxidative metabolites, a dioxygenated species M461 (**26**) and a mono-oxygenated species M445 (**27**) on the tetrahydropyranyl moiety.

The team was still puzzled by the severe cholinergic adverse events observed in rats at the 100 and 300 mg/kg arms of the dose escalation study, as this was not consistent with the past 20 years of M_1_ PAM research. Could the *N*-demethylated metabolite A (**24**) be responsible? Due to the disconnect between liver S9 and hepatocytes, we felt it was prudent to examine *in vivo* rat MET ID at doses of 100 and 300 mg/kg to ensure **24** is produced upon *in vivo* oral dosing after 5–30 min and 160–240 min, and to determine if any other putative metabolites are generated at detectable levels (Figure 10). Across both doses and time points, 73–81% of the parent **11** remained. Metabolite **24** was produced *in vivo* at 2.2 to 5.6% relative abundance and lower than from *in vitro* incubations, along with two oxidative metabolites, a dioxygenated species M461 (**26**) and a mono-oxygenated species M445 (**27**) on the tetrahydropyranyl moiety. These were produced in higher relative abundance than **24**, representing 7.7 to 13.8% (**26**) and 8.3 to 11.1% (**27**), respectively.^21^ A species such as **27** would resemble the hydroxy pyranyl congeners known to be potent ago-PAMs and prone to severe cholinergic side effects, and the potential for **24** to elicit similar cholinergic toxicity was unknown. However, it was clear from these data that PAM **11** rapidly generated stable metabolites with strong potential to be “active” metabolites.

### Synthesis and Characterization of Metabolites

Due to the prevalence of metabolite A (VU6036463, **24**) in both *in vitro* preparations and *in vivo*, we first synthesized and characterized **24**. Utilizing intermediate **19** (Scheme 3), a Suzuki coupling with benzyl chloride **28** afforded a mixture of *N*-Boc protected analog **29** and *N*-H analog **29a** in moderate isolated yield. Hydrolysis of the nitriles to the carboxylic acid, followed by a HATU-mediated coupling with tetrahydro-2*H*-pyran-4-amine delivered **24** in 81% yield over the two steps.^21^ In our kinetic assays, **24** was an “active” metabolite, with the profile of an M_1_ ago-PAM. Metabolite **24** was a potent M_1_ PAM on both rat (EC_50_ = 56 nM, 89% ACh max) and human (EC_50_ = 144 nM, 73% ACh max); in fact, more potent than the parent **11**. PAM **24** also displayed M_1_ agonism at both the rat (EC_50_ = 3.1 μM, 48% ACh Max) and human receptors (EC_50_ = 2.1 μM, 36% ACh Max). As with the parent **11**, metabolite **24** was inactive on rat and human M_2–5_. In our tier 1 *in vitro* DMPK panel, **24** displayed an acceptable profile with moderate predicted hepatic clearance (hCL_hep_ = 12.8 mL/min/kg and rCL_hep_ = 44 mL/min/kg), good unbound fraction in human (*f*_u_ = 0.047), rat (*f*_u_ = 0.134) and rat brain homogenate binding (*f*_u_ = 0.025), and an acceptable CYP profile (>30 μM @ 1A2, 2C9, 2D6 and 5.7 μM @ 3A4). To ascertain if **24** could be the source of the adverse cholinergic events in rats, we dosed **11** and **24**, in parallel, at a dose of 100 mg/kg i.p. in 30% captisol and prepared plasma and brain samples at a 3-h time point to determine brain exposure of the M_1_ ago-PAM **24**. In this study, **11** achieved a total plasma concentration of 3.6 μM and a total brain concentration of 0.93 μM (*K*_p_ = 0.26). In contrast, and again—unexpectedly—the metabolite **24** displayed lower exposure in plasma (1.2 μM) and very low (0.08 μM) brain exposure (*K*_p_ = 0.07).^21^ These data suggest that the adverse events in rats was unlikely due to CNS activity of the “active” metabolite **24**. To further confirm this *in vivo* finding, **24** was found to be a P-gp substrate (MDR1-MDCK ER = 31.9, *P*_app_ = 2.0 × 10–6 cm/s) rendering **24** a nonparticipant in the observed toxicity of **11**. At the same time, we explored an *N*-CD_3_ congener of **11** to determine if the kinetic isotope effect would engender metabolic stability to the alkylated pyrazole and avoid the production of **24**, but the metabolism proved to be identical to the *N*-CH_3_.

**Figure 11:**
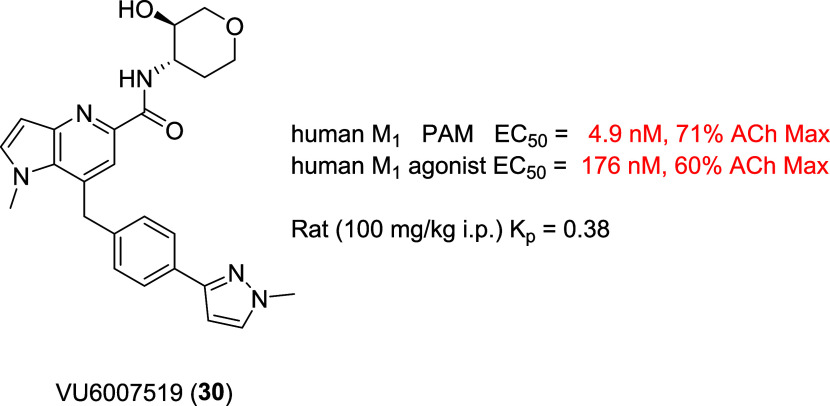
Structure and human M_1_ pharmacology of **30**, an extremely potent M_1_ ago-PAM with CNS penetration in rat.

Based on the known cholinergic adverse events with hydroxy pyranyl amides such as **2**–**5**,^8−12^ and the rat *in vivo* MET ID suggesting, by mass, that M445 (**27**) was a similar species, we prepared analog **30** (VU6007519) following Scheme 2. As shown in Figure 11, this putative metabolite was an extremely potent human M_1_ ago-PAM (PAM EC_50_ = 4.9 nM, 71% ACh Max; agonist EC_50_ = 176 nM, 60%), and this profile would likely give rise to the cholinergic adverse events seen in rats at doses over 100 mg/kg. Importantly, **30** was also brain penetrant in rat at 100 mg/kg i.p. (*K*_p_ = 0.38) and comparable to **11** (*K*_p_ = 0.26). To provide additional evidence that this “active” metabolite was responsible for the overactivation of M_1_ and the observed toxicity, coinjection of **30** with the rat *in vivo* MET ID proved that **30** was not in fact the metabolite 445 (**27**) despite identical masses.^21^ Thus, **27** was possibly another stereo- or regio-isomer of **30**, but quite likely a potent M_1_ ago-PAM. While a valuable and interesting line of investigation, the project team had to refocus on ligands with the potential to advance as backups to our clinical asset, VU319/ACP-319.

## Conclusions

In summary, a lead optimization campaign to identify a suitable backup for the clinical compound, VU319/ACP-319, focused on scaffold-hopping from the pyrrolo[2,3-*b*]pyridine-based M_1_ PAM, VU6007477, to isomeric pyrrolo[3,2-*b*]pyridine and thieno[3,2-*b*]pyridine congeners. From this effort, VU6007496, a pyrrolo[3,2-*b*]pyridine, advanced into late stage profiling, only to be plagued with unanticipated, species-specific metabolism, *in vitro*/*in vivo* disconnects and “active” and potentially toxic metabolites. For this program, *in vitro* liver S9, hepatocyte and *in vivo* MET ID were critical to identify putative “active” metabolites. The unexpected and species-specific mouse metabolism thwarted our phenotypic cholinergic seizure liability *in vivo* screen as a stage gate, and thus prevented further development of VU6007496 as a backup clinical candidate. However, VU6007496 proved to be a highly selective and CNS penetrant M_1_ PAM (with minimal agonism), with excellent multispecies IV/PO PK, CNS penetration, no impact on long-term depression (or cholinergic toxicity) and robust efficacy in novel object recognition (MED = 3 mg/kg p.o.). Cholinergic toxicity was not observed in rats at 30 mg/kg p.o., providing a 10-fold window from the MED, and suggesting that VU6007496 can serve as another valuable *in vivo* rat tool compound, but not mouse, to study selective M_1_ activation *in vivo*.

## Supporting Information Available

Additional experimental details; methods for the synthesis and characterization of all compounds; *in vitro* and *in vivo* DMPK protocols; eurofins lead profiling screen data; synthesis of VU6006874 (**12**) (PDF)

## Abbreviations

PAM: positive allosteric modulator
PBL: plasma/brain level
DMPK: drug metabolism and pharmacokinetics
AE: adverse event
M_1_: muscarinic acetylcholine receptor subtype 1
MED: minimum effective dose
NOR: novel object recognition
