# Supplementary material for: Discovery of VU6007496: Challenges in the Development of an M1 Positive Allosteric Modulator Backup Candidate
Source: ACS Chem Neurosci. 2024 Aug 28;15(18):3421–33. doi: 10.1021/acschemneuro.4c00508 (PMC11413853; doi:10.1021/acschemneuro.4c00508)
Supplement: Supplementary file 1 — cn4c00508_si_001.pdf [file cn4c00508_si_001.pdf]

Supporting Information

Discovery of VU6007496: Challenges in the Development of an M_1 Positive Allosteric Modulator Back-up Candidate

Julie L. Engers,[1,2] Katrina A. Bollinger,[1,2] Rory A. Capstick,[1,2] Madeline F. Long,[1,2] Aaron M. Bender,[1,2] Jonathan W. Dickerson,[1,2] Weimin Peng,[1,2] Christopher C. Presley,[1,2] Hyekyung P. Cho,[1,2] Alice L. Rodriguez,[1,2] Colleen M. Niswender,[1,2,4,5,6] Zixiu Xiang,[1,2] Anna L. Blobaum,[1,2] Olivier Boutaud,[1,2] Jerri M. Rook,[1,2] Darren W. Engers,[1,2] P. Jeffrey Conn,[1,2,4] and Craig W. Lindsley*[1,2,3,4]

Affiliation:
[1]Warren Center for Neuroscience Drug Discovery, Vanderbilt University, Nashville, TN 37232, USA
[2]Department of Pharmacology, Vanderbilt University School of Medicine, Nashville, TN 37232, USA
[3]Department of Chemistry, Vanderbilt University, Nashville TN 37232, USA
[4]Vanderbilt Kennedy Center, Vanderbilt University Medical Center, Nashville, TN 37232, USA
[5]Vanderbilt Brain Institute, Vanderbilt University, Nashville, TN 37232, USA
[6]Vanderbilt Institute of Chemical Biology, Vanderbilt University, Nashville, TN 37232, USA

*To whom correspondence should be addressed at craig.lindsley@vanderbilt.edu

TABLE OF CONTENTS

Eurofins Lead Profiling Screen and Cardiac Ion Channel Screen....S2
Procedures for Biological Experiments....S5
General Methods....S12
Synthetic Procedures....S13
^{1}H NMR Spectra, ^{13}C NMR Spectra, and LC-MS Traces....S24
References....S47

Table S1. Eurofins Lead Profiling Screen Data

This is a radioligand binding panel of 67 targets including GPCRs, ion channels, transporters and nuclear hormones. Biochemical assay results are presented as the percent inhibition of specific binding at a 10 µM concentration of **VU6007496** (Compound **11**).

**Target/Protein**	**Species**	**% Inhibition at 10 µM**
Adenosine A_1	Human	10
Adenosine A_{2A}	Human	-11
Adenosine A_3	Human	38
Adrenergic α_{1A}	Human	-1
Adrenergic α_{1B}	Human	14
Adrenergic α_{1D}	Human	-6
Adrenergic α_{2A}	Human	-7
Adrenergic β_1	Human	-14
Adrenergic β_2	Human	7
Androgen (Testosterone)	Human	-24
Bradykinin B_1	Human	21
Bradykinin B_2	Human	-3
Calcium Channel L-Type, Benzothiazepine	Rat	12
Calcium Channel L-Type, Dihydropyridine	Rat	-8
Calcium Channel N-Type	Rat	8
Cannabinoid CB_1	Human	5
Dopamine D_1	Human	5
Dopamine D_{2S}	Human	5
Dopamine D_3	Human	8
Dopamine $D_{4.4}$	Human	1
Endothelin ET_A	Human	3
Endothelin ET_B	Human	-5
Epidermal Growth Factor (EGF)	Human	5
Estrogen ERα	Human	5
$GABA_A$, Flunitrazepam, Central	Rat	-3
$GABA_A$, Muscimol, Central	Rat	-1
$GABA_{B1A}$	Human	4
Glucocorticoid	Human	5
Glutamate, Kainate	Rat	-19
Glutamate, NMDA, Agonism	Rat	0
Glutamate, NMDA, Glycine	Rat	0
Glutamate, NMDA, Phencyclidine	Rat	7
Histamine H_1	Human	5
Histamine H_2	Human	28
Histamine H_3	Human	-12
Imidazoline I_2, Central	Rat	-9

Interleukin IL-1 R1	Human	10
Leukotriene, Cysteinyl $CysLT_1$	Human	3
Melatonin MT_1	Human	7
Muscarinic M_1	Human	-8
Muscarinic M_2	Human	0
Muscarinic M_3	Human	-4
Neuropeptide Y Y_1	Human	-10
Neuropeptide Y Y_2	Human	31
Nicotinic Acetylcholine α3β4	Human	6
Nicotinic Acetylcholine α1, Bungarotoxin	Human	-3
Opiate δ_1 (OP1, DOP)	Human	5
Opiate κ (OP2, KOP)	Human	32
Opiate μ (OP3, MOP)	Human	-4
Phorbol Ester	Mouse	-1
Platelet Activating Factor (PAF)	Human	-13
Potassium Channel [K_{ATP}]	Hamster	11
Potassium Channel hERG	Human	11
Prostanoid EP_4	Human	16
Purinergic P2X	Rat	17
Purinergic P2Y, Non-Selective	Rat	-9
Rolipram	Rat	10
Serotonin (5-HT_{1A})	Human	7
Serotonin (5-HT_{2B})	Human	7
Serotonin (5-HT_3)	Human	-3
Sigma σ_1	Human	-8
Sodium Channel, Site 2	Rat	19
Tachykinin NK_1	Human	8
Thyroid Hormone	Rat	2
Transporter, Dopamine (DAT)	Human	13
Transporter, GABA	Rat	-17
Transporter, Norepinephrine (NET)	Human	12
Transporter, Serotonin (SERT)	Human	-7

Table S2. Effects of VU6007496 on Ion Channel Current (Charles River Study 200609.WVN)

Ion Channel	Conc (μM)	Mean % Inhibition	Standard Deviation	Standard Error	n	Individual Data Points (% Inhibition)
hCav1.2	10	12.8	8.2	4.1	4	3.5
						9.0
						17.0
						21.9
hCav3.2	10	4.2	4.4	2.2	4	-0.1
						3.2
						3.3
						10.3
hHCN2	10	4.9	0.9	0.5	3	5.9
						4.5
						4.1
hERG	10	2.6	2.4	1.4	3	4.7
						3.2
						-0.1
hKv1.3	10	16.5	8.9	4.5	4	19.0
						7.4
						27.8
						11.7
hKv1.5	10	8.7	3.2	1.9	3	6.0
						7.9
						12.3
hKvLQT1/minK	10	13.5	8.5	4.2	4	16.1
						0.9
						19.2
						17.7
hNav1.5 (Tonic)	10	3.9	5.1	2.6	4	-0.7
						9.9
						6.3
						-0.1
hNav1.5 (Phasic)	10	11.4	5.0	2.5	4	9.7
						16.2
						14.5
						5.0

Procedures for Biological Experiments

Calcium mobilization assays:

All functional cell-based assays were performed in stable Chinese Hamster Ovary (CHO) cell lines constitutively expressing human M_5 or human M_1 receptors. For full muscarinic selectivity, CHO cells expressing human M_3, human M_2 plus G_{qi5} or human M_4 plus G_{qi5} were used. Cells were plated at 15,000 cells per 20 μL per well in black 384-well, TC-treated, clear-bottomed plates (Greiner) in Ham's F12 medium supplemented with 10% FBS and 20 mM HEPES. Cells were incubated overnight at 37 °C under 5% CO_2. The following day, the medium was removed and replaced with 1.2 μM Fluo-4 AM (Invitrogen) in assay buffer (Hank's Balanced Salt Solution supplemented with 20 mM HEPES and 2.5 mM Probenecid, pH 7.4) and the cells were incubated for 50 minutes at 37 °C under 5% CO_2. Dye was then removed and replaced with 20 μL of fresh assay buffer. Test compounds at a 10 mM concentration in DMSO were serially diluted in DMSO (either 1:3 or 1:5 dilution) to create a 10-point concentration series. The DMSO solutions were then diluted in assay buffer resulting in compound solutions at 2-times the final assay concentration with the highest assay concentration of 30 μM. The compound plate, cell plate, and plates containing EC_{20} and EC_{80} acetylcholine concentrations were placed in a Hamamatsu FDSS 6000 or 7000 kinetic imaging plate reader equipped to measure Ex_{480}/Em_{540} fluorescence. Data were collected at 1 frame per second. After 2 seconds of collecting baseline fluorescence, 20 μL of the compound solutions were added to the cell plate. This was followed by the addition of an EC_{20} concentration of acetylcholine at 142 seconds. At 267 seconds, an EC_{80} concentration of acetylcholine was added along with a maximally effective acetylcholine concentration in wells not containing a compound to allow data normalization. The fluorescence signal was collected for a total of 300 seconds. Compound concentration response curves (CRCs) were collected in triplicate across three separate plates. Data were imported and analyzed in Dotmatics Informatics software. by normalizing all data in the individual kinetic traces to the initial fluorescence read. The magnitude of each agonist addition was then determined and normalized to the average maximum response. This percent maximum response was plotted against log[compound] and fit to a four parameter logistical equation to determine log(IC_{50}). The IC_{50} determined using the EC_{80} of acetylcholine is the value reported. Compound CRC curves that did not plateau below 10% ACh_{max} were assigned a low potency value of >10 μM.

Drug Metabolism Methods:

In vitro

Plasma protein binding and Brain homogenate binding: Determination of fraction unbound (f_u) in plasma was conducted in vitro via equilibrium dialysis using HTDialysis (HTD) membrane plates. The top half of the plate was filled with 100 uL of Dubelco's Phosphate Buffered Saline, pH 7.4 (DPBS). Compounds were diluted into plasma from each species (5 µM final concentration), which was aliquoted in triplicate to the 'bottom half' of the prepared HTD plate wells. The HTD plate was sealed and incubated for 6 hours at 37 ˚C. Following incubation, each well (both top and bottom halves) were transferred (20 µL) to the corresponding wells of a 96-shallow-well (V-bottom) plate. The daughter plates were then matrix-matched (DPBS side wells received equal volume of plasma, and plasma side wells received equal volume of DPBS), and extraction solution (120 µL; acetonitrile containing 50 nM carbamazepine as IS) was added to all wells of both daughter plates to precipitate protein and extract test article. The plates were then sealed and centrifuged (3500 rcf) for 10 minutes at ambient temperature. Supernatant (60 µL) from each well of the daughter plates was then transferred to the corresponding wells of new daughter plates (96-shallow-well, V bottom) containing water (Milli-Q, 60 µL/well), and the plates were sealed in preparation for LC-MS/MS analysis (see LC-MS/MS analysis method below).

The unbound fraction (f_u) was calculated following the equation below, and mean values for each species were calculated from 3 replicates.

A similar approach was used to determine the degree of brain homogenate binding, which employed the same methodology and procedure with the following modifications: 1) a final compound concentration of 1 µM was used, 2) naïve rat brains were homogenized in DPBS (1:3 composition of brain: DPBS, w/w) using a Mini-Bead Beater™ machine in order to obtain brain homogenate, which was then treated in the same manner as the plasma samples in the previously described plasma protein binding assay. Fraction unbound for both plasma and brain samples was determined using Equation 4.

$$f_u = \frac{Conc_{buffer}}{Conc_{plasma}}$$

Equation 4 Determination of fraction unbound in plasma.

The diluted fraction unbound (f_u2) in brain was calculated in the same manner by using brain homogenate rather than plasma. Undiluted fraction unbound for the brain was calculated using Equation 5

$$f_u = \frac{1/4}{\left\{\left(\frac{1}{fu2}\right) - 1\right\} + 1/4}$$

Equation 5 Determination of fraction unbound in brain. F_u2 represents the diluted fraction unbound.

Intrinsic clearance: Human or rat hepatic microsomes (0.5 mg/mL) and 1 µM test compound were incubated in 100 mM potassium phosphate pH 7.4 buffer with 3 mM $MgCl_2$ at 37 °C with constant shaking. After a 5 min preincubation, the reaction was initiated by the addition of NADPH (1 mM). At selected time intervals (0, 3, 7, 15, 25, and 45 min), aliquots were taken and subsequently placed into a 96-well plate containing cold acetonitrile with internal standard (50 ng/mL carbamazepine). Plates were then centrifuged at 3000 rcf (4 °C) for 10 min, and the supernatant was transferred to a separate 96-well plate and diluted 1:1 with water for LC/MS/MS analysis. The *in vitro* half-life ($_{1/2}$, min, Eq. 1), intrinsic clearance (CL_{int}, mL/min/kg, Eq. 2), and subsequent predicted hepatic clearance (CL_{hep}, mL/min/kg, Eq. 3) was determined employing the following equations:

(1) $T_{1/2} = \frac{Ln(2)}{K}$

where k represents the slope from linear regression analysis of the natural log percent remaining of a test compound as a function of incubation time

(2) $CL_{int} = \frac{0.693}{in\ vitro T_{1/2}}\ x\ \frac{mL\ incubation}{mg\ microsomes}\ x\ \frac{45\ mg\ microsomes}{gram\ liver}\ x\ \frac{20^{a}\ gram\ liver}{kg\ body\ wt}$

[a]scale-up factors: of 20 (human) or 45 (rat)

(3) $CL_{hep} = \frac{Q_h \cdot CL_{int}}{Q_h + CL_{int}}$

where Q_h (hepatic blood flow, mL/min/kg) is 21 (human) or 70 (rat).

LC/MS/MS Bioanalysis of Samples from Plasma Protein Binding and Intrinsic Clearance Assays:

Samples were analyzed on a Thermo Electron TSQ Quantum Ultra triple quad mass spectrometer (San Jose, CA) via electrospray ionization (ESI) with two Themo Electron Accella pumps (San Jose, CA), and a Leap Technologies CTC PAL autosampler (Carrboro, NC). Analytes were separated by gradient elution on a dual column system with two Thermo Hypersil Gold (2.1 x 30 mm, 1.9 µm) columns (San Jose, CA) thermostated at 40 °C. HPLC mobile phase A was 0.1% formic acid in water and mobile phase B was 0.1% formic acid in acetonitrile. The gradient started at 10% B after a 0.2 min hold and was linearly increased to 95% B over 0.8 min; hold at 95% B for 0.2 min; returned to 10% B in 0.1 min. The total run time was 1.3 min and the HPLC flow rate was 0.8 mL/min. While pump 1 ran the gradient method, pump 2 equilibrated the alternate column isocratically at 10% B. Compound optimization, data collection, and processing was performed using Thermo Electron's QuickQuan software (v2.3) and Xcalibur (v2.0.7 SP1).

***In vivo* DMPK experimental:**

Determination of brain to plasma ratio:

Animal care and use

All animal study procedures were approved by the Institutional Animal Care and Use Committee and were conducted in accordance with the National Institutes of Health regulations of animal care covered in Principles of Laboratory Animal Care (National Institutes of Health). All rats were fasted overnight prior to testing.

In-life phase

For determination of the brain over plasma ratio (K_p), compounds were formulated in 8% ethanol, 32% PEG400 and 60% DMSO (*v/v/v*) and administered as a single 0.2 mg/kg IV dose (1 mL/kg) to male, Sprague Dawley rats (n = 1) via injection into a surgically-implanted jugular vein catheter. At 15 min post dosing, blood sample was collected into chilled, K_2EDTA anticoagulant-fortified tube and immediately placed on wet ice. The blood sample was then centrifuged (1700 rcf, 5 minutes, 4 ˚C) to obtain plasma sample. At the same post-administration time point, whole

brain sample was obtained by rapid dissection, rinsed with PBS, and immediately frozen in individual tissue collection box (dry ice). All brain and plasma samples were stored at -80 ˚C until analysis by LC-MS/MS.

Sample Analysis: Concentrations in plasma and brain homogenates were quantified by liquid chromatography tandem mass spectrometry (LC-MS/MS). Whole brains were homogenized in 3 mL of 70:30 IPA:water in a mini bead beater for 3 min, and centrifuged at 3,500 g for 5 min. 5 uL of the supernatant was diluted in 15 uL of blank plasma for quantification of the analytes. Plasma samples were centrifuged at 3,500 g for 5 min. A standard curve was generated by diluting the analytes DMSO stocks with blank plasma to obtain a final concentration of 10,000 ng/ml followed by a serial dilution down to 0.5 ng/ml. Quality controls were generated by a serial dilution of the 5,000 ng/ml standard curve solution in blank plasma to obtain 3 concentrations of 500, 50, and 5 ng/ml. 20 uL of brain diluted in plasma, plasma, blank plasma, standard curve and QC samples were loaded in a V-bottom 96-well plate. 120 uL of acetonitrile containing 0.05 uM carbamazepine (internal standard) was added to each well and the plate was centrifuged at 3,500 g for 5 min. 60 uL of the supernatant of each well (protein free) was transferred to a new 96-well plate containing 60 uL of water. The plates were sealed for analysis by LC-MS/MS.

Plasma and brain tissue samples originating from *in vivo* studies were analyzed by electrospray ionization using an AB Sciex Q-TRAP 5500 (Foster City, CA) that was coupled to a Shimadzu LC-20AD pump (Columbia, MD) and a Leap Technologies CTC PAL auto-sampler (Carrboro, NC). Analytes were separated by gradient elution using a C18 column (3 x 50 mm, 3 mm; Fortis Technologies Ltd, Cheshire, UK) that was thermostated at 40 °C. HPLC mobile phase A was 0.1% formic acid in water (pH unadjusted); mobile phase B was 0.1% formic acid in acetonitrile (pH unadjusted). A 10% B gradient was held for 0.2 min and was linearly increased to 90% B over 0.8 min, with an isocratic hold for 0.5 min, before transitioning to 10% B over 0.05 min. The column was re-equilibrated (1 min) before the next sample injection. The total run time was 2.55 min, and the HPLC flow rate was 0.5 ml/min. The source temperature was set at 500 °C, and mass spectral analyses were performed using a Turbo-Ion spray source in positive ionization mode (5.0-kV spray voltage) and using multiple-reaction monitoring of transitions specific for the analytes. All data were analyzed using AB Sciex Analyst 1.5.1 software.

Brain plasma concentration ratio (K_p) was calculated by dividing brain concentration by plasma concentration for each animal. Unbound brain to unbound plasma concentration ratio ($K_{p,uu}$) is calculated using the following formula: $K_{p,uu}$ = (Brain ng/g x brain fu)/(plasma ng/ml x plasma fu).

Pharmacokinetic profiles in rats following oral single escalating doses

Single escalating oral dosing in Sprague-Dawley rats was performed at Frontage Laboratories according to their non-GLP Standard Operating Procedure and IACUC protocols. In short, compounds were formulated in 10% Tween 80 in water and dosed at 10 mg/kg. At different times, arterial blood was collected from a femoral artery catheter, and compound concentration was determined in plasma by LC-MS/MS following their non-GLP protocol. PK parameters were determined using Phoenix WinNonlin software (version 6.3).

In-vitro determination of blood-brain barrier penetration potential

Blood-brain barrier penetration was determined using MDR1-MDCK cell monolayers by Absorption Systems, following their protocol. In short, compounds were incubated at 5 mM final concentration on one side of the cell monolayer for 2 hours. Compounds concentration on either side of the monolayer was determined by LC-MS/MS and apparent permeability and efflux ratio were determined as described in Wang, Q. et al.[1]

Behavioral Manifestations of Seizure Activity

To evaluate induction of behavioral manifestation of seizure activity, C57Bl/6 mice received administration of vehicle or 100 mg/kg M_1 PAM. Compounds were formulated in 30% Captisol (11) or 10% Tween 80 (2-4 and 12) (pH 7.0) at a concentration of 10 mg/mL and injected i.p. (n = 3). Animals were monitored continuously and scored for behavioral manifestations of seizure activity at 5, 10, 15, and 30 min, and 1 and 3 h. Behavioral manifestations of seizures were scored using a modified Racine scoring system.[2] Briefly, a score of 0 represents no behavior alterations; score 1, immobility, mouth and facial movements, or facial clonus; score 2, head nodding, tail extension; score 3, forelimb clonus, repetitive movements; score 4, rearing and tonic clonic seizure; and score 5, continuous rearing and falling, severe generalized tonic clonic seizure.

Novel Object Recognition Task

Rats were habituated for 10 min for 2 consecutive days in an empty novel object recognition (NOR) arena consisting of dark-colored plexiglass box (40 × 64 × 33 cm^3). On day 3, rats were administered vehicle (0.5% natrasol/0.015% Tween80) or M_1 PAM (0.1-3 mg/kg, p.o.., 10 mL/kg, n = 15-18) and returned to their home cage for 30 min. Rats were then placed in the NOR arena containing two identical objects for 10 min. Following the exposure period, rats were placed back into their home cages for 24 h. The rats were then returned to the arena in which one of the previously exposed (familiar) objects was replaced by a novel object and were video recorded for 5 min while they explored the two objects. Time spent exploring each object was scored by an observer blinded to the experimental conditions and the recognition index was calculated as [(time spent exploring novel object) – (time spent exploring familiar object)]/total time exploring objects.

Supplemental field electrophysiology methods

Recording pipettes were constructed from thin-walled borosilicate capillary glass tubing (I.D.=1.17 mm, O.D. 1.50 mm; Warner Instruments, Hamden, CT), pulled with a horizontal pipette puller (P-97 Sutter Instrument Co., Novata, CA) to a resistance of 1-3MΩ when filled with ACSF. Layer II/III was visualized using an Olympus BX50WI upright microscope (Olympus, Lake Success, NY) microscope according to landmarks illustrated in the Allen mouse brain atlas (Lein et al, 2007) and the recording electrode was laterally placed approximately 200µM away from layer 2/3 into layer V so that the recording and stimulating electrodes were parallel to each other. Inputoutput curves were generated to determine the stimulus intensity that produced approximately 70% of the maximum fEPSP slope before each experiment, which was then used as the baseline stimulation. Similarly, the recording electrode for ofEPSP was placed in layer V and an input-output curve was generated to produce approximately 70% of the maximal ofEPSP slope. Data were digitized using a Multiclamp 700B, using a sampling rate of 20,000kHz and were filtered at 0.5kHz, with a Digidata 1322A, pClamp 9.2 and Clampex 10.6.2 software (Molecular Devices) running on a Dell PC (Round Rock, TX). Offline data analysis to calculate fEPSP slope or ofEPSP slope was performed using Clampfit 10.2 (Molecular Devices).

General Methods

All NMR spectra were recorded on a 400 MHz AMX Bruker NMR spectrometer. ^{1}H and ^{13}C chemical shifts are reported in δ values in ppm downfield with the deuterated solvent as the internal standard. Data are reported as follows: chemical shift, multiplicity (s = singlet, d = doublet, t = triplet, q = quartet, b = broad, m = multiplet), integration, coupling constant (Hz). Low resolution mass spectra were obtained on an Agilent 6120/6150 or Waters QDa (Performance) SQ MS with ESI source. *Method A (Agilent 6120/6150):* MS parameters were as follows: fragmentor: 70, capillary voltage: 3000 V, nebulizer pressure: 30 psig, drying gas flow: 13 L/min, drying gas temperature: 350 ºC. Samples were introduced via an Agilent 1290 UHPLC comprised of a G4220A binary pump, G4226A ALS, G1316C TCC, and G4212A DAD with ULD flow cell. UV absorption was generally observed at 215 nm and 254 nm with a 4 nm bandwidth. Column: Waters Acquity BEH C18, 1.0 x 50 mm, 1.7 um. Gradient conditions: 5% to 95% CH_3CN in H_2O (0.1% TFA) over 1.4 min, hold at 95% CH_3CN for 0.1 min, 0.5 mL/min, 55 ºC. *Method B (Agilent 6120/6150):* MS parameters were as follows: fragmentor: 100, capillary voltage: 3000 V, nebulizer pressure: 40 psig, drying gas flow: 11 L/min, drying gas temperature: 350 ºC. Samples were introduced via an Agilent 1200 HPLC comprised of a degasser, G1312A binary pump, G1367B HP-ALS, G1316A TCC, G1315D DAD, and a Varian 380 ELSD (if applicable). UV absorption was generally observed at 215 nm and 254 nm with a 4 nm bandwidth. Column: Thermo Accucore C18, 2.1 x 30 mm, 2.6 um. Gradient conditions: 7% to 95% CH_3CN in H_2O (0.1% TFA) over 1.6 min, hold at 95% CH_3CN for 0.35 min, 1.5 mL/min, 45 ºC. *Method C (Waters QDa (Performance) SQ MS)*: MS parameters were as follows: cone voltage: 15 V, capillary voltage: 0.8 kV, probe temperature: 600° C. Samples were introduced via an Acquity I-Class PLUS UPLC comprised of a BSM, FL-SM, CH-A, and PDA. UV absorption was generally observed at 215 nm and 254 nm; 4 nm bandwidth. Column: Phenomenex EVO C18, 1.0 x 50 mm, 1.7 um. Column temperature: 55° C. Flow rate: 0.4 mL/min. Default gradient: 5% to 95% CH_3CN (0.05% TFA) in H_2O (0.05% TFA) over 1.4 min (curve 6), hold at 95% CH_3CN for 0.1 min. "Polar" (2% to 70% CH_3CN (0.05% TFA) in H_2O (0.05% TFA) over 0.8 min (curve 6), transition to 95% CH_3CN over 0.1 min (curve 6), hold at 95% CH_3CN for 0.6 min.) and "Non-Polar" (40% to 95% CH_3CN (0.05% TFA) in H_2O (0.05% TFA) over 1.4 min (curve 6), hold at 95% CH_3CN for 0.1 min.) gradients were also available. *Method D (Waters QDa (Performance) SQ MS):* MS parameters were as follows: cone voltage: 15 V, capillary voltage: 0.8 kV, probe temperature: 600° C.

Samples were introduced via an Acquity I-Class PLUS UPLC comprised of a BSM, FL-SM, CH-A, and PDA. UV absorption was generally observed at 215 nm and 254 nm with a 4 nm bandwidth. Column: Phenomenex EVO C18, 1.0 x 50 mm, 1.7 um. Column temperature: 55° C. Flow rate: 0.4 mL/min. Default gradient: 5% to 95% CH_3CN in H_2O (5 mM NH_4HCO_3) over 1.4 min (curve 6), hold at 95% CH_3CN for 0.1 min. "Polar" (2% to 70% CH_3CN in H_2O (5 mM NH_4HCO_3) over 0.8 min (curve 6), transition to 95% CH_3CN over 0.1 min (curve 6), hold at 95% CH_3CN for 0.6 min.) and "Non-Polar" (40% to 95% CH_3CN in H_2O (5 mM NH_4HCO_3) over 1.4 min (curve 6), hold at 95% CH_3CN for 0.1 min.) gradients were also available. High resolution mass spectra were obtained on an Agilent 6540 UHD Q-TOF with ESI source. MS parameters were as follows: fragmentor: 150, capillary voltage: 3500 V, nebulizer pressure: 60 psig, drying gas flow: 13 L/min, drying gas temperature: 275 ºC. Samples were introduced via an Agilent 1200 UHPLC comprised of a G4220A binary pump, G4226A 3 ALS, G1316C TCC, and G4212A DAD with ULD flow cell. UV absorption was observed at 215 nm and 254 nm with a 4 nm bandwidth. Column: Agilent Zorbax Extend C18, 1.8 µm, 2.1 x 50 mm. Gradient conditions: 5% to 95% CH_3CN in H_2O (0.1% formic acid) over 1 min, hold at 95% CH_3CN for 0.1 min, 0.5 mL/min, 40 ºC. For compounds that were purified on a Gilson preparative reversed-phase HPLC, the system comprised of a 333 aqueous pump with solvent selection valve, 334 organic pump, GX 271 or GX-281 liquid hander, two column switching valves, and a 155 UV detector. UV wavelength for fraction collection was user-defined, with absorbance at 254 nm always monitored. Method 1: Phenomenex Axia-packed Luna C18, 30 x 50 mm, 5 µm column. Mobile phase: CH_3CN in H_2O (0.1% TFA). Gradient conditions: 0.75 min equilibration, followed by user defined gradient (starting organic percentage, ending organic percentage, duration), hold at 95% CH_3CN in H_2O (0.1% TFA) for 1 min, 50 mL/min, 23 ºC. Method 2: Phenomenex Axia packed Gemini C18, 50 x 250 mm, 10 um column. Mobile phase: CH_3CN in H_2O (0.1% TFA). Gradient conditions: 7 min equilibration, followed by user defined gradient (starting organic percentage, ending organic percentage, duration), hold at 95% CH_3CN in H_2O (0.1% TFA) for 7 min, 120 mL/min, 23 ºC. Solvents for extraction, washing and chromatography were HPLC grade.

<u>Synthetic Procedures:</u>

Scheme S1. Synthesis of 7-chloro-1-methyl-1*H*-pyrrolo[3,2-*b*]pyridine-5-carbonitrile **13** and a 7-chlorothienyl[3,2-*b*]pyrdine-5-carbonitrile **14**.[a]

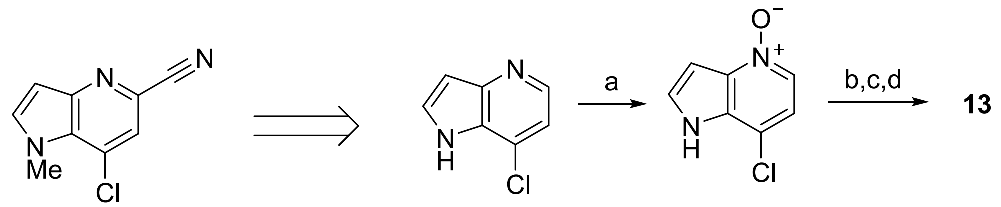

13 **15** **16**

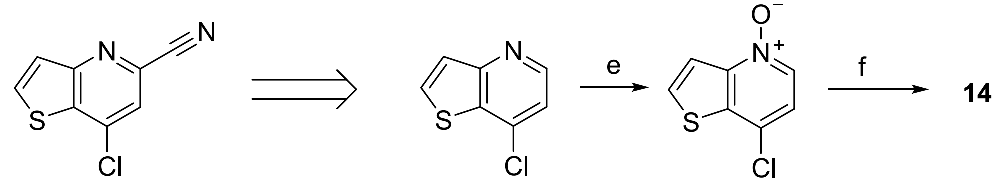

14 **17** **18**

[a]Reagents and conditions: (a) *m*-CPBA, *n*-BuOAc:heptane (3:5), 0 °C – rt, 83%; (b) $(MeO)_2SO_2$, *n*-BuOAc, 75 °C, 16 h; (c) KCN, aq. NH_4Cl, 50 °C, 2 h; quantitative yield (2 steps); (d) MeI, NaH, DMF, 0 °C – rt, 98%; (e) *m*-CPBA, DCM, 0 °C – rt, 54%; (f) TMSCN, $(CH_3)_2NCOCl$, DCM, rt, 16 h, 98%.

Preparation of 1-methyl-7-(4,4,5,5-tetramethyl-1,3,2-dioxaborolan-2-yl)-1*H*-pyrrolo[3,2-*b*]pyridine-5-carbonitrile (13)

Step a: *Synthesis* **2008**, No. 2, 201–214. To a vigorously stirred suspension of 7-chloro-1*H*-pyrrolo[3,2-*b*]pyridine (**15**, 10.0 g, 65.54 mmol) in *n*-butyl acetate-heptane mixture (3:5 v/v, 327 mL) at 0 °C was added 3-chloroperoxybenzoic acid (18.36 g, 81.92 mmol) portion wise. The reaction mixture was vigorously stirred and allowed to warm to rt overnight. Vacuum filtration of the resulting white suspension, followed by washing the filter cake with heptane (4 × 50 mL) provided intermediate **16**, 7-chloro-1*H*-pyrrolo[3,2-*b*]pyridine 4-oxide 3-chlorobenzoic acid salt (17.75 g, 83% yield). HRMS (ESI/Q-TOF) m/z: $[M+H]^+$ Calcd for $C_8H_8ClN_3$ 169.0163; Found 169.0158.

Steps b and c: A suspension of **16** (17.75 g, 54.59 mmol) and dimethyl sulfate (6.2 mL, 65.51 mmol) in *n*-butyl acetate (100 mL) was stirred under N_2 at 75 °C for 16 h. After cooling to 4 °C, cyclohexane (30 mL) was added and the solution was allowed to settle for 2 h. The upper phase (*n*-butyl acetate/cyclohexane) was separated and discarded. The lower phase was washed with cyclohexane (30 mL), diluted with sat. soln. NH_4Cl (130 mL, 54.60 mmol) and potassium cyanide (10.66 g, 163.77 mmol) was added. The resulting mixture was stirred at 50 °C for 2 h. After cooling to rt, the precipitate was collected using vacuum filtration, washed with water and 10% $NaHCO_3$ solution and dried under vacuum oven to provide 7-chloro-1*H*-pyrrolo[3,2-*b*]pyridine-5-

carbonitrile in quantitative yield and used in the next step without further purification. ^{1}H NMR (400 MHz, DMSO) δ 7.98 (d, J = 3.1 Hz, 1H), 7.88 (s, 1H), 6.77 (d, J = 3.1 Hz, 1H), N-H proton not observable; ^{13}C NMR (101 MHz, DMSO) δ 147.96, 134.00, 127.58, 125.45, 125.10, 120.39, 118.05, 103.55; HRMS (ESI/Q-TOF) m/z: $[M+H]^+$ Calcd for $C_8H_8ClN_3$ 178.1067; Found 178.0167.

Step d: To a solution of 7-chloro-1*H*-pyrrolo[3,2-*b*]pyridine-5-carbonitrile (7.57 g, 38.36 mmol, in DMF (96 mL, 0.4 M) at 0 °C, NaH (60% dispersion in mineral oil, 1.92 g, 47.96 mmol) was added portion wise. The suspension was stirred for 15 min and iodomethane (2.99 mL, 47.96 mmol) was added dropwise. After 30 min at 0 °C, cold water (~ 50 mL) was added slowly and extracted with EtOAc (3x). The combined extracts were washed with brine, dried (Na_2SO_4), filtered and concentrated in vacuo. Purification using flash chromatography on silica gel (0-50% EtOAc/hexanes) afforded **13**, 7-chloro-1-methyl-1*H*-pyrrolo[3,2-*b*]pyridine-5-carbonitrile (7.20 g, 98% yield). ^{1}H NMR (400 MHz, $CDCl_3$) δ 7.44 – 7.38 (m, 2H), 6.73 (dd, J = 3.23, 0.7 Hz, 1H), 4.16 (s, 3H); ^{13}C NMR (101 MHz, $CDCl_3$) δ 149.29, 137.62, 126.90, 126.38, 126.30, 121.77, 117.74, 103.77, 37.09; HRMS (ESI/Q-TOF) m/z: $[M+H]^+$ Calcd for $C_9H_6ClN_3$ 192.0323, Found 192.0324; ES-MS $[M+1]^+$: 192.2.

Preparation of 7-chlorothieno[3,2-*b*]pyridine-5-carbonitrile (14)

Step e: To a solution of 7-chlorothieno[3,2-*b*]pyridine (**17**, 20.356 g, 120 mmol) in DCM (500 mL) at 0 °C was added 3-chloroperoxybenzoic acid (32.27 g, 144 mmol) portion wise. The suspension was vigorously stirred and allowed to warm to rt overnight. After cooling to 0 °C, the reaction mixture was quenched with 10% $Na_2S_2O_3$ solution and concentrated under reduced pressure. The resulting residue was re-dissolved in DCM and washed with 10% K_2CO_3 solution. The aqueous layer was back extracted with iPA/$CHCl_3$ (1:3, 3x). The combined organic layers were washed with brine, dried (Na_2SO_4), filtered and concentrated to provide intermediate **18**, 7-chlorothieno[3,2-*b*]pyridine 4-oxide (44.41 g, 54% yield) which was carried forward without further purification. ES-MS $[M+H]^+$ = 186.3.

Step f: To a solution of 7-chlorothieno[3,2-*b*]pyridine 4-oxide **18** (17.11 g, 50 mmol) in DCM (250 mL) was added dimethylcarbamoyl chloride (9.2 mL, 100 mmol) slowly. After 15 min, trimethylsilyl cyanide (12.5 mL, 100 mmol) was added over 15 min. The resulting mixture was allowed to stir at rt for 16 h. The reaction mixture was recharged with dimethylcarbamoyl chloride (4.6 mL, 50 mmol) and trimethylsilyl cyanide (6.25 mL, 50 mmol). After 3 h, the reaction mixture

was quenched with 10% K_2CO_3 solution (~50 mL) and stirred for 15 min. The layers were separated. The aqueous layer was extracted with DCM (3x). The combined extracts were washed with brine, dried (Na_2SO_4), filtered and concentrated. Purification using normal phase chromatography on silica gel (0-50% EtOAc/hexanes) to provide 7-chlorothieno[3,2-*b*]pyridine-5-carbonitrile as a yellow powder (6.4 g, 66% yield). ^{1}H NMR (400 MHz, $CDCl_3$) δ 7.99 (d, *J* = 5.6 Hz, 1H), 7.67 (d, *J* = 5.6 Hz, 1H), 7.65 (s, 1H); ^{13}C NMR (101 MHz, $CDCl_3$) δ 157.64, 139.26, 136.31, 134.43, 131.65, 125.98, 122.01, 116.91. HRMS (ESI/Q-TOF) m/z: $[M+H]^+$ Calcd for $C_8H_3ClN_2S$ 194.9778; Found 194.9778.

Scheme S2. Synthesis of VU6007496 (11).[a]

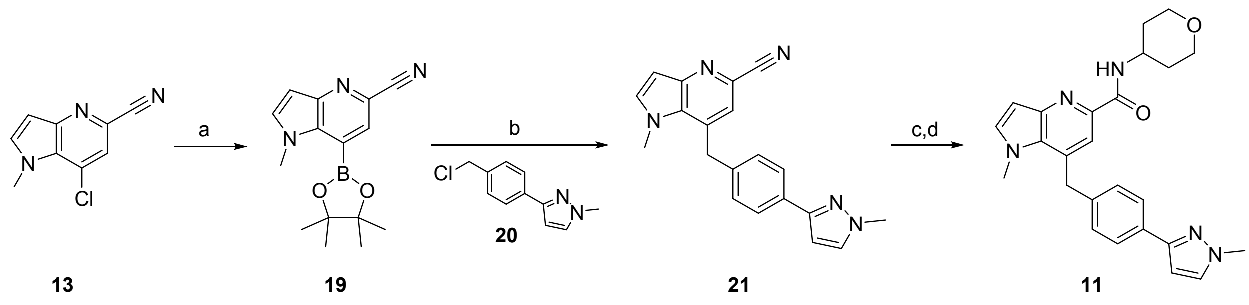

[a]Reagents and conditions: (a) bis(pinacolato)diboron, KOAc, Pd(dppf)Cl_2, 1,4-dioxane, 100 °C, 16 h; (b) benzyl chloride **20**, Cs_2CO_3, Pd(dppf)Cl_2, THF/H_2O, 100 °C, 16 h; 56% (2 steps); (c) conc. HCl, reflux, 2 h; (d) tetrahydro-2*H*-pyran-4-amine, HATU, DIEA, DMF, rt, 20 min, 56% (2 steps).

Preparation of 1-methyl-7-(4-(1-methyl-1*H*-pyrazol-3-yl)benzyl)-*N*-(tetrahydro-2*H*-pyran-4-yl)-1*H*-pyrrolo[3,2-*b*]pyridine-5-carboxamide (VU6007496)

Step a: Intermediate **13,** 7-chloro-1-methyl-1*H*-pyrrolo[3,2-*b*]pyridine-5-carbonitrile (1.92 g, 10 mmol), bis(pinacolato)diboron (3.17 g, 12.5 mmol), potassium acetate (2.94 g, 30 mmol), [1,1'-bis(diphenylphosphino)ferrocene]dichloropalladium (II) (1.10 g, 1.5 mmol) and anhydrous 1,4-dioxane (66.7 mL, 0.15 M) were charged into a RBF equipped with a Findenser™. The mixture was evacuated and purged with nitrogen (3x) and heated at 100 °C. After 16 h, the reaction mixture was filtered over a pad of Celite which was rinsed thoroughly with DCM/EtOAc (1:1). The filtrate was concentrated under reduced pressure to provide **19**, 1-methyl-7-(4,4,5,5-tetramethyl-1,3,2-dioxaborolan-2-yl)-1*H*-pyrrolo[3,2-*b*]pyridine-5-carbonitrile which was carried forward without further purification. ^{1}H NMR (400 MHz, DMSO) δ 7.93 (d, *J* = 3.3 Hz, 1H), 7.75 (s, 1H), 6.76 (d, *J* = 3.3 Hz, 1H), 3.99 (s, 3H), 1.39 (s, 12H); ES-MS $[M+H]^+$: 202.3 (boronic acid).

Step b: 1-methyl-7-(4,4,5,5-tetramethyl-1,3,2-dioxaborolan-2-yl)-1*H*-pyrrolo[3,2-*b*]pyridine-5-carbonitrile (**19**, 2.83 g, 10 mmol), cesium carbonate (9.77 g, 30 mmol), 3-(4-(chloromethyl)phenyl)-1-methyl-1*H*-pyrazole (**20**, 2.07 g, 10 mmol), anhydrous 1,4-dioxane (91 mL) and water (18 mL) were combined. After evacuated and purged with nitrogen (3x), [1,1'-bis(diphenylphosphino)ferrocene]dichloropalladium(II) (1.10 g, 1.5 mmol) was added. After 16 h at 100 °C, the reaction mixture was filtered over a pad of Celite which was rinsed thoroughly with DCM/EtOAc (1:1, v/v). The filtrate was concentrated under reduced pressure. The crude material was purified using normal phase chromatography on silica gel (0-100% EtOAc/hexanes). The material was further purified using preparative reverse phase HPLC (10-70% MeCN/0.1% TFA in H_2O) to provide 1-methyl-7-(4-(1-methyl-1H-pyrazol-3-yl)benzyl)-1H-pyrrolo[3,2-b]pyridine-5-carbonitrile (**21**, 2.46 g, 56% yield). ^{1}H NMR (400 MHz, DMSO) δ 7.78 (d, *J* = 3.3 Hz, 1H), 7.75 – 7.73 (m, 2H), 7.71 (d, *J* = 2.2 Hz, 1H), 7.41 (s, 1H), 7.15 – 7.13 (m, 2H), 6.70 (d, *J* = 3.3 Hz, 1H), 6.64 (d, *J* = 2.3 Hz, 1H), 4.58 (s, 2H), 3.94 (s, 3H), 3.86 (s, 3H); ^{13}C NMR (101 MHz, $CDCl_3$) δ 150.96, 148.46, 137.22, 136.41, 132.59, 132.20, 131.48, 129.53, 128.59 (2C), 126.44, 126.23 (2C), 123.37, 118.74, 103.34, 102.88, 39.09, 37.35, 37.09; HRMS (ESI/Q-TOF) m/z: $[M+H]^+$ Calcd for $C_{20}H_{17}N_5$ 328.1557, Found 328.1557; ES-MS $[M+1]^+$: 328.4.

Step c: A mixture of 1-methyl-7-(4-(1-methyl-1*H*-pyrazol-3-yl)benzyl)-1*H*-pyrrolo[3,2-*b*]pyridine-5-carbonitrile (**21**, 2.67 g, 5.59 mmol) and concentrated hydrochloric acid solution (37%, 20.5 mL) was refluxed for 2 h. The mixture was concentrated under reduced pressure and azeotroped with toluene (3x) to provide 1-methyl-7-(4-(1-methyl-1*H*-pyrazol-3-yl)benzyl)-1*H*-pyrrolo[3,2-*b*]pyridine-5-carboxylic acid as an HCl salt which was used without further purification. ^{1}H NMR (400 MHz, DMSO) δ 8.18 (d, *J* = 3.3 Hz, 1H), 7.80 – 7.74 (m, 3H), 7.73 (d, *J* = 2.2 Hz, 1H), 7.25 – 7.18 (m, 2H), 6.89 (d, *J* = 3.2 Hz, 1H), 6.67 (d, *J* = 2.3 Hz, 1H), 4.83 (s, 2H), 4.12 (s, 3H), 3.87 (s, 3H), COOH proton not observable; ^{13}C NMR (101 MHz, DMSO) δ 162.58, 149.55, 142.34, 141.06, 139.77, 136.90, 134.96, 132.41, 132.19, 132.15, 129.23 (2C), 125.54 (2C), 119.20, 102.54, 97.88, 38.67, 37.06, 36.09; HRMS (TOF, ES+) [M+H]+ Calcd for $C_{20}H_{18}N_4O$ 347.1503, Found 347.1507; ES-MS $[M+1]^+$: 347.4.

Step d: To a solution of carboxylic acid (1.867 g, 4.88 mmol) in DMF (49 mL) was added tetrahydro-2*H*-pyran-4-amine (5.05 mL, 48.77 mmol) followed by DIEA (4.25 mL, 24.38 mmol). The mixture was stirred for 10 min and HATU (3.71 g, 9.75 mmol) was added. The resulting mixture was stirred at rt for 20 min. Purification using preparative reverse phase HPLC (20-50%

MeCN/0.1% TFA in H_2O). The desired fractions were neutralized with sat. soln. $NaHCO_3$ and extracted with iPA/$CHCl_3$ (1:3). The combined extracts were washed with brine, dried (Na_2SO_4), filtered and concentrated to provide **11** (VU6007496) as white solid which was then converted to HCl salt using 4M HCl solution in 1,4-dioxane (1.61 g, 71% yield). ^{1}H NMR (400 MHz, MeOD) δ 8.16 (s, 1H), 8.10 (d, J = 3.3 Hz, 1H), 7.94 (d, J = 2.5 Hz, 1H), 7.85 – 7.78 (m, 2H), 7.35 – 7.29 (m, 2H), 6.97 (d, J = 3.3 Hz, 1H), 6.87 (d, J = 2.6 Hz, 1H), 4.90 (s, 2H, under H_2O peak), 4.21 (ddd, J = 11.4, 7.0, 4.3 Hz, 1H), 4.11 (s, 3H), 4.07 (s, 3H), 4.00 (ddd, J = 12.3, 4.6, 1.9 Hz, 2H), 3.53 (td, J = 11.9, 2.1 Hz, 2H), 1.95 (ddd, J = 12.5, 4.4, 2.0 Hz, 2H), 1.81 – 1.67 (m, 2H), N-H proton not observable; ^{13}C NMR (101 MHz, MeOD) δ 160.51, 150.56, 144.93, 143.78, 140.54, 139.87, 137.42, 136.38, 134.39, 130.45 (2C), 130.02, 128.06 (2C), 118.81, 105.18, 97.76, 67.88 (2C), 48.74, 38.78, 38.32, 37.77, 33.38 (2C); HRMS (TOF, ES+) [M+H]+ Calcd for $C_{25}H_{27}N_5O_2$ 430.2238, Found 430.2238; ES-MS $[M+1]^+$: 430.4.

Preparation of 3-(4-(chloromethyl)phenyl)-1-methyl-1*H*-pyrazole (20)

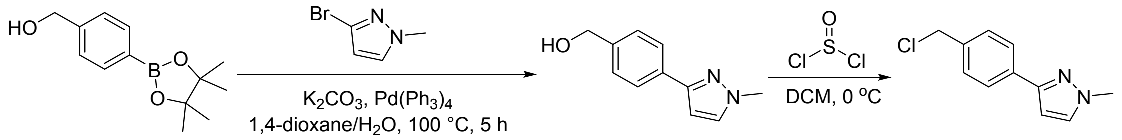

(4-(4,4,5,5-tetramethyl-1,3,2-dioxaborolan-2-yl)phenyl)methanol (CAS# 59016-93-2, 10.0 g, 65.8 mmol), 3-bromo-1-methyl-1*H*-pyrazole (CAS# 151049-87-5, 13.24 g, 82.3 mmol) and potassium carbonate (18.46 g, 131.6 mmol) were combined. Anhydrous 1,4-dioxane (300 mL) and water (75 mL) were added. The resulting mixture was evacuated and purged with nitrogen (3x) and tetrakis(triphenylphosphine)palladium (1.44 g, 1.97 mmol) was added. After 5 h at 100 °C, the reaction mixture was filtered through a pad of Celite. The filtrate was diluted with water and extracted with EtOAc (3x). The combined extracts were washed with brine, dried ($MgSO_4$), filtered and concentrated. Purification using normal phase chromatography on silica gel (0-100% EtOAc/hexanes) to provide **20a** (8.5 g, 69% yield) as a cream solid. ^{1}H NMR (400 MHz, DMSO) δ 7.76 – 7.71 (m, 2H), 7.71 (d, J = 2.2 Hz, 1H), 7.35 – 7.28 (m, 2H), 6.65 (d, J = 2.3 Hz, 1H), 5.17 (t, J = 5.7 Hz, 1H), 4.50 (d, J = 5.7 Hz, 2H), 3.87 (s, 3H); ^{13}C NMR (101 MHz, DMSO) δ 149.93, 141.55, 132.22, 131.96, 126.71 (2C), 124.69 (2C), 102.34, 62.73, 38.61; HRMS (ESI/Q-TOF) m/z: $[M+H]^+$ Calcd for $C_{11}H_{12}N_2O$ 189.1022, Found 189.1020.

To a suspension of **20a** (8.5 g, 45.16 mmol) in DCM (226 mL) at 0 °C, thionyl chloride (6.6 mL, 90.32 mmol) was slowly added. The reaction mixture was allowed to warm to rt and stirred for 2

h then diluted with DCM and washed with sat. soln. $NaHCO_3$. The aqueous layer was back extracted with DCM (2x). The combined extracts were concentrated and purified using normal phase chromatography on silica gel (0-10% MeOH/DCM) to provide **20,** 3-(4-(chloromethyl)phenyl)-1-methyl-1H-pyrazole as a white solid. ^1H NMR (400 MHz, DMSO-d_6) δ 7.82 – 7.75 (m, 2H), 7.73 (d, J = 2.3 Hz, 1H), 7.48 – 7.41 (m, 2H), 6.70 (d, J = 2.3 Hz, 1H), 4.77 (s, 2H), 3.88 (s, 3H); ^{13}C NMR (101 MHz, DMSO-d_6) δ 149.41, 136.48, 133.50, 132.41, 129.23 (2C), 125.13 (2C), 102.72, 46.15, 38.68; HRMS (ESI/Q-TOF) m/z: $[M+H]^+$ Calcd for $C_{11}H_{11}ClN_2$ 207.0684, Found 207.0682.

Scheme S3. Synthesis of VU6006874 (12).[a]

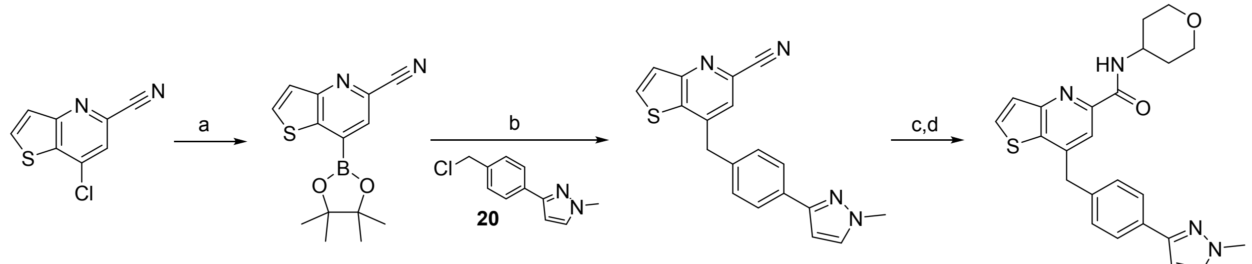

14 22 23 12

[a]Reagents and conditions: (a) bis(pinacolato)diboron, KOAc, Pd(dppf)Cl_2, 1,4-dioxane, 100 °C, 16 h, 85%; (b) benzyl chloride **20**, Cs_2CO_3, Pd(dppf)Cl_2, THF/H_2O, 90 °C, 16 h; (c) conc. HCl, 100 °C, 3 h; (d) tetrahydro-*2H*-pyran-4-amine, HATU, DIEA, DMF, rt, 1 h, 61% (2 steps).

Preparation of 7-(4-(1-methyl-1*H*-pyrazol-3-yl)benzyl)-N-(tetrahydro-2*H*-pyran-4-yl)thieno[3,2-*b*]pyridine-5-carboxamide (12, VU6006874)

Step a: Intermediate **14,** 7-chlorothieno[3,2-*b*]pyridine-5-carbonitrile (9.0 g, 46.24 mmol), bis(pinacolato)diboron (23.48 g, 92.4 mmol), potassium acetate (22.69 g, 231.2 mmol), [1,1'-bis(diphenylphosphino)ferrocene]dichloropalladium (II) (1.6 g, 2.31 mmol) and anhydrous 1,4-dioxane (231 mL, 0.2 M) were combined. The mixture was evacuated and purged with nitrogen (3x) and heated at 90 °C. After 16 h, the reaction mixture was filtered over a pad of Celite which was rinsed thoroughly with DCM. The filtrate was concentrated under reduced pressure to provide intermediate **22**, 7-(4,4,5,5-tetramethyl-1,3,2-dioxaborolan-2-yl)thieno[3,2-*b*]pyridine-5-carbonitrile (11.2 g, 85% yield) which was carried forward without further purification. HRMS (TOF, ES+) [M+H]+ Calcd for $C_8H_5BN_2O_2S$ (boronic acid) 205.0239, Found 205.0240.

Step b: 7-(4,4,5,5-tetramethyl-1,3,2-dioxaborolan-2-yl)thieno[3,2-*b*]pyridine-5-carbonitrile (**22**, 6.38 g, 22.3 mmol), cesium carbonate (36.32 g, 111.46 mmol), 3-(4-(chloromethyl)phenyl)-1-

methyl-1*H*-pyrazole (4.6 g, 22.3 mmol), anhydrous 1,4-dioxane (55.73 mL) and water (55.73 mL) were combined. After the flask was evacuated and purged with nitrogen (3x), [1,1'-bis(diphenylphosphino)ferrocene]dichloropalladium(II) (2.0 g, 2.45 mmol) was added. After 16 h at 100 °C, the reaction mixture was filtered over a pad of Celite which was rinsed thoroughly with DCM/EtOAc (1:1, v/v). The filtrate was concentrated under reduced pressure. The crude material was purified using normal phase chromatography on silica gel (0-100% EtOAc/hexanes) to provide intermediate **23**, 7-(4-(1-methyl-1*H*-pyrazol-3-yl)benzyl)thieno[3,2-*b*]pyridine-5-carbonitrile (1.61 g, 29% yield). ^{1}H NMR (400 MHz, $CDCl_3$) δ 7.86 (d, *J* = 5.6 Hz, 1H), 7.81 – 7.74 (m, 2H), 7.61 (d, *J* = 5.6 Hz, 1H), 7.40 – 7.35 (m, 2H), 7.28 – 7.25 (m, 2H), 6.53 (d, *J* = 2.3 Hz, 1H), 4.28 (s, 2H), 3.95 (s, 3H); ^{13}C NMR (101 MHz, $CDCl_3$) δ 157.01, 151.06, 146.15, 136.50, 135.15, 133.11, 133.05, 131.59, 131.44, 129.71 (2C), 126.27 (2C), 125.58, 122.16, 117.93, 103.03, 40.22, 39.22; HRMS (TOF, ES+) [M+H]+ Calcd for $C_{19}H_{14}N_4S$ 331.1012, Found 331.1012.

Step c: A mixture of 7-(4-(1-methyl-1*H*-pyrazol-3-yl)benzyl)thieno[3,2-*b*]pyridine-5-carbonitrile (1.61 g, 4.87 mmol) and concentrated hydrochloric acid solution (37%, 8.4 mL) was refluxed for 2 h. The mixture was concentrated under reduced pressure and azeotroped with toluene (3x) to provide 7-(4-(1-methyl-1*H*-pyrazol-3-yl)benzyl)thieno[3,2-*b*]pyridine-5-carboxylic acid which was carried forward without further purification. ^{1}H NMR (400 MHz, DMSO) δ 8.29 (d, *J* = 5.4 Hz, 1H), 7.94 (s, 1H), 7.76 – 7.68 (m, 4H), 7.49 (s, 1H), 7.38 – 7.31 (m, 3H), 7.24 (s, 1H), 6.65 (d, *J* = 2.3 Hz, 1H), 4.38 (s, 2H), 3.86 (s, 3H), COOH not observable; ^{13}C NMR (101 MHz, DMSO) 166.11, 155.11, 149.59, 146.43, 146.07, 136.36, 135.84, 134.40, 132.43, 132.12, 129.56 (2C), 125.33 (2C), 124.67, 119.20, 102.53, 38.90, 38.65; HRMS (TOF, ES+) [M+H]+ Calcd for $C_{19}H_{14}N_4S$ 350.0958, Found 350.0960.

Step d: To a solution of 7-(4-(1-methyl-1*H*-pyrazol-3-yl)benzyl)thieno[3,2-*b*]pyridine-5-carboxylic acid (1.7 g, 4.87 mmol) in DMF (24.3 mL) was added tetrahydro-2*H*-pyran-4-amine (604 µL, 5.84 mmol) followed by DIEA (5.08 mL, 29.20 mmol). After 10 min, HATU (2.22 g, 5.84 mmol) was added. The resulting mixture was stirred at rt for 2 h. Purification using preparative reverse phase HPLC (20-50% MeCN/0.1% TFA in H_2O). The desired fractions were neutralized with sat. soln. $NaHCO_3$ and extracted with iPA/$CHCl_3$ (1:3). The combined extracts were washed with brine, dried (Na_2SO_4), filtered and concentrated to provide compound **12 (VU6008764)** as off- white solid which was then converted to HCl salt using 4M HCl solution in 1,4-dioxane (1.7 g, 75% yield). ^{1}H NMR (400 MHz, MeOD) δ 8.43 (d, *J* = 5.6 Hz, 1H), 8.28 (s,

1H), 8.06 – 8.01 (m, 1H), 7.85 – 7.78 (m, 3H), 7.56 – 7.49 (m, 2H), 6.94 (d, *J* = 2.6 Hz, 1H), 4.61 (s, 2H), 4.20 (tt, *J* = 11.0, 4.2 Hz, 1H), 4.10 (s, 3H), 4.06 – 3.96 (m, 2H), 3.55 (td, *J* = 11.9, 2.2 Hz, 2H), 2.04 – 1.91 (m, 2H), 1.85 – 1.70 (m, 2H), N-H proton not observable; ^{13}C NMR (101 MHz, DMSO) δ 163.47, 154.94, 149.66, 148.79, 145.80, 136.50, 134.99, 133.46, 132.30, 132.15, 129.47 (2C), 125.26 (2C), 124.80, 116.83, 102.45, 66.21 (2C), 45.64, 39.39, 38.63, 32.29 (2C); HRMS (TOF, ES+) [M+H]+ Calcd for $C_{24}H_{24}N_4O_2S$ 433.1693, Found 433.1690.

Scheme S4. Synthesis of metabolite VU6036463 (24).[a]

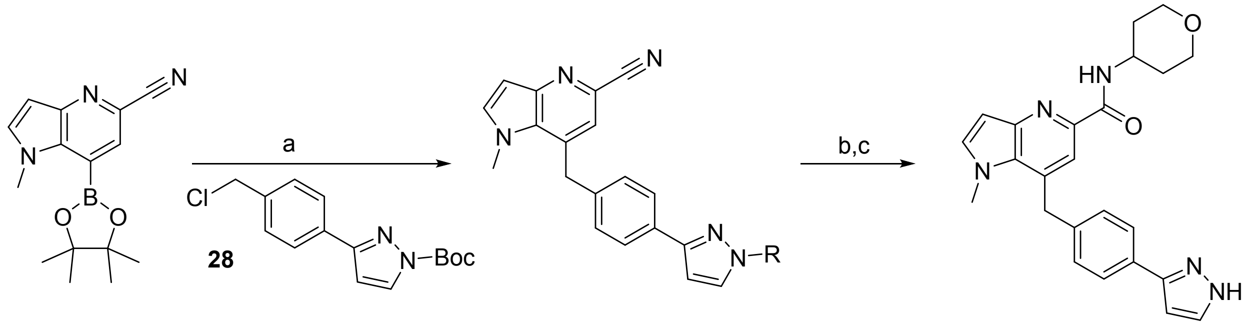

19 29, R = Boc 29a, R = H VU6306463 (**24**)

[a]Reagents and conditions: (a) benzyl chloride **28**, Cs_2CO_3, Pd(dppf)Cl_2, THF/H_2O, 90 °C, 16 h; 58%; (b) conc. HCl, reflux, 2 h; (c) tetrahydro-*2H*-pyran-4-amine, HATU, DIEA, DMF, rt, 20 min, 81% (2 steps).

Preparation of 7-(4-(1*H*-pyrazol-3-yl)benzyl)-1-methyl-*N*-(tetrahydro-2*H*-pyran-4-yl)-1*H*-pyrrolo[3,2-*b*]pyridine-5-carboxamide (VU6036463)

Step a: 1-methyl-7-(4,4,5,5-tetramethyl-1,3,2-dioxaborolan-2-yl)-1*H*-pyrrolo[3,2-*b*]pyridine-5-carbonitrile (**19**, 424 mg, 1.5 mmol), cesium carbonate (892 mg, 2.74 mmol), tert-butyl 3-(4-(chloromethyl)phenyl)-1*H*-pyrazole-1-carboxylate (**28**, 401 g, 1.37 mmol), anhydrous 1,4-dioxane (9 mL) and water (2 mL) were combined. After the flask was evacuated and purged with nitrogen (3x), [1,1'-bis(diphenylphosphino)ferrocene]dichloropalladium(II) (151 mg, 0.21 mmol) was added. After 16 h at 100 °C, the reaction mixture was filtered over a pad of Celite which was rinsed thoroughly with DCM/EtOAc (1:1, v/v). The filtrate was concentrated under reduced pressure. The crude material was purified using normal phase chromatography on silica gel (0-100% EtOAc/hexanes) and further purified using preparative reverse phase HPLC (20-80% MeCN/0.1% TFA in H_2O) to provide a mixture of tert-butyl 3-(4-((5-cyano-1-methyl-1*H*-pyrrolo[3,2-*b*]pyridin-7-yl)methyl)phenyl)-1*H*-pyrazole-1-carboxylate (**29**, 247 mg, 34% yield) and 7-(4-(1*H*-

pyrazol-3-yl)benzyl)-1-methyl-1*H*-pyrrolo[3,2-*b*]pyridine-5-carbonitrile (**29a**, 143 mg, 24% yield); ^{1}H NMR (400 MHz, DMSO) δ 7.79 (d, *J* = 3.3 Hz, 1H), 7.78 – 7.74 (m, 2H), 7.70 (d, *J* = 2.2 Hz, 1H), 7.41 (s, 1H), 7.20 – 7.14 (m, 2H), 6.69 (dd, *J* = 13.0, 2.7 Hz, 2H), 4.60 (s, 2H), 3.95 (s, 3H), NH proton not observable. HRMS (TOF, ES+) [M+H]+ Calcd for $C_{19}H_{15}N_5$ 314.1400, Found 314.1399.

Step b: A mixture of carbonitriles **29** (247 mg, 0.47 mmol) and **29a** (143 mg, 0.33 mmol) and concentrated hydrochloric acid solution (37% aq., 3.0 mL) was refluxed for 2 h. The mixture was concentrated under reduced pressure and azeotroped with toluene (3x) until dryness to provide 7-(4-(1*H*-pyrazol-3-yl)benzyl)-1-methyl-1*H*-pyrrolo[3,2-*b*]pyridine-5-carboxylic acid (295 mg) which was carried forward as HCl salt without further purification. ES-MS $[M+H]^+$ = 323.2; ^{1}H NMR (400 MHz, DMSO) δ 8.17 (d, *J* = 3.3 Hz, 1H), 7.83 – 7.78 (m, 3H), 7.73 (d, *J* = 2.2 Hz, 1H), 7.40 – 7.09 (m, 5H), 6.87 (d, *J* = 3.2 Hz, 1H), 6.71 (d, *J* = 2.2 Hz, 1H), 4.84 (s, 2H).

Step c: To a solution of 7-(4-(1*H*-pyrazol-3-yl)benzyl)-1-methyl-1*H*-pyrrolo[3,2-*b*]pyridine-5-carboxylic acid (298 mg, 0.81 mmol) in DMF (8.0 mL) was added tetrahydro-2*H*-pyran-4-amine (0.837 mL, 8.1 mmol) followed by DIEA (0.704 mL, 4.05 mmol). The mixture was stirred for 5 min and HATU (614 mg, 1.62 mmol) was added. The resulting mixture was stirred at rt for 2 h. Purification using reverse phase HPLC (20-70% MeCN/0.05% NH_4OH in H_2O) to provide VU6036463 as an off-white solid (273 mg, 81% yield). ^{1}H NMR (400 MHz, MeOD) δ 7.73 – 7.70 (m, 3H), 7.63 (s, 1H), 7.49 (d, *J* = 3.3 Hz, 1H), 7.17 (d, *J* = 8.0 Hz, 2H), 6.67 (d, *J* = 3.3 Hz, 1H), 6.63 (d, *J* = 2.3 Hz, 1H), 4.63 (s, 2H), 4.12 (tt, *J* = 10.9, 4.3 Hz, 1H), 4.01 (dd, *J* = 8.2, 4.0 Hz, 2H), 3.94 (s, 3H), 3.56 (td, *J* = 11.7, 2.2 Hz, 2H), 2.00 – 1.87 (m, 2H), 1.74 (dtd, *J* = 13.0, 11.3, 4.4 Hz, 2H), N-H proton not observable; ^{13}C NMR (101 MHz, DMSO) δ 164.09, 149.83, 145.92, 143.62, 138.41, 136.37, 132.88, 132.13, 129.79, 129.23, 128.88 (2C), 125.54 (2C), 116.87, 101.71, 101.45, 66.24 (2C), 45.40, 36.61, 36.18, 32.45 (2C); HRMS (TOF, ES+) [M+H]+ Calcd for $C_{24}H_{25}N_5O_2$ 416.2081, Found 416.2080.

Preparation of 7-(4-(1H-pyrazol-3-yl)benzyl)-1-methyl-N-(tetrahydro-2*H*-pyran-4-yl)-1*H*-pyrrolo[3,2-*b*]pyridine-5-carboxamide (30, VU6007519)

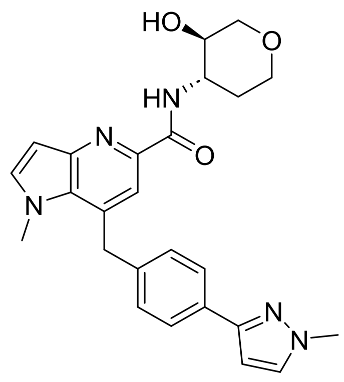

30 (VU6007519)

To a solution of 1-methyl-7-(4-(1-methyl-1*H*-pyrazol-3-yl)benzyl)-1*H*-pyrrolo[3,2-*b*]pyridine-5-carboxylic acid (52.4 mg, 0.125 mmol) in DMF (2 mL) was added (3*R*,4*S*)-4-aminotetrahydro-2*H*-pyran-3-ol (as acetyl-*D*-phenylalanine complex, 81.1 mg, 0.25 mmol) followed by DIEA (0.218 mL, 1.25 mmol). The mixture was stirred for 10 min and HATU (99.8 mg, 0.26 mmol) was added. The resulting mixture was stirred at rt for 2 h. Purification using reverse phase HPLC (20-70% MeCN/0.1% TFA in H_2O) to provide **30** (**VU6007519)** as an off-white solid (34.6 mg, 62% yield). ^{1}H NMR (400 MHz, MeOD) δ 7.74 (s, 1H), 7.72 – 7.68 (m, 2H), 7.58 (d, *J* = 2.3 Hz, 1H), 7.49 (d, *J* = 3.3 Hz, 1H), 7.17 – 7.10 (m, 2H), 6.67 (d, *J* = 3.3 Hz, 1H), 6.58 (d, *J* = 2.3 Hz, 1H), 4.61 (s, 2H), 4.02 – 3.93 (m, 6H), 3.91 (s, 3H), 3.68 (td, *J* = 9.6, 4.9 Hz, 1H), 3.54 – 3.47 (m, 1H), 3.22 (dd, *J* = 11.2, 9.8 Hz, 1H), 2.07 (ddt, *J* = 13.3, 4.6, 2.3 Hz, 1H), 1.73 (dtd, *J* = 13.3, 11.7, 4.6 Hz, 1H), N-H and O-H protons not observable; ^{13}C NMR (101 MHz, MeOD) δ 168.30, 152.65, 148.15, 144.56, 140.40, 137.47, 134.11, 133.51, 133.12, 131.48, 129.81 (2C), 127.09 (2C), 118.98, 103.89, 103.00, 72.54, 70.27, 67.70, 54.53, 38.84, 38.19, 37.18, 32.66; HRMS (Q-TOF, ES+) [M+H]+ Calcd for $C_{25}H_{27}N_5O_3$ 446.2187; Found 446.2186.

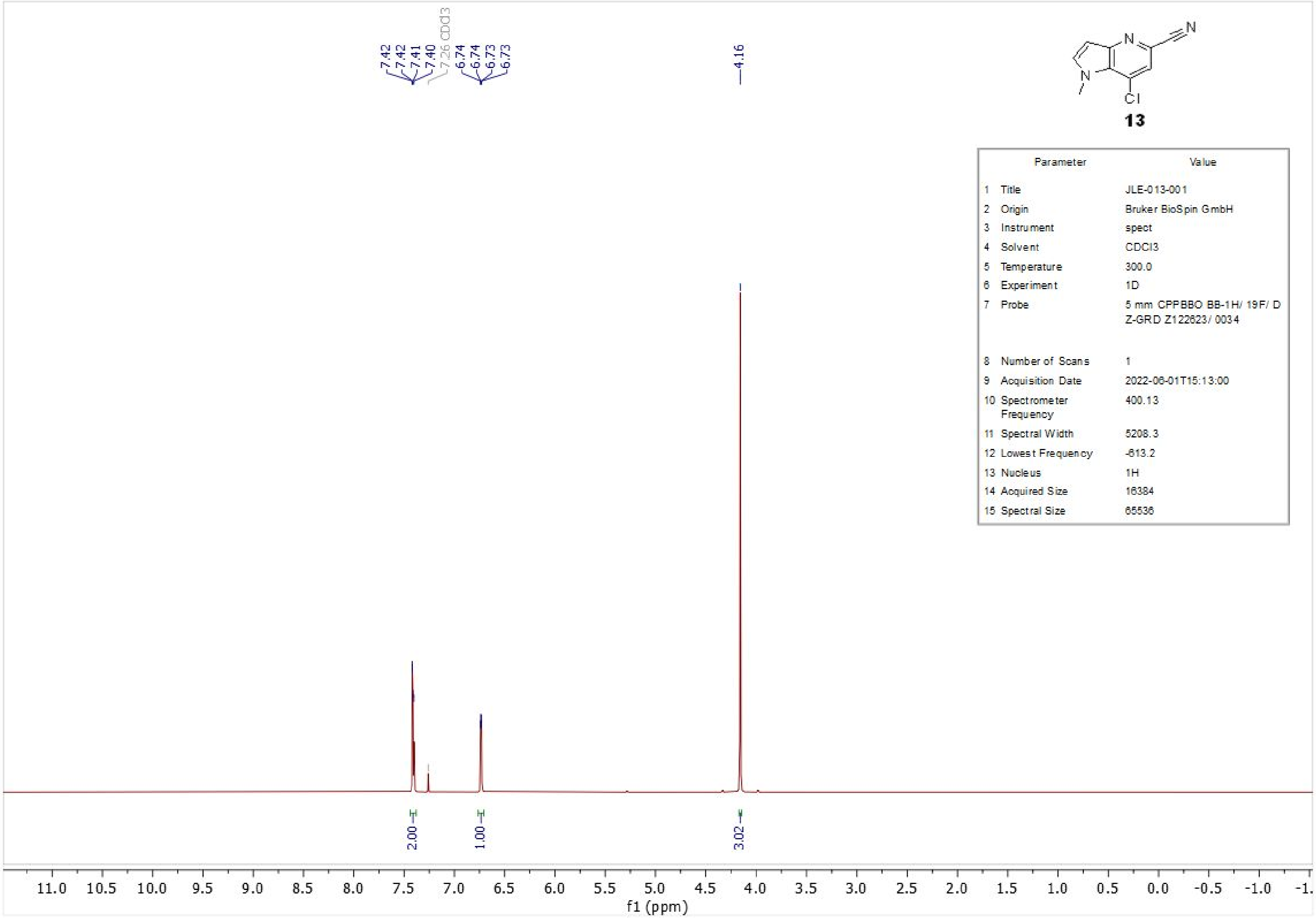

^{1}H NMR (400 MHz, $CDCl_3$) δ 7.44 – 7.38 (m, 2H), 6.73 (dd, J = 3.3, 0.7 Hz, 1H), 4.16 (s, 3H).

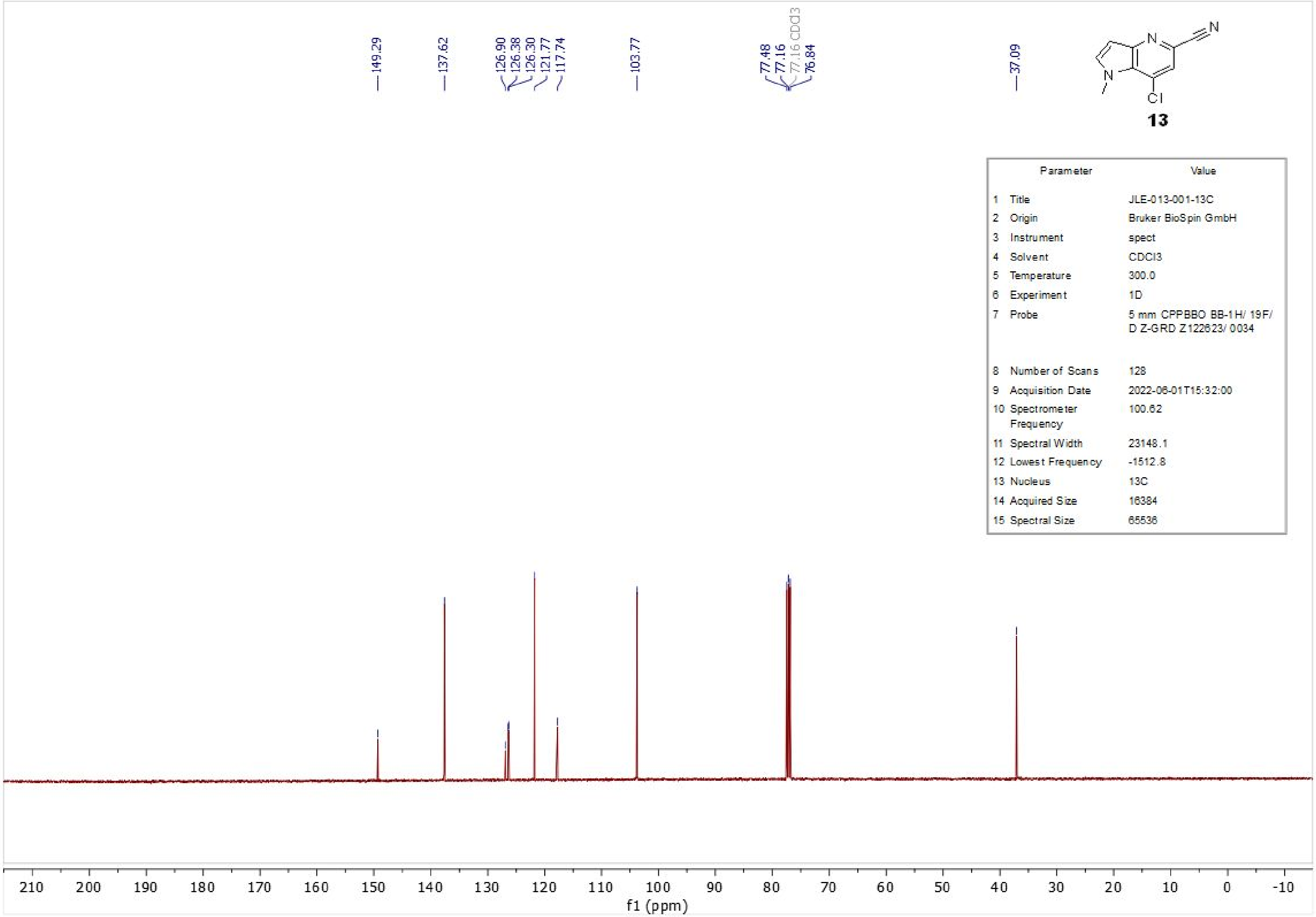

^{13}C NMR (101 MHz, $CDCl_3$) δ 149.29, 137.62, 126.90, 126.38, 126.30, 121.77, 117.74, 103.77, 77.48, 77.16, 76.84, 37.09.

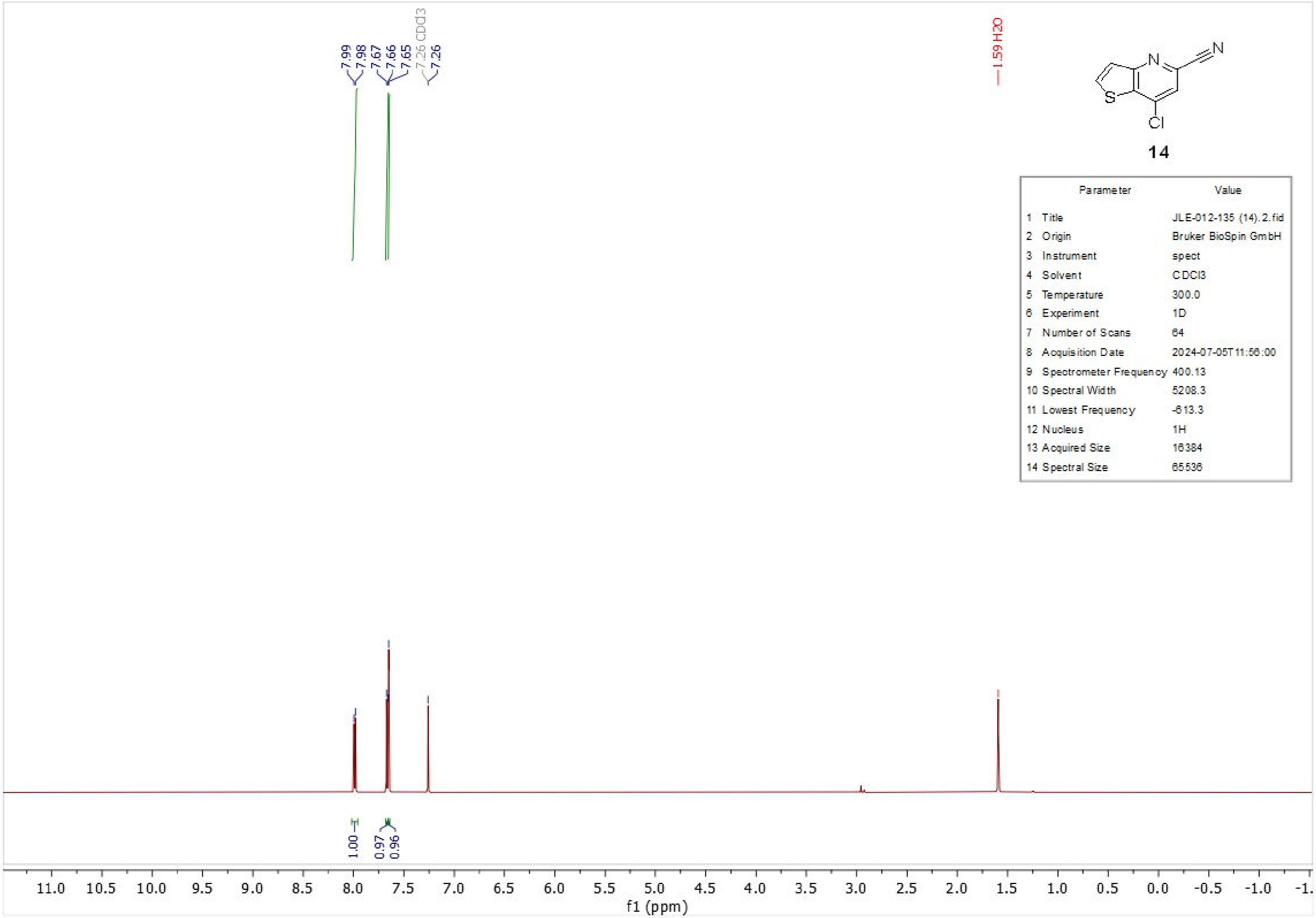

^{1}H NMR (400 MHz, $CDCl_3$) δ 7.99 (d, *J* = 5.6 Hz, 1H), 7.67 (d, *J* = 5.6 Hz, 1H), 7.65 (s, 1H).

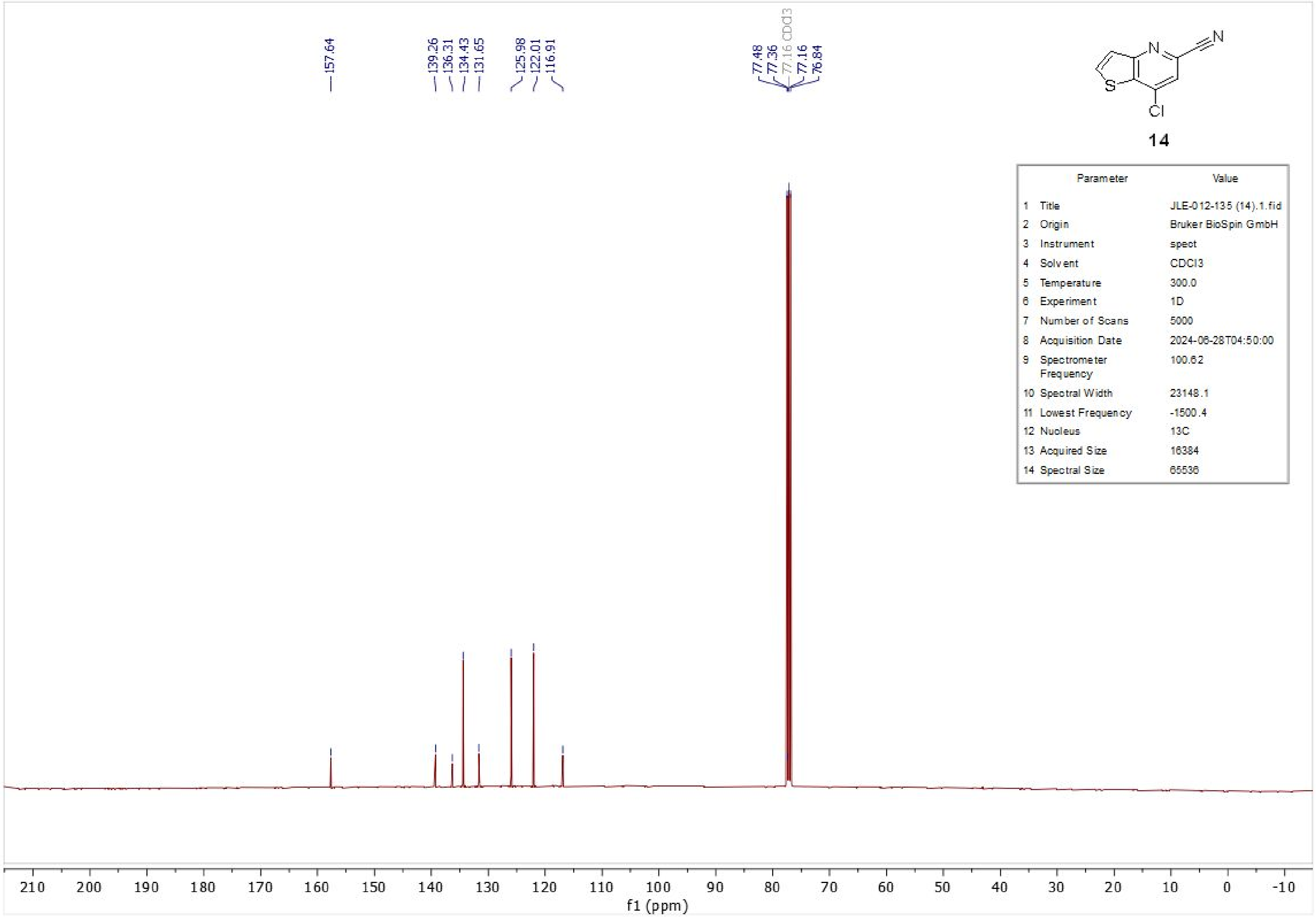

^{13}C NMR (101 MHz, $CDCl_3$) δ 157.64, 139.26, 136.31, 134.43, 131.65, 125.98, 122.01, 116.91

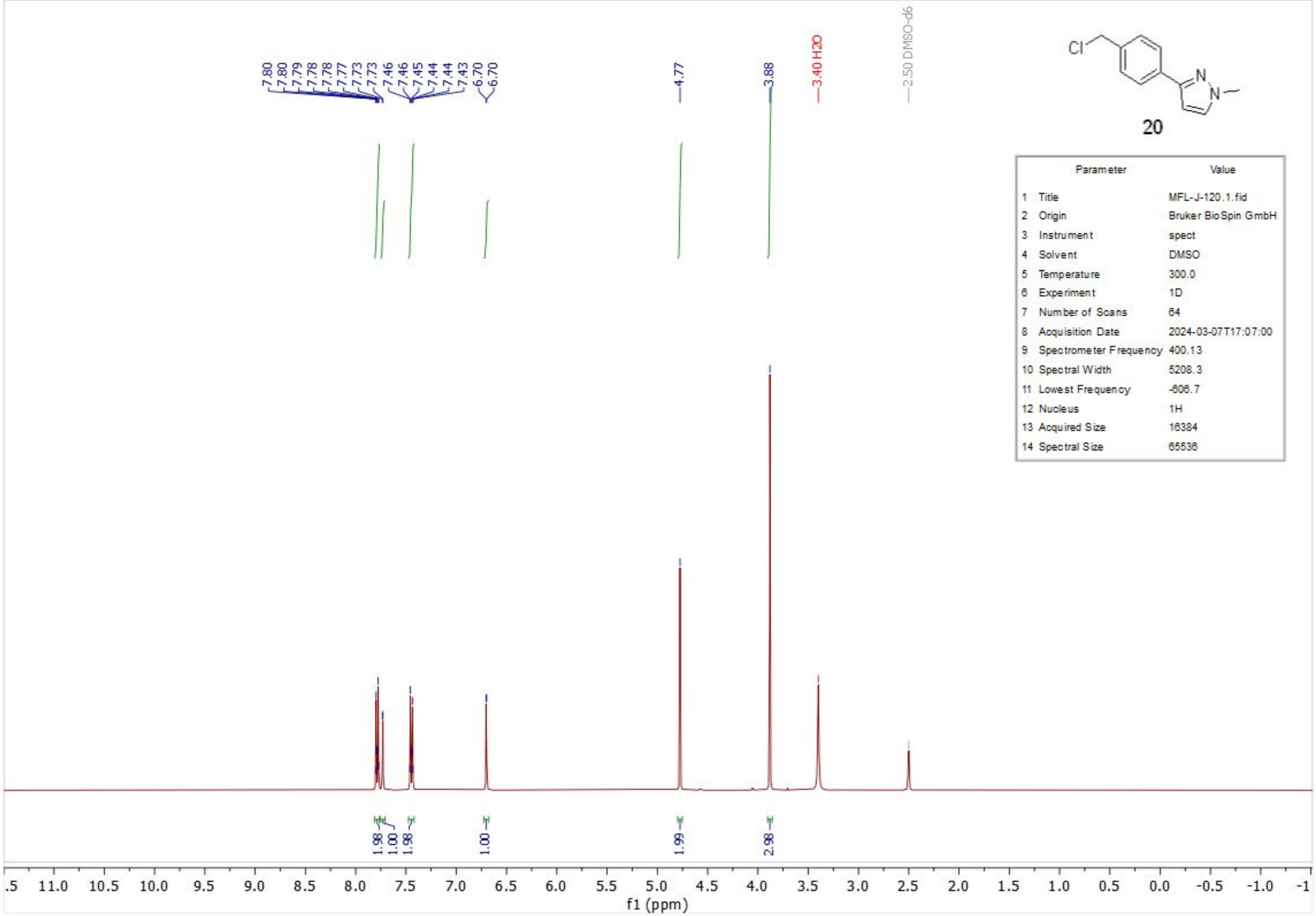

^{1}H NMR (400 MHz, DMSO-d$_6$) δ 7.82 – 7.75 (m, 2H), 7.73 (d, J = 2.3 Hz, 1H), 7.48 – 7.41 (m, 2H), 6.70 (d, J = 2.3 Hz, 1H), 4.77 (s, 2H), 3.88 (s, 3H).

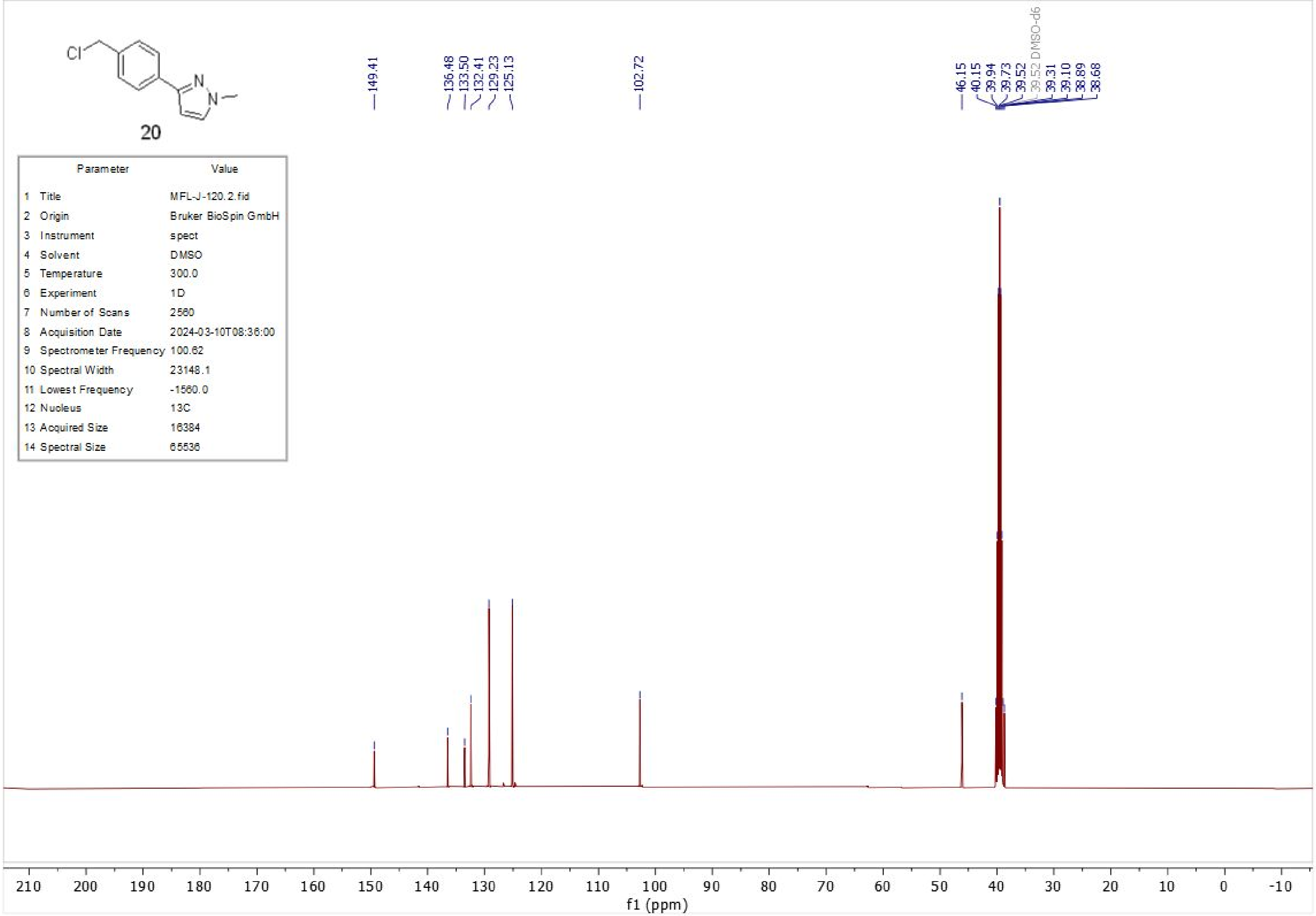

^{13}C NMR (101 MHz, DMSO-d_6) δ 149.41, 136.48, 133.50, 132.41, 129.23 (2C), 125.13 (2C), 102.72, 46.15, 38.68.

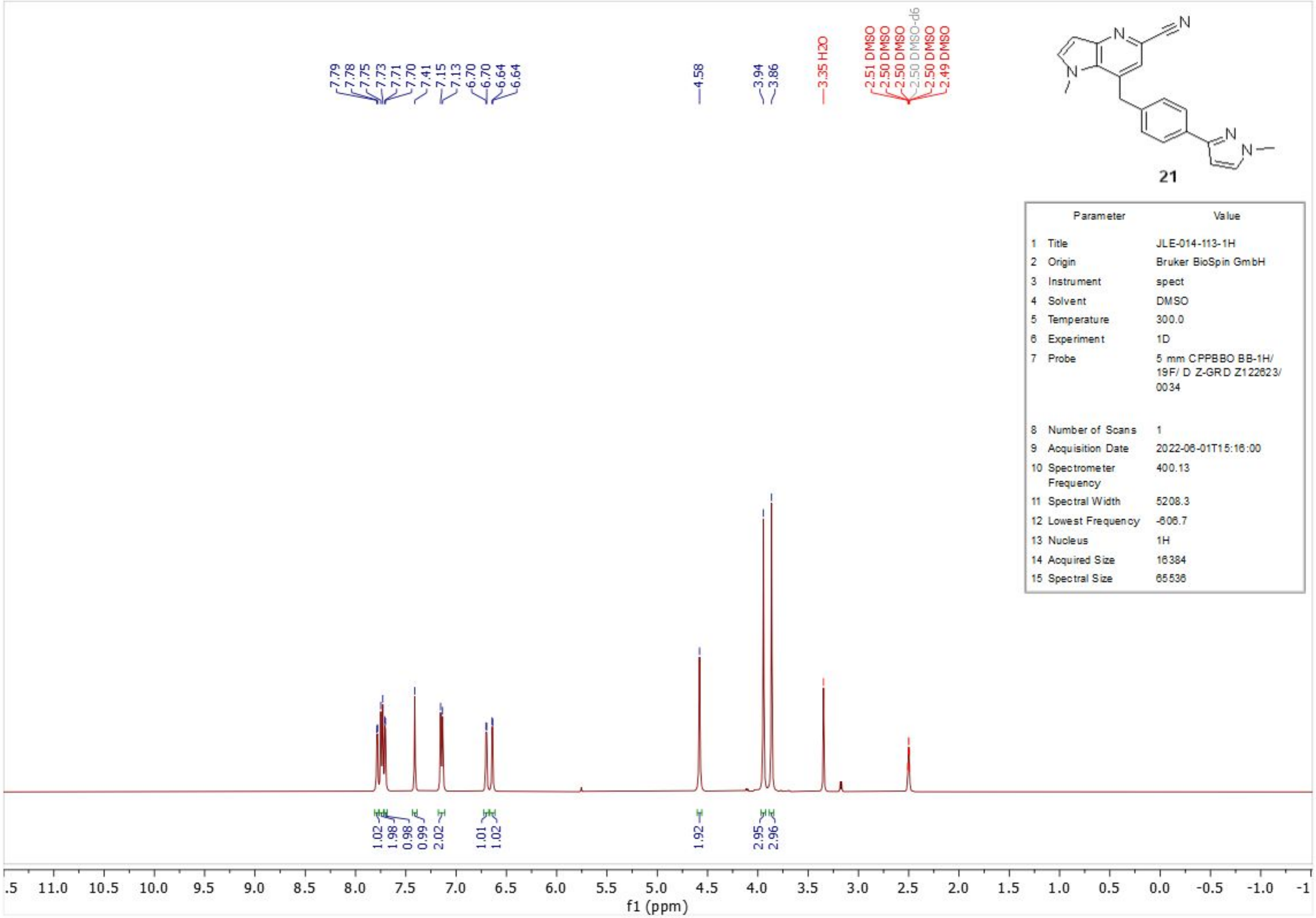

^{1}H NMR (400 MHz, DMSO) δ 7.78 (d, J = 3.3 Hz, 1H), 7.75 – 7.73 (m, 2H), 7.71 (d, J = 2.2 Hz, 1H), 7.41 (s, 1H), 7.15 – 7.13 (m, 2H), 6.70 (d, J = 3.3 Hz, 1H), 6.64 (d, J = 2.3 Hz, 1H), 4.58 (s, 2H), 3.94 (s, 3H), 3.86 (s, 3H)

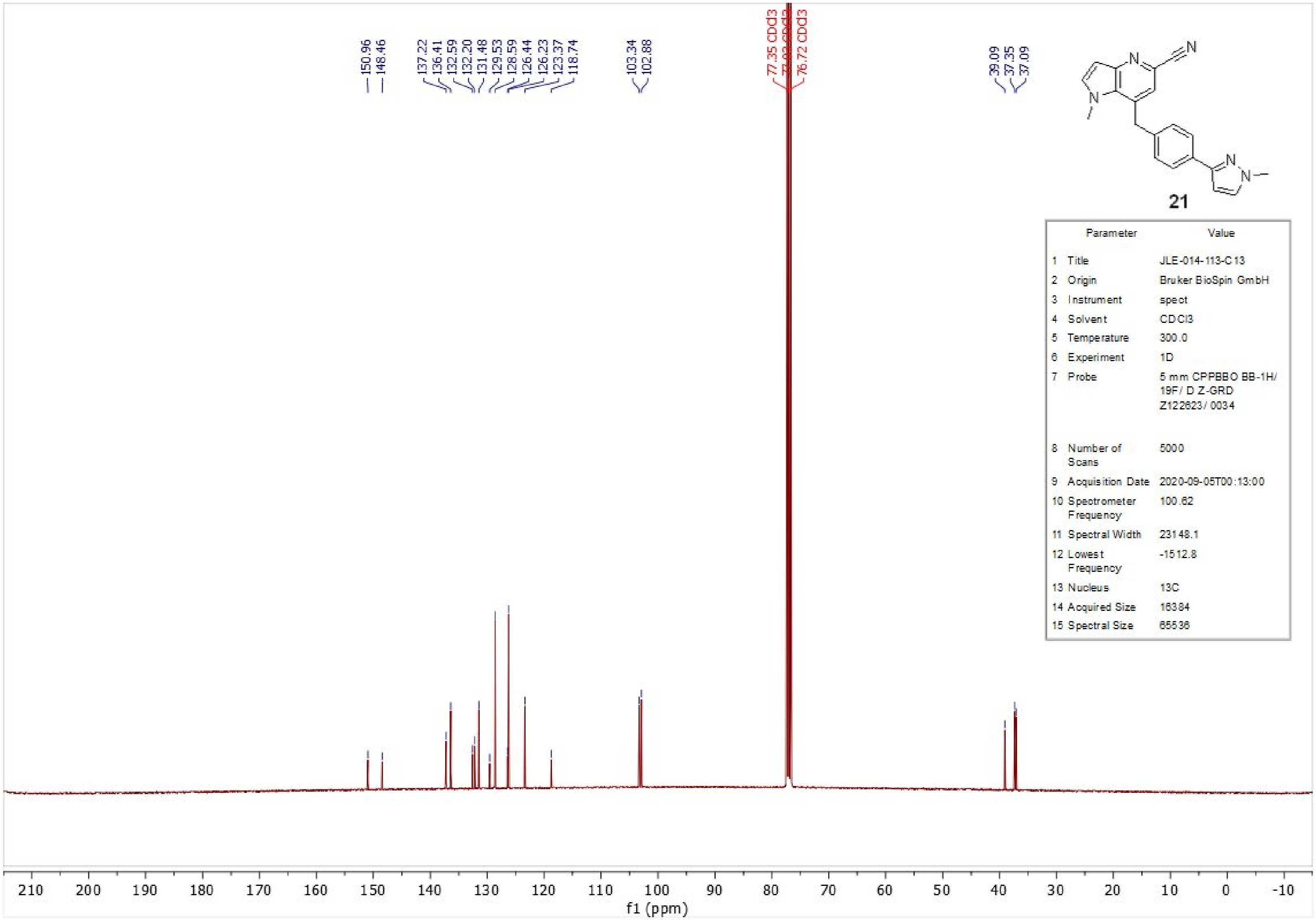

^{13}C NMR (101 MHz, $CDCl_3$) δ 150.96, 148.46, 137.22, 136.41, 132.59, 132.20, 131.48, 129.53, 128.59 (2C), 126.44, 126.23 (2C), 123.37, 118.74, 103.34, 102.88, 39.09, 37.35, 37.09.

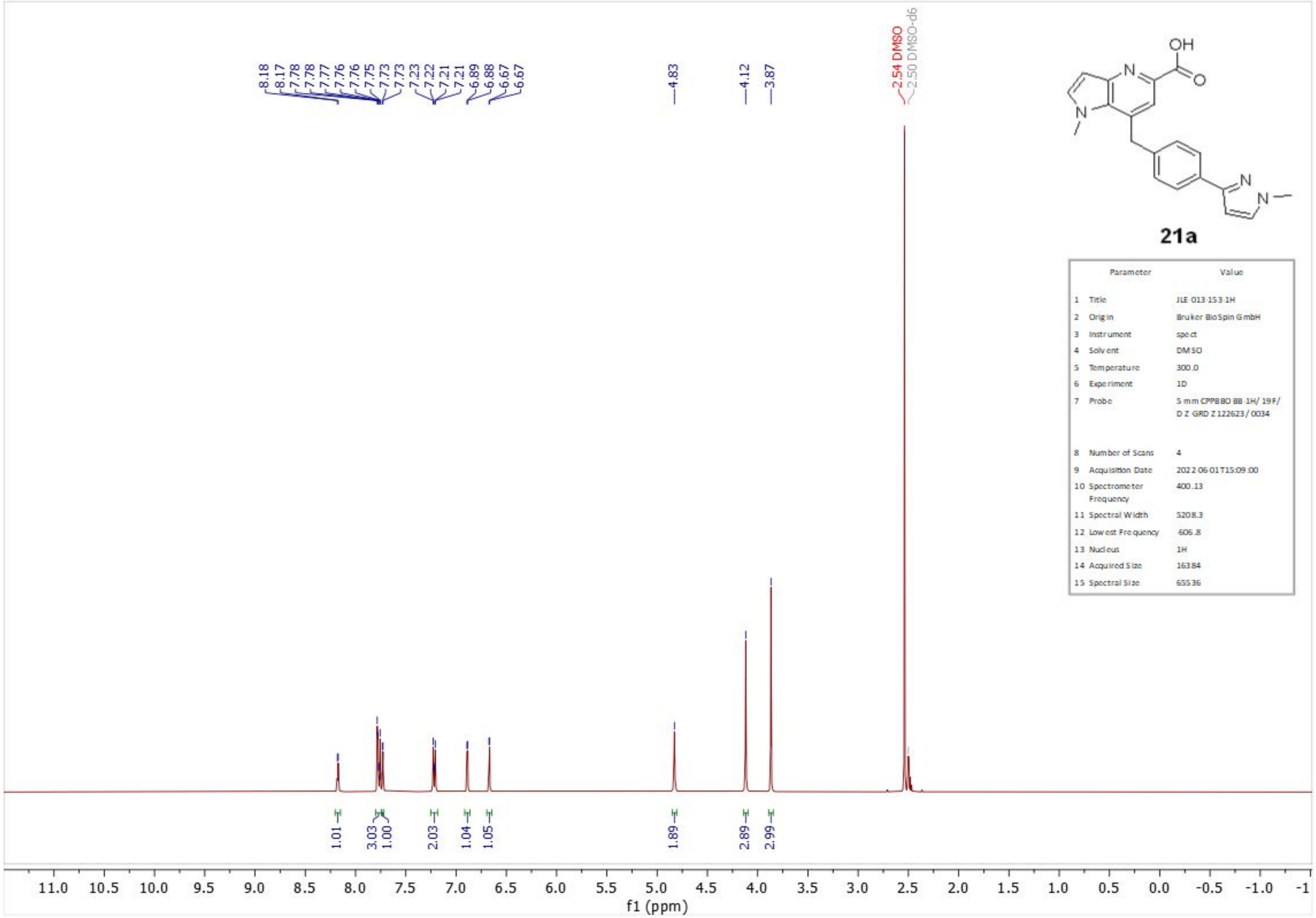

^{1}H NMR (400 MHz, DMSO) δ 8.18 (d, J = 3.3 Hz, 1H), 7.80 – 7.74 (m, 3H), 7.73 (d, J = 2.2 Hz, 1H), 7.25 – 7.18 (m, 2H), 6.89 (d, J = 3.2 Hz, 1H), 6.67 (d, J = 2.3 Hz, 1H), 4.83 (s, 2H), 4.12 (s, 3H), 3.87 (s, 3H), COOH proton not observable.

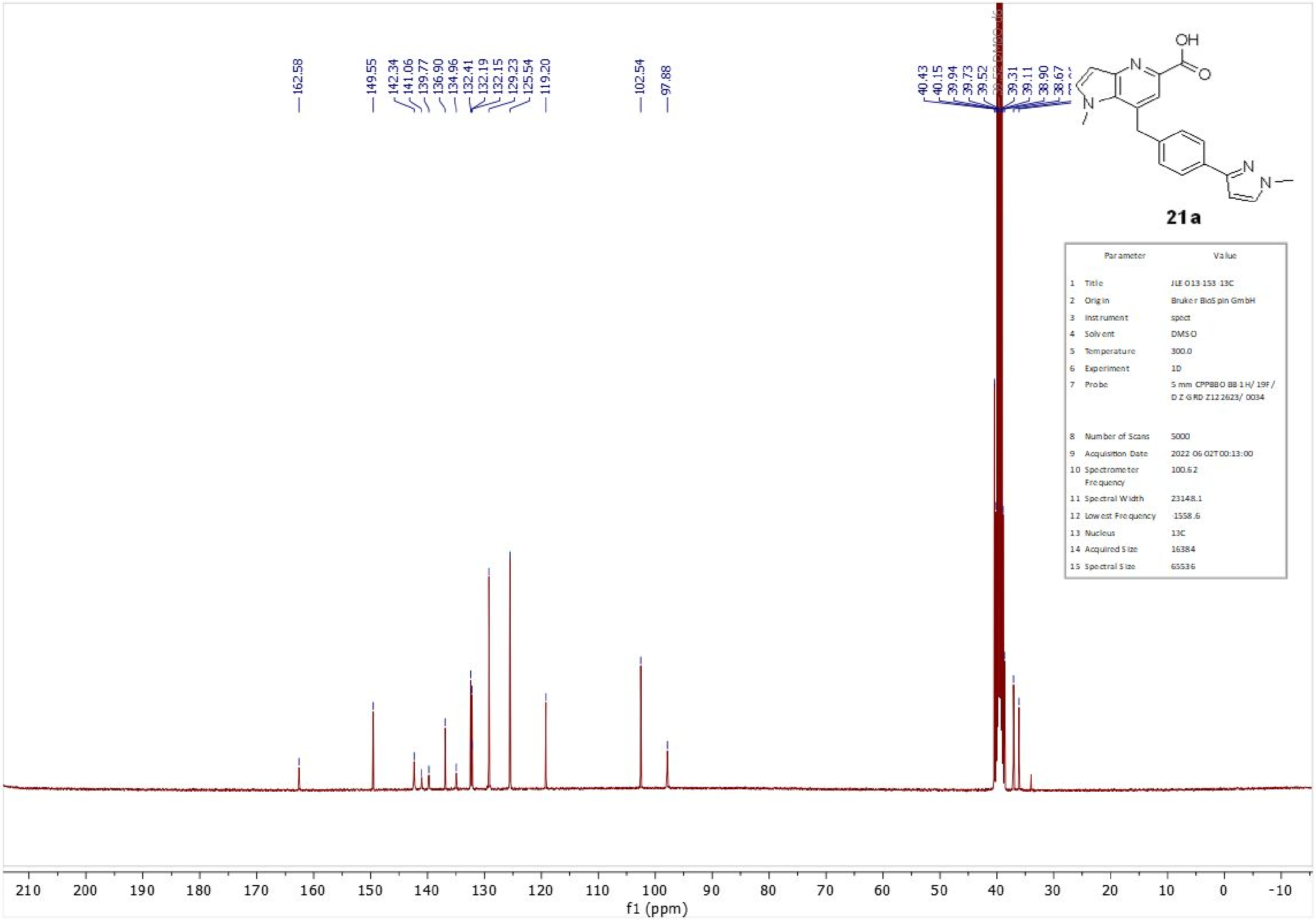

^{13}C NMR (101 MHz, DMSO) δ 162.58, 149.55, 142.34, 141.06, 139.77, 136.90, 134.96, 132.41, 132.19, 132.15, 129.23 (2C), 125.54 (2C), 119.20, 102.54, 97.88, 38.67, 37.06, 36.09

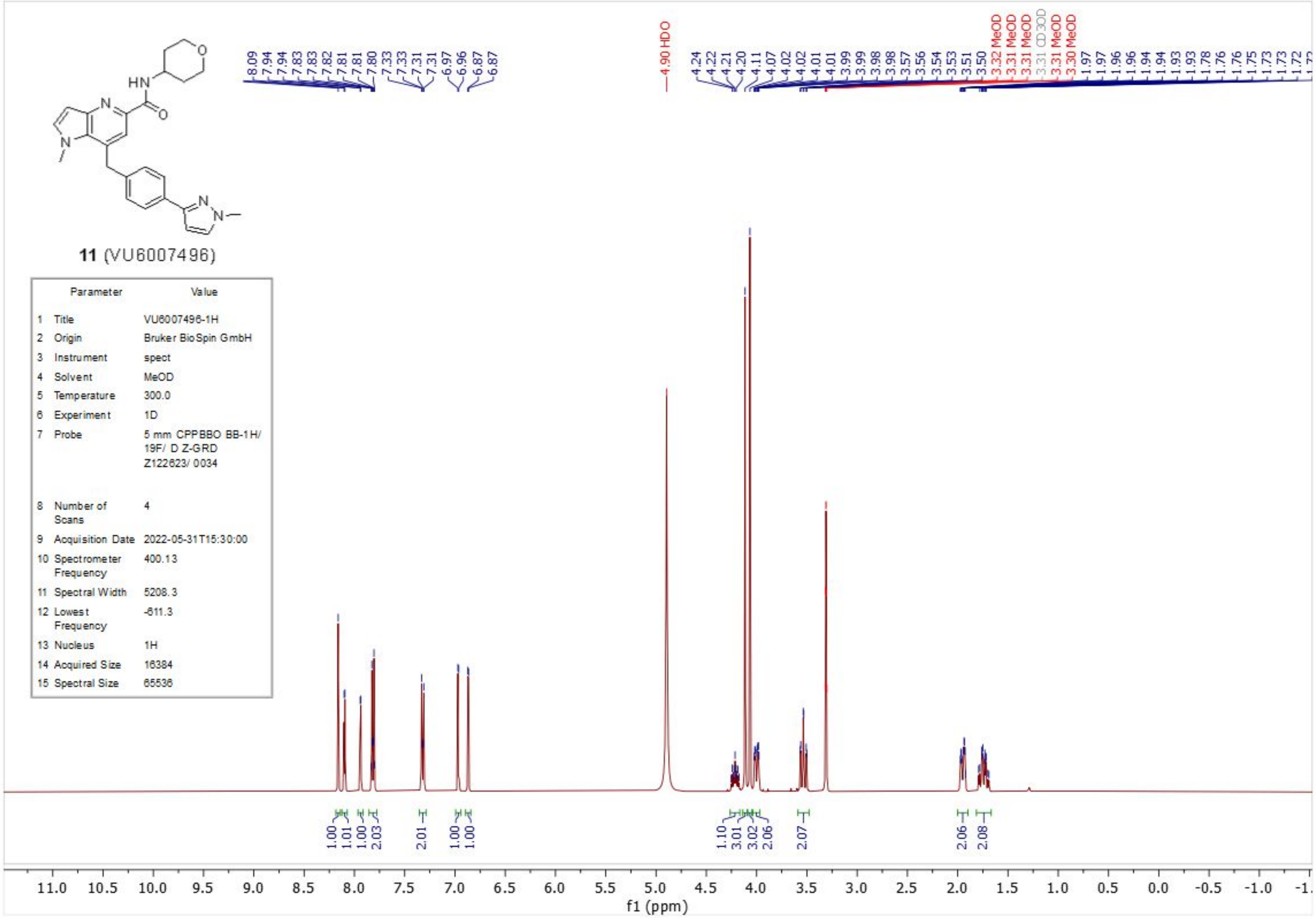

^{1}H NMR (400 MHz, MeOD) δ 8.16 (s, 1H), 8.10 (d, *J* = 3.3 Hz, 1H), 7.94 (d, *J* = 2.5 Hz, 1H), 7.85 – 7.78 (m, 2H), 7.35 – 7.29 (m, 2H), 6.97 (d, *J* = 3.3 Hz, 1H), 6.87 (d, *J* = 2.6 Hz, 1H), 4.90 (s, 2H, under H_2O peak), 4.21 (ddd, *J* = 11.4, 7.0, 4.3 Hz, 1H), 4.11 (s, 3H), 4.07 (s, 3H), 4.00 (ddd, *J* = 12.3, 4.6, 1.9 Hz, 2H), 3.53 (td, *J* = 11.9, 2.1 Hz, 2H), 1.95 (ddd, *J* = 12.5, 4.4, 2.0 Hz, 2H), 1.81 – 1.67 (m, 2H), N-H proton not observable.

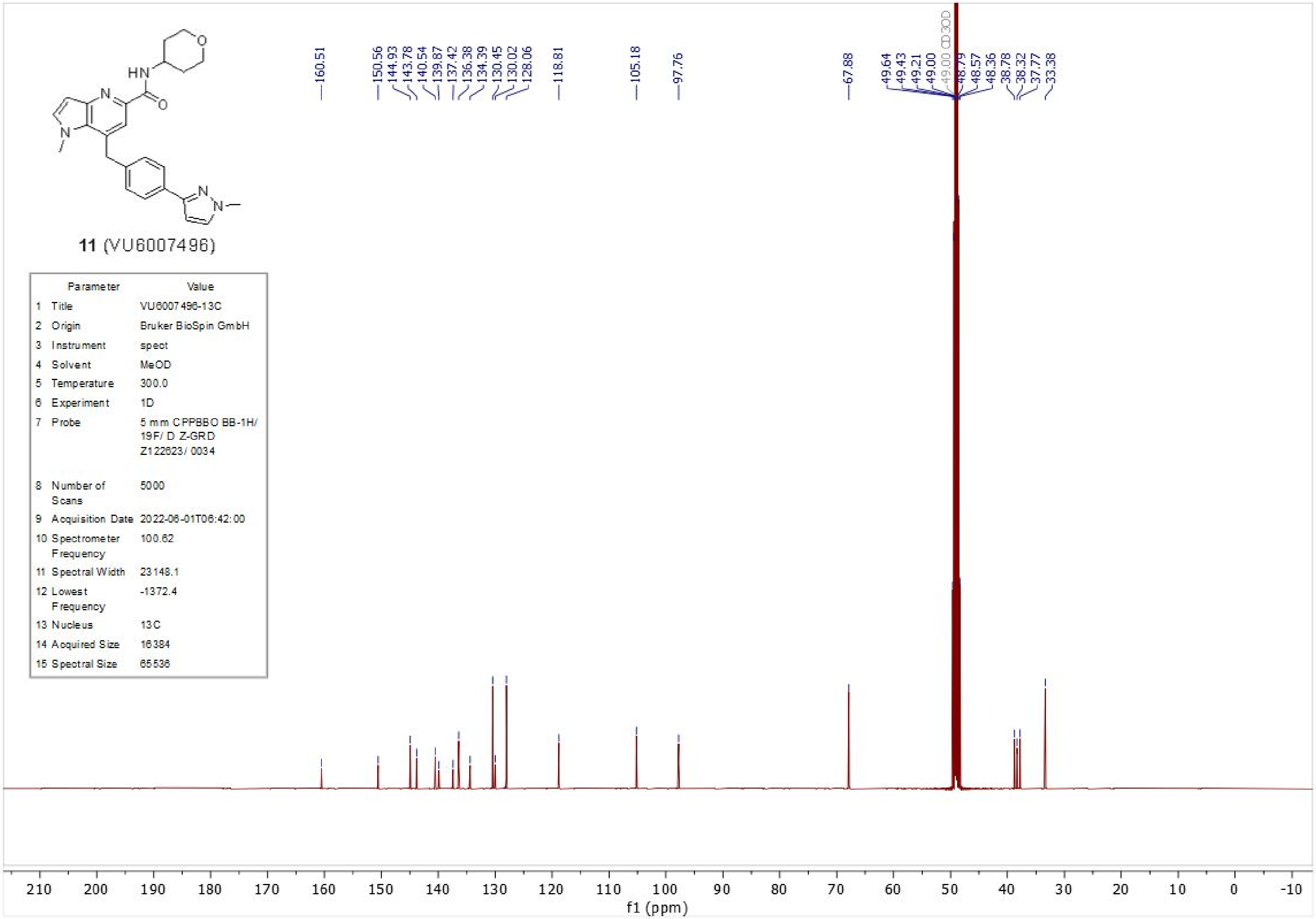

^{13}C NMR (101 MHz, MeOD) δ 160.51, 150.56, 144.93, 143.78, 140.54, 139.87, 137.42, 136.38, 134.39, 130.45 (2C), 130.02, 128.06 (2C), 118.81, 105.18, 97.76, 67.88 (2C), 47.74, 38.78, 38.32, 37.77, 33.38 (2C).

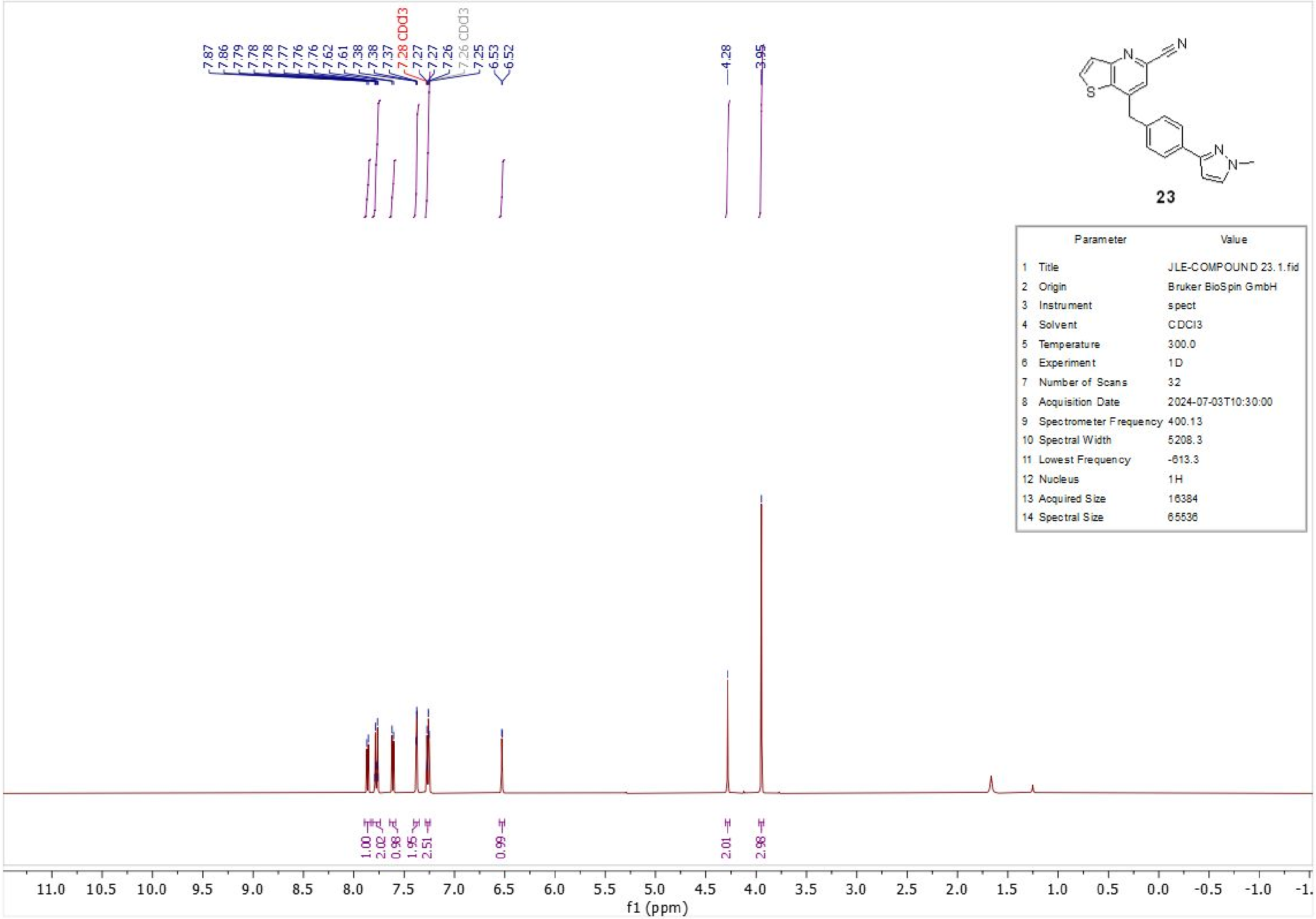

^{1}H NMR (400 MHz, $CDCl_3$) δ 7.86 (d, J = 5.6 Hz, 1H), 7.81 – 7.74 (m, 2H), 7.61 (d, J = 5.6 Hz, 1H), 7.40 – 7.35 (m, 2H), 7.28 – 7.25 (m, 2H), 6.53 (d, J = 2.3 Hz, 1H), 4.28 (s, 2H), 3.95 (s, 3H).

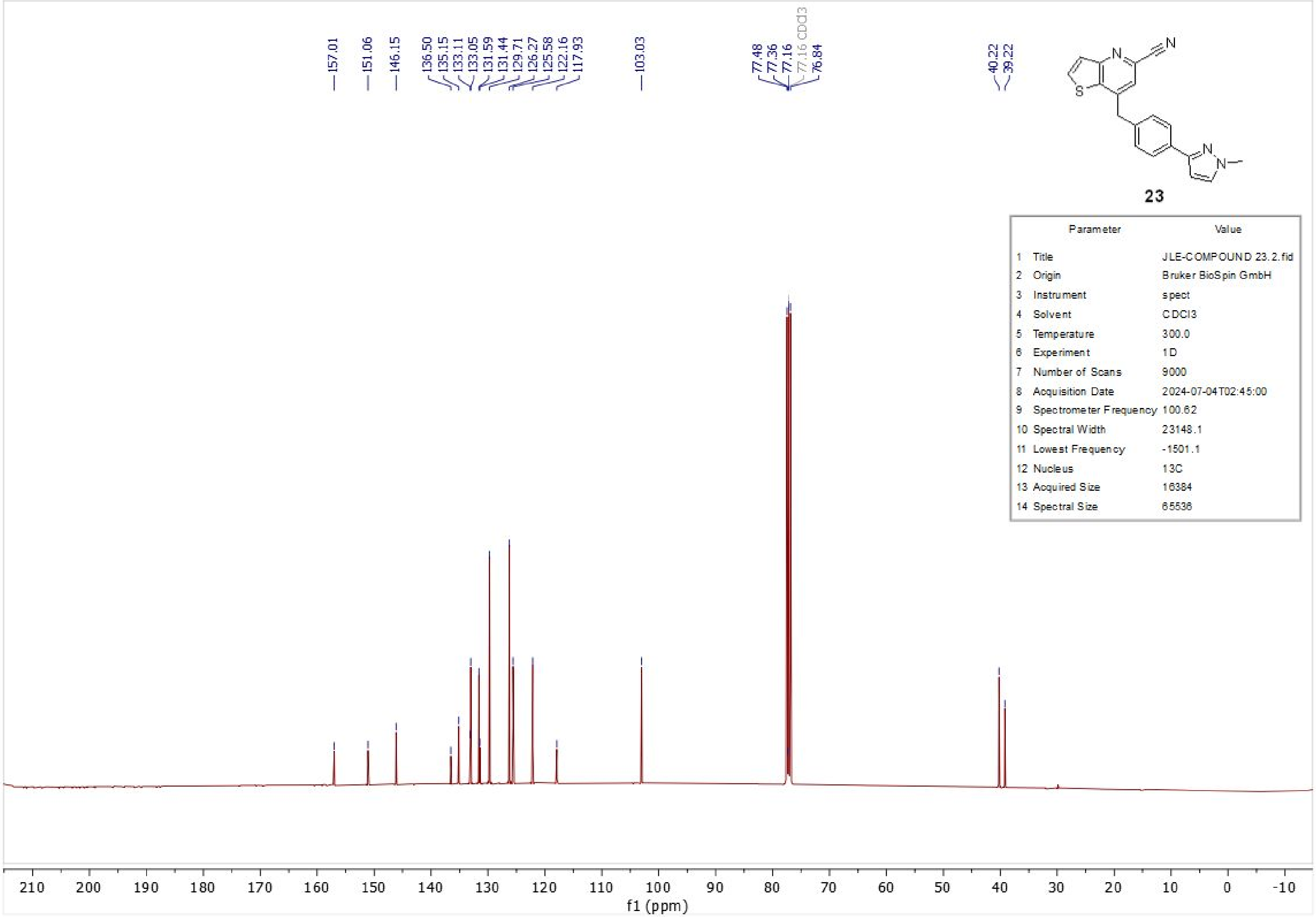

^{13}C NMR (101 MHz, $CDCl_3$) δ 157.01, 151.06, 146.15, 136.50, 135.15, 133.11, 133.05, 131.59, 131.44, 129.71 (2C), 126.27 (2C), 125.58, 122.16, 117.93, 103.03, 40.22, 39.22.

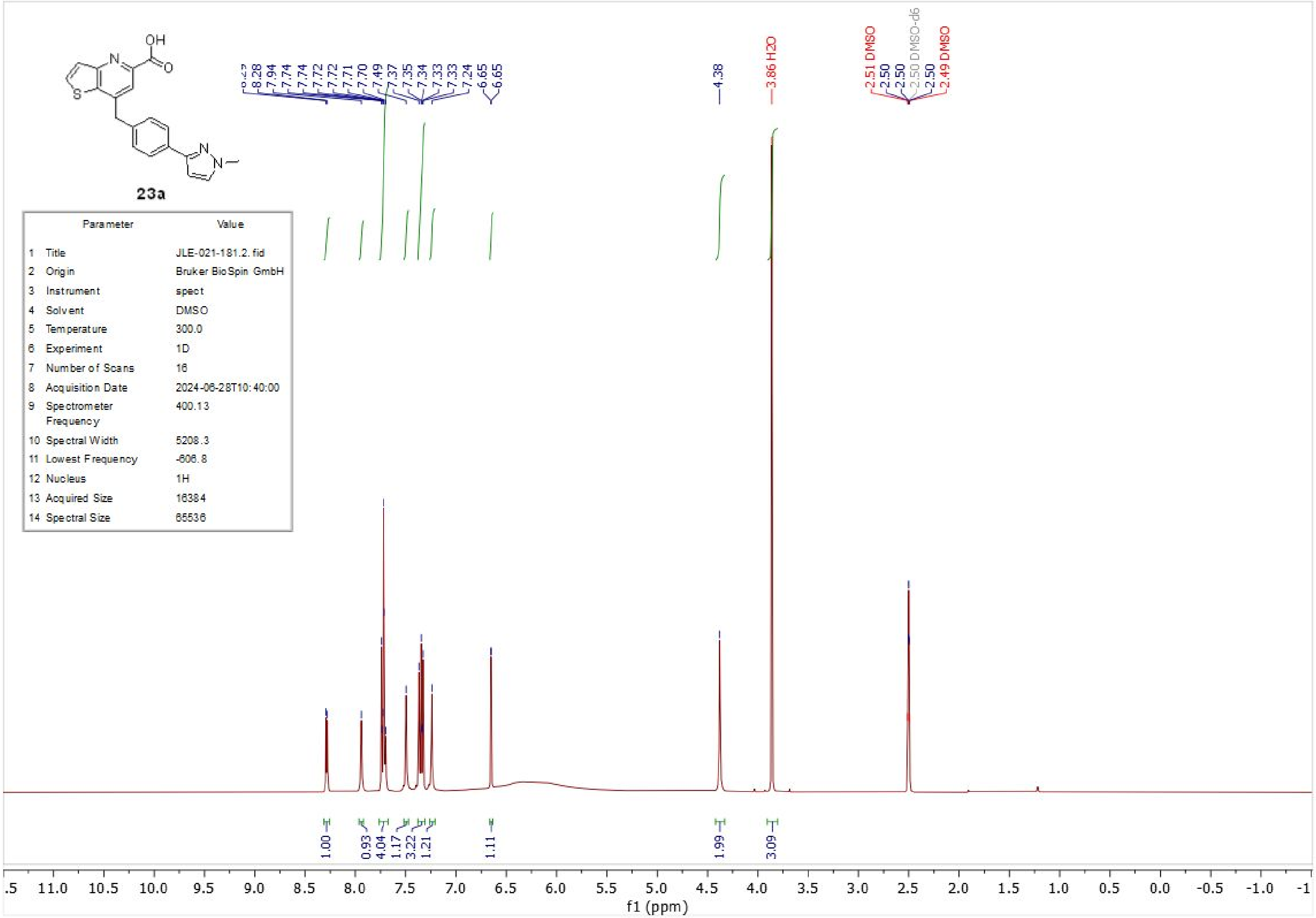

^{1}H NMR (400 MHz, DMSO) δ 8.29 (d, *J* = 5.4 Hz, 1H), 7.94 (s, 1H), 7.76 – 7.68 (m, 4H), 7.49 (s, 1H), 7.38 – 7.31 (m, 3H), 7.24 (s, 1H), 6.65 (d, *J* = 2.3 Hz, 1H), 4.38 (s, 2H), 3.86 (s, 3H), COOH not observable.

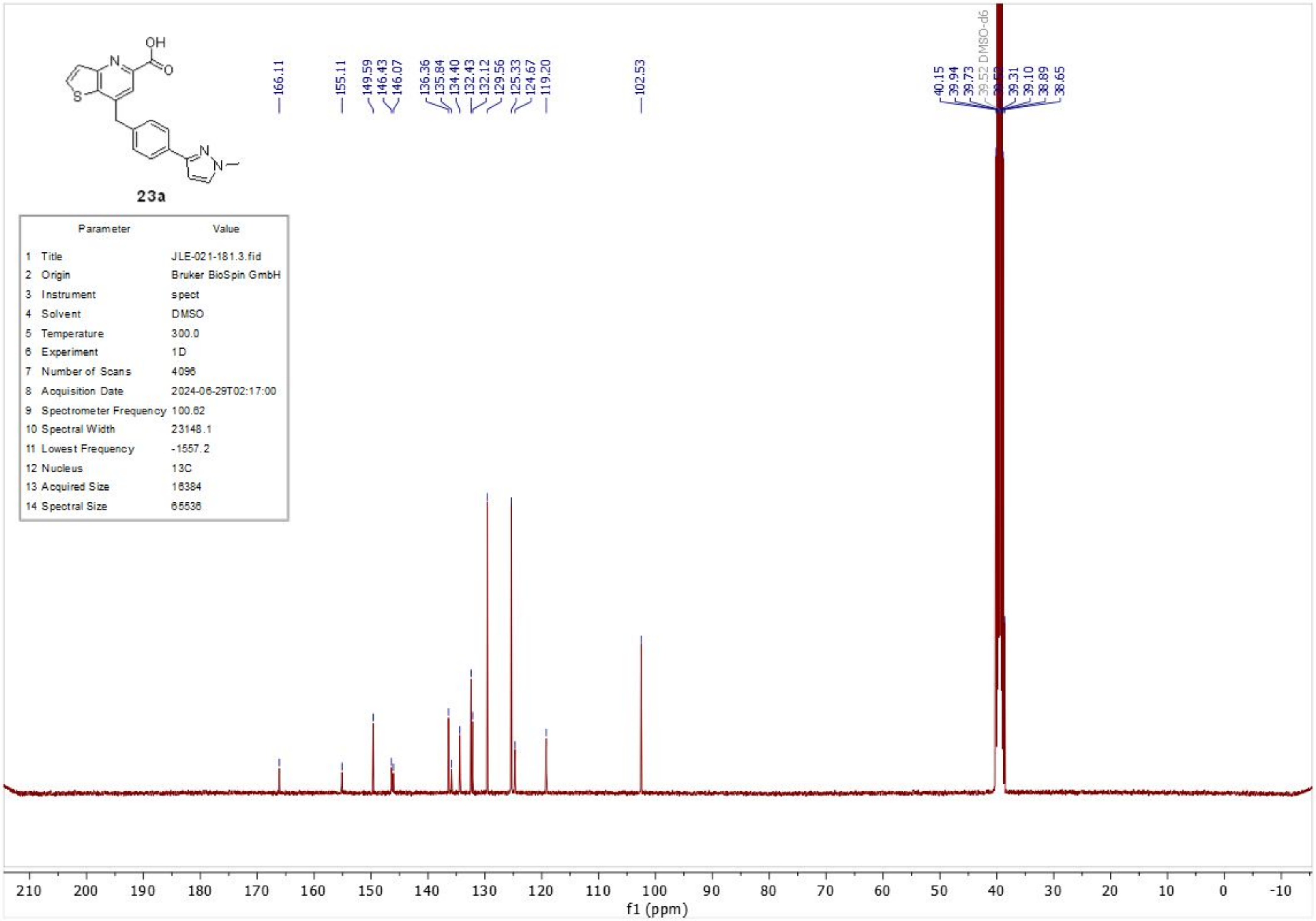

^{13}C NMR (101 MHz, DMSO) 166.11, 155.11, 149.59, 146.43, 146.07, 136.36, 135.84, 134.40, 132.43, 132.12, 129.56 (2C), 125.33 (2C), 124.67, 119.20, 102.53, 38.90, 38.65.

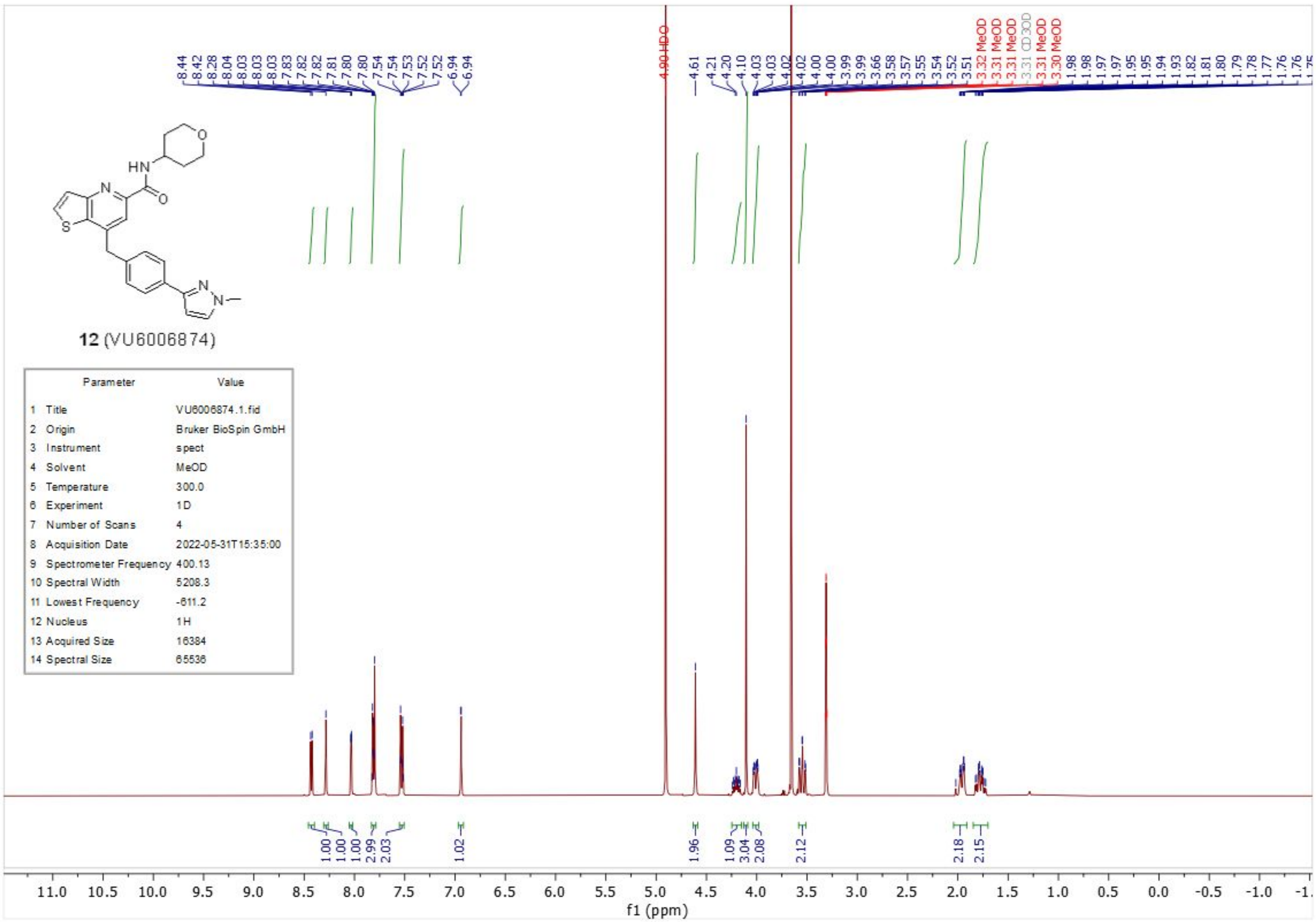

^{1}H NMR (400 MHz, MeOD) δ 8.43 (d, J = 5.6 Hz, 1H), 8.28 (s, 1H), 8.06 – 8.01 (m, 1H), 7.85 – 7.78 (m, 3H), 7.56 – 7.49 (m, 2H), 6.94 (d, J = 2.6 Hz, 1H), 4.61 (s, 2H), 4.20 (tt, J = 11.0, 4.2 Hz, 1H), 4.10 (s, 3H), 4.06 – 3.96 (m, 2H), 3.55 (td, J = 11.9, 2.2 Hz, 2H), 2.04 – 1.91 (m, 2H), 1.85 – 1.70 (m, 2H), N-H proton not observable.

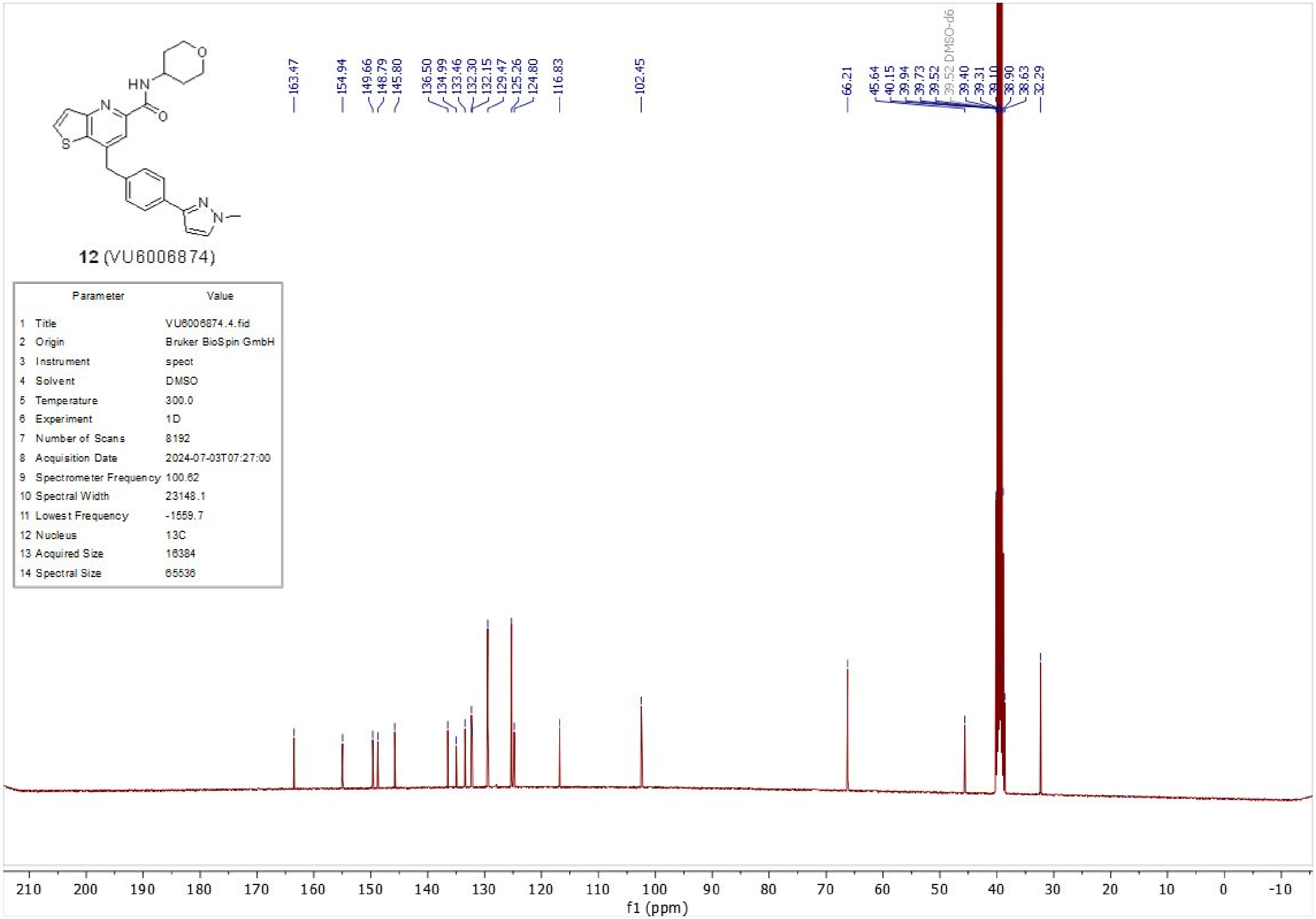

^{13}C NMR (101 MHz, DMSO) δ 163.47, 154.94, 149.66, 148.79, 145.80, 136.50, 134.99, 133.46, 132.30, 132.15, 129.47 (2C), 125.26 (2C), 124.80, 116.83, 102.45, 66.21 (2C), 45.64, 39.39, 38.63, 32.29 (2C).

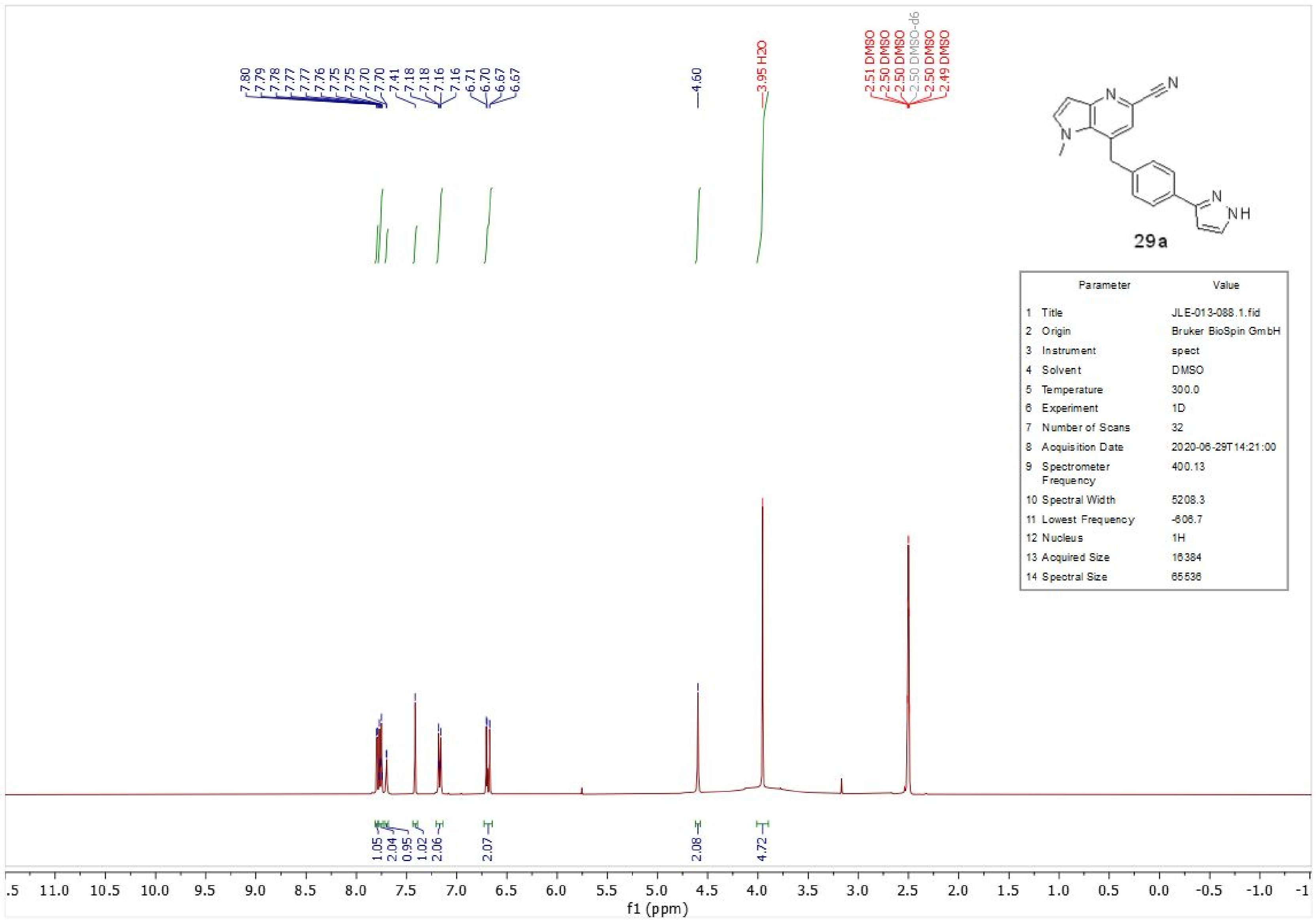

^{1}H NMR (400 MHz, DMSO) δ 7.79 (d, *J* = 3.3 Hz, 1H), 7.78 – 7.74 (m, 2H), 7.70 (d, *J* = 2.2 Hz, 1H), 7.41 (s, 1H), 7.20 – 7.14 (m, 2H), 6.69 (dd, *J* = 13.0, 2.7 Hz, 2H), 4.60 (s, 2H), 3.95 (s, 3H), NH proton not observable.

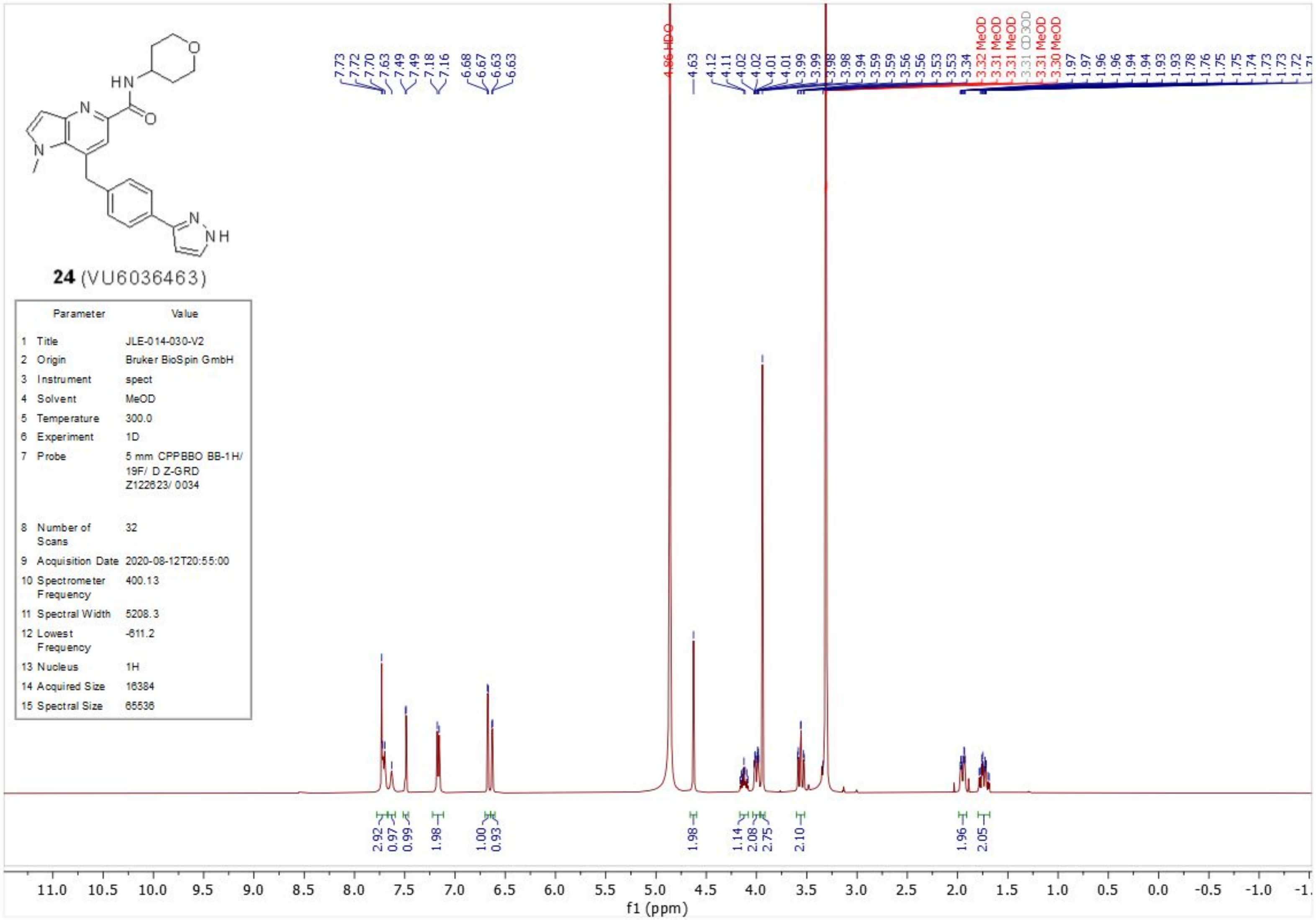

^{1}H NMR (400 MHz, MeOD) δ 7.73 – 7.70 (m, 3H), 7.63 (s, 1H), 7.49 (d, J = 3.3 Hz, 1H), 7.17 (d, J = 8.0 Hz, 2H), 6.67 (d, J = 3.3 Hz, 1H), 6.63 (d, J = 2.3 Hz, 1H), 4.63 (s, 2H), 4.12 (tt, J = 10.9, 4.3 Hz, 1H), 4.01 (dd, J = 8.2, 4.0 Hz, 2H), 3.94 (s, 3H), 3.56 (td, J = 11.7, 2.2 Hz, 2H), 2.00 – 1.87 (m, 2H), 1.74 (dtd, J = 13.0, 11.3, 4.4 Hz, 2H), N-H proton not observable.

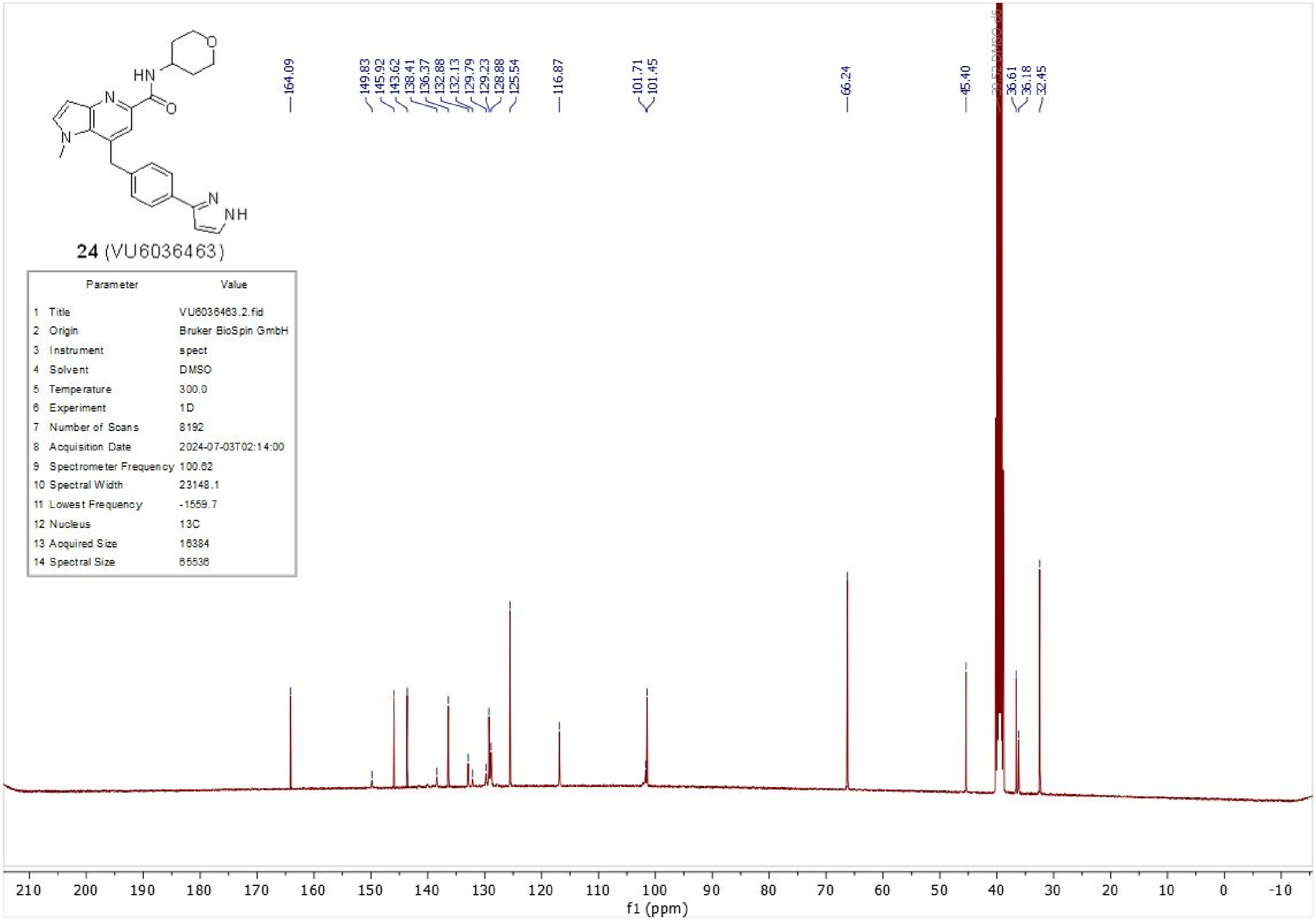

	Parameter	Value
1	Title	VU6036463.2.fid
2	Origin	Bruker BioSpin GmbH
3	Instrument	spect
4	Solvent	DMSO
5	Temperature	300.0
6	Experiment	1D
7	Number of Scans	8192
8	Acquisition Date	2024-07-03T02:14:00
9	Spectrometer Frequency	100.62
10	Spectral Width	23148.1
11	Lowest Frequency	-1559.7
12	Nucleus	13C
13	Acquired Size	16384
14	Spectral Size	65536

^{13}C NMR (101 MHz, DMSO) δ 164.09, 149.83, 145.92, 143.62, 138.41, 136.37, 132.88, 132.13, 129.79, 129.23, 128.88 (2C), 125.54 (2C), 116.87, 101.71, 101.45, 66.24 (2C), 45.40, 36.61, 36.18, 32.45 (2C).

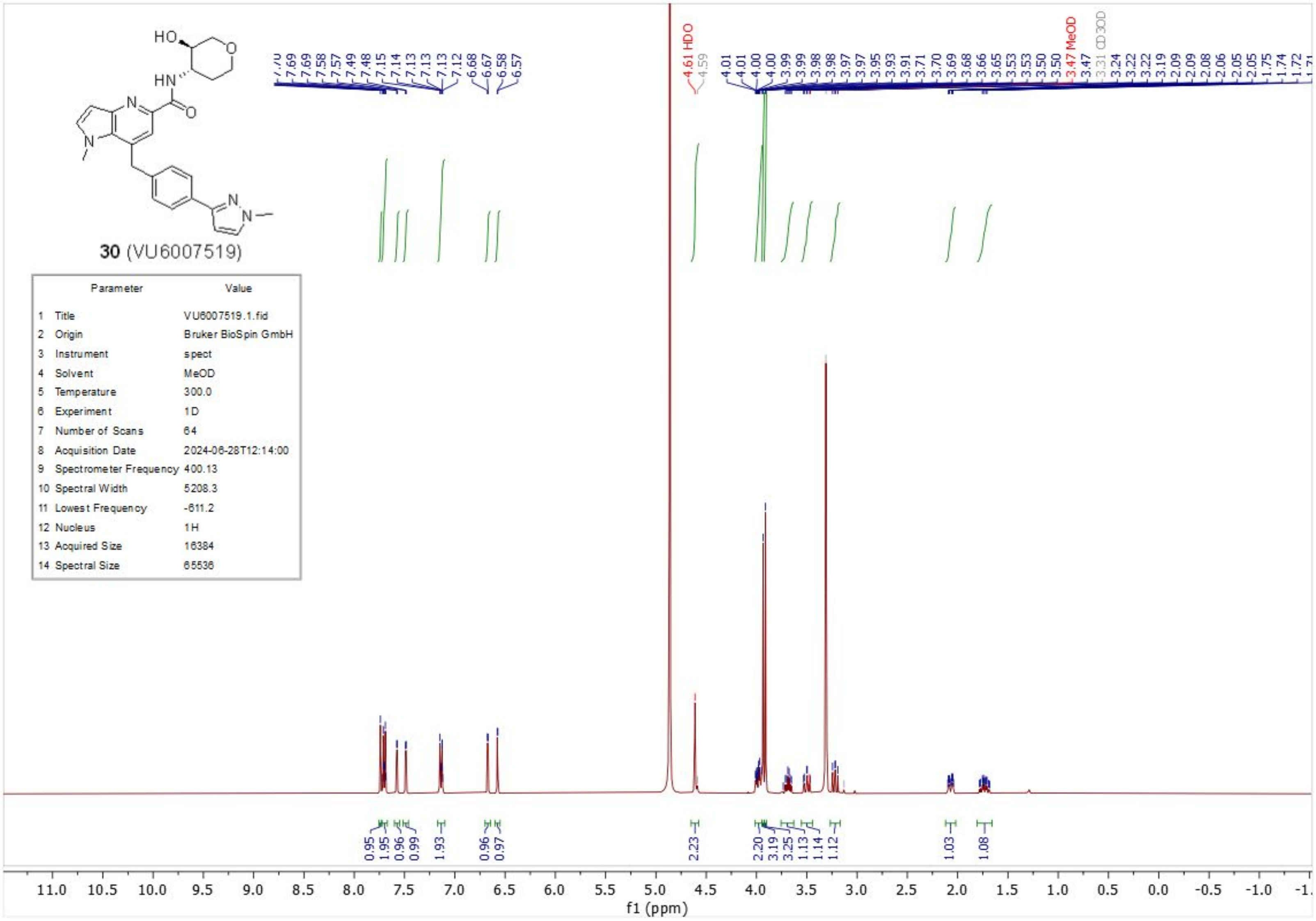

^{1}H NMR (400 MHz, MeOD) δ 7.74 (s, 1H), 7.72 – 7.68 (m, 2H), 7.58 (d, J = 2.3 Hz, 1H), 7.49 (d, J = 3.3 Hz, 1H), 7.17 – 7.10 (m, 2H), 6.67 (d, J = 3.3 Hz, 1H), 6.58 (d, J = 2.3 Hz, 1H), 4.61 (s, 2H), 4.02 – 3.93 (m, 6H), 3.91 (s, 3H), 3.68 (td, J = 9.6, 4.9 Hz, 1H), 3.54 – 3.47 (m, 1H), 3.22 (dd, J = 11.2, 9.8 Hz, 1H), 2.07 (ddt, J = 13.3, 4.6, 2.3 Hz, 1H), 1.73 (dtd, J = 13.3, 11.7, 4.6 Hz, 1H), N-H and O-H protons not observable.

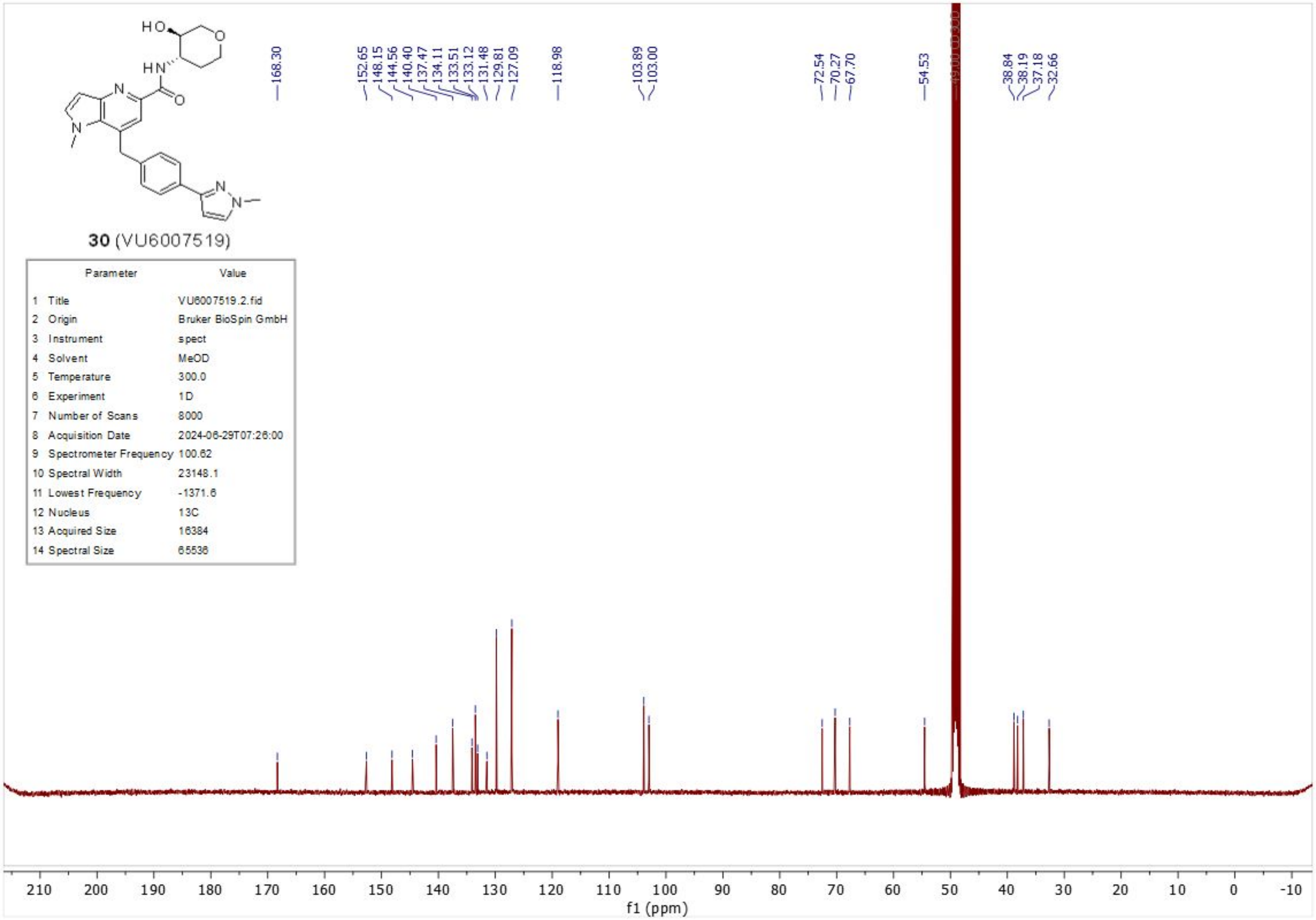

^{13}C NMR (101 MHz, MeOD) δ 168.30, 152.65, 148.15, 144.56, 140.40, 137.47, 134.11, 133.51, 133.12, 131.48, 129.81 (2C), 127.09 (2C), 118.98, 103.89, 103.00, 72.54, 70.27, 67.70, 54.53, 38.84, 38.19, 37.18, 32.66.

References

1. Wang, Q.; Rager, J. D.; Weinstein, K.; Kardos, P. S.; Dobson, G. L.; Li, J.; Hidalgo, I. J., Evaluation of the MDR-MDCK cell line as a permeability screen for the blood-brain barrier. *Int J Pharm* **2005**, *288* (2), 349-359.
2. Racine, R. J. Modification of seizure activity by electrical stimulation. II. Motor seizure. *Electroencephalogr. Clin. Neurophysiol*. **1972,** *32* (3) 281–294.
